# Drastic Enhancement of Activity and Durability for Oxygen Reduction Reaction by Melamine Modification for Platinum Nanocluster‐Loaded Electrocatalysts

**DOI:** 10.1002/smsc.202500632

**Published:** 2026-03-15

**Authors:** Ryuki Kurosaki, Tokuhisa Kawawaki, Kaoru Ikeda, Kazutaka Oiwa, Kotaro Sato, Haruna Tachibana, Minoru Inaba, Kenji Iida, Yuichi Negishi

**Affiliations:** ^1^ Carbon Value Research Center Research Institute for Science and Technology Tokyo University of Science Shinjuku‐ku Japan; ^2^ Institute of Multidisciplinary Research for Advanced Materials Tohoku University Sendai Japan; ^3^ Department of Molecular Chemistry and Biochemistry Doshisha University Kyoto Japan; ^4^ Institute for Catalysis Hokkaido University Sapporo Japan

**Keywords:** cluster, electrocatalyst, melamine, oxygen reduction, platinum

## Abstract

To facilitate the widespread adoption of environmentally friendly fuel cells, it is essential to enhance the mass activity (MA) and durability of platinum (Pt) catalysts used in the oxygen reduction reaction (ORR). In this study, approximately 1 nm Pt nanocluster (NC)‐loaded carbon black (CB) (Pt NC/CB) catalysts were successfully prepared. The obtained Pt NC/CB catalysts exhibited up to 3.25 times higher ORR MA than commercial Pt nanoparticle (NP)/CB catalysts. Furthermore, the melamine‐modified Pt NC/CB catalysts achieved up to 6.37 times higher MA and 2.5 times higher durability than commercial Pt NP/CB catalysts. Notably, after durability testing, the melamine‐modified Pt NC/CB catalysts achieved 11.0 times higher ORR MA than that of the commercial Pt NP/CB. Although small Pt NCs were considered to be less durable than Pt NPs owing to their high surface energy, the strong interaction between the Pt NCs and melamine significantly enhances their activity and durability. These findings offer a pathway to significantly reduce Pt loading in polymer electrolyte fuel cells, contributing to their practical and widespread application.

## Graphical abstract

We developed 1 nm platinum (Pt) nanoclusters whose mass activity and durability were enhanced 6.37 and 2.5 times, respectively, via melamine modification. After durability tests, activity remained 11.0 times higher than commercial catalysts. This strong Pt–melamine interaction stabilizes small clusters, offering a pathway to significantly reduce Pt usage and facilitate the widespread adoption of environmentally friendly polymer electrolyte fuel cells.

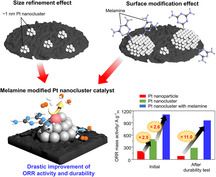

## 1 Introduction

Recently, the depletion of fossil fuel resources and the reduction of carbon dioxide (CO_2_) emissions have made it desirable to transition to a recycling‐oriented society that uses green hydrogen (H_2_) as an energy source. In particular, polymer electrolyte fuel cells (PEFCs; Figure S1) are attracting great attention as a next‐generation power source for homes and vehicles because they can produce electricity from H_2_ and oxygen (O_2_) in air without emitting CO_2_. The oxygen reduction reaction (ORR) occurring at the cathode in the PEFC is the rate‐limiting step, and a large amount of expensive and rare platinum (Pt) catalyst is used to reduce the activation energy. There are also durability issues during operation, resulting in aggregation and dissolution of the Pt catalyst. Consequently, PEFCs are not widely used. Therefore, it is essential to improve the activity per amount and durability of Pt used as a catalyst in the ORR for the wide spread use of PEFCs and to realize a clean next‐generation energy society.

**FIGURE 1:**
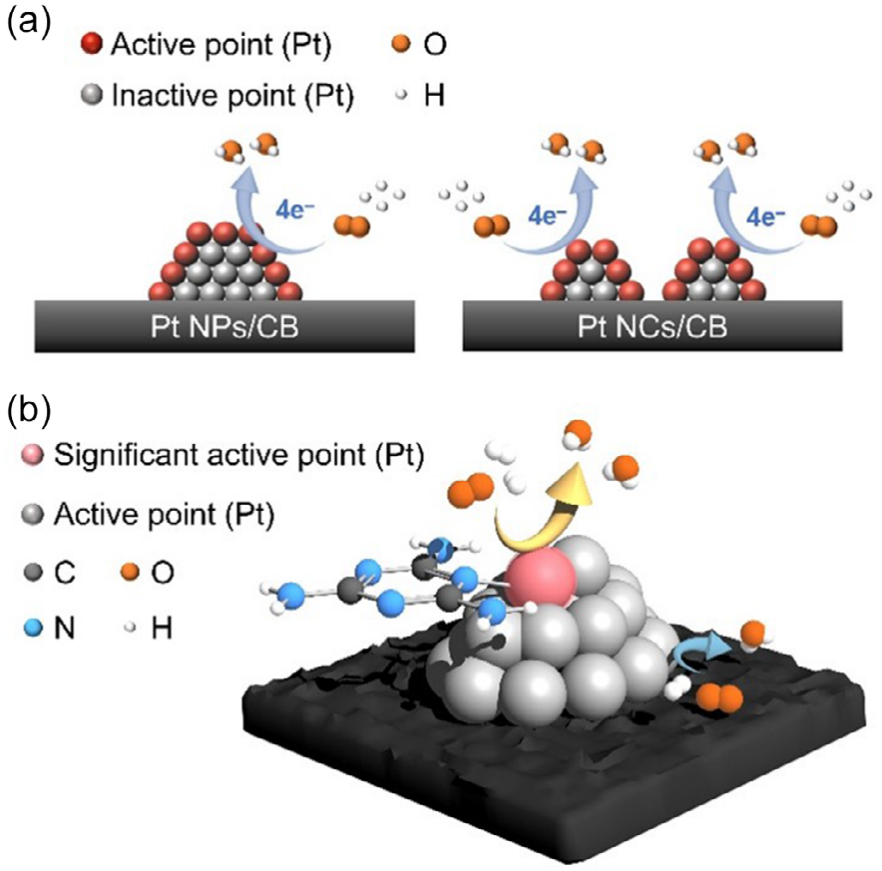
Size refinement effect of Pt nanoclusters (NCs) and melamine modification. (a) Schematics of the size refinement effects of Pt catalysts and (b) melamine modification effects on Pt catalysts for the improvement of the oxygen reduction reaction (ORR) activity.

In general, PEFCs are equipped with catalysts consisting of Pt nanoparticles (NPs) with diameters of approximately 3–5 nm on carbon black (CB). However, recent studies have revealed that approximately 1‐nm Pt nanocluster (NC)‐loaded CB (Pt NC/CB) catalysts exhibit higher ORR activity than Pt NP‐loaded CB (Pt NP/CB) catalysts [1, 2, 3]. The main reasons for this increase in the ORR activity are thought to be (1) an increase in the number of Pt atoms on the surface at which the ORR can proceed and (2) the formation of Pt atoms that are thermodynamically favorable for the ORR owing to the unique electronic structure of Pt NCs based on their small size (Figure 1a) [4]. From density functional theory (DFT) calculations, it has been found that Pt NCs on graphene have Pt atoms with electronic states favorable for the ORR, thus enhancing the ORR activity [5, 6]. Furthermore, it has recently been reported that modification of Pt NP/CB with certain organic compounds can improve the ORR mass activity (MA) and durability. In particular, the modification of Pt NP/CB and Pt alloy NP catalysts with melamine (Figure S2), a 1,3,5‐triazine ring with three terminal NH_2_ groups, significantly enhances the ORR activity [7, 8]. However, the origin of the increase of the ORR activity owing to melamine modification has not been clarified completely, and the effect of Pt particle miniaturization on these organic modifications has not been investigated.

In this study, we created ORR electrocatalysts that exhibit significantly higher activity than commercial Pt NP/CB catalysts by using Pt_*x*_ NCs with different numbers of Pt atoms (*x* = 17, ∼35, ∼51, and ∼66) as precursors. In addition, we modified the Pt_*x*_ NC CB‐supported catalysts with melamine to further improve their activity and durability. These catalysts achieved 6.37 times higher MA and 2.5 times better durability than commercial Pt NP/CB, especially by modifying the supported catalyst with melamine using Pt_∼51_ NCs as a precursor. In addition, DFT calculations were performed to elucidate the origins of the increases in these activities. The stronger adsorption of melamine on Pt NC/CB than on Pt NP/CB suggests that melamine modification has a greater effect on the ORR activity and durability for Pt NC/CB than Pt NP/CB (Figure 1b).

## 2 Results and Discussion

### 2.1 Improvement of the ORR Activity Using the Size Refinement Effects of Pt Catalysts

**FIGURE 2:**
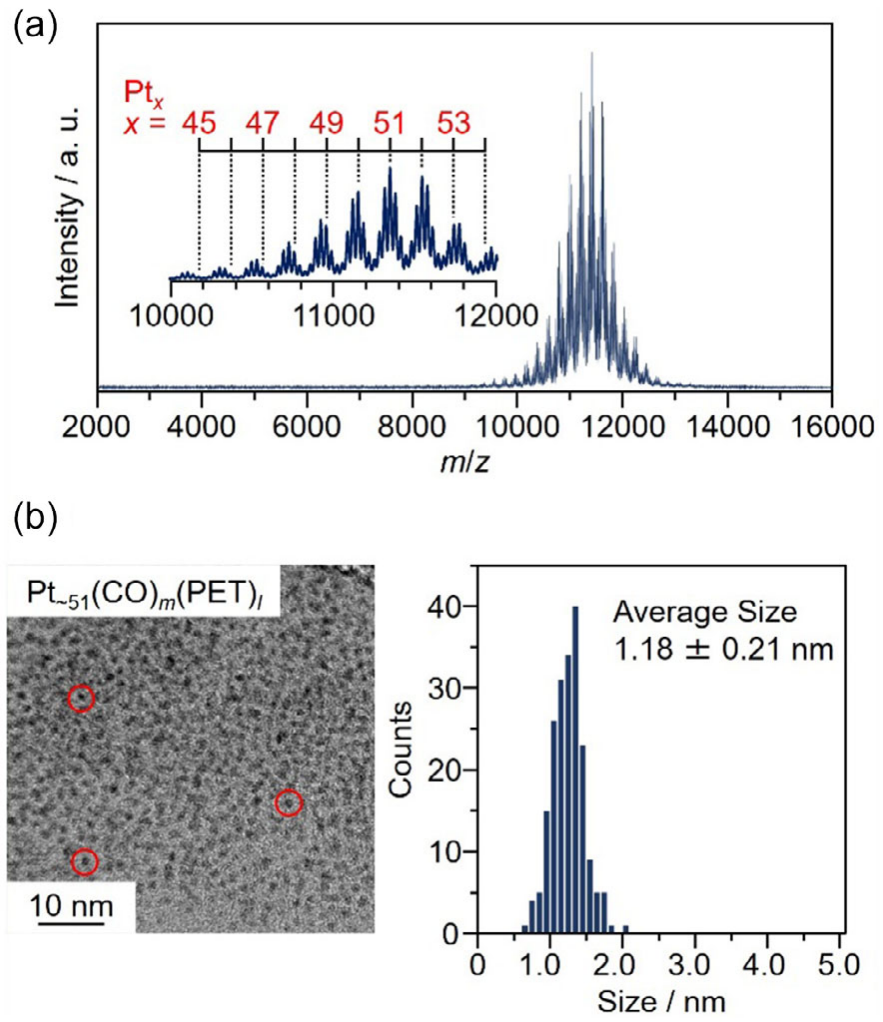
Size characterization of Pt NCs by mass spectrometry and particle size observation. (a) Matrix‐assisted laser desorption/ionization mass spectrum and (b) transmission electron microscopy (TEM) image and the resulting Pt size histogram of Pt_∼51_(CO)_*m*_(PET)_*l*_ (CO = carbon monoxide, PET = 2‐phenylethanethiolate).

**FIGURE 3:**
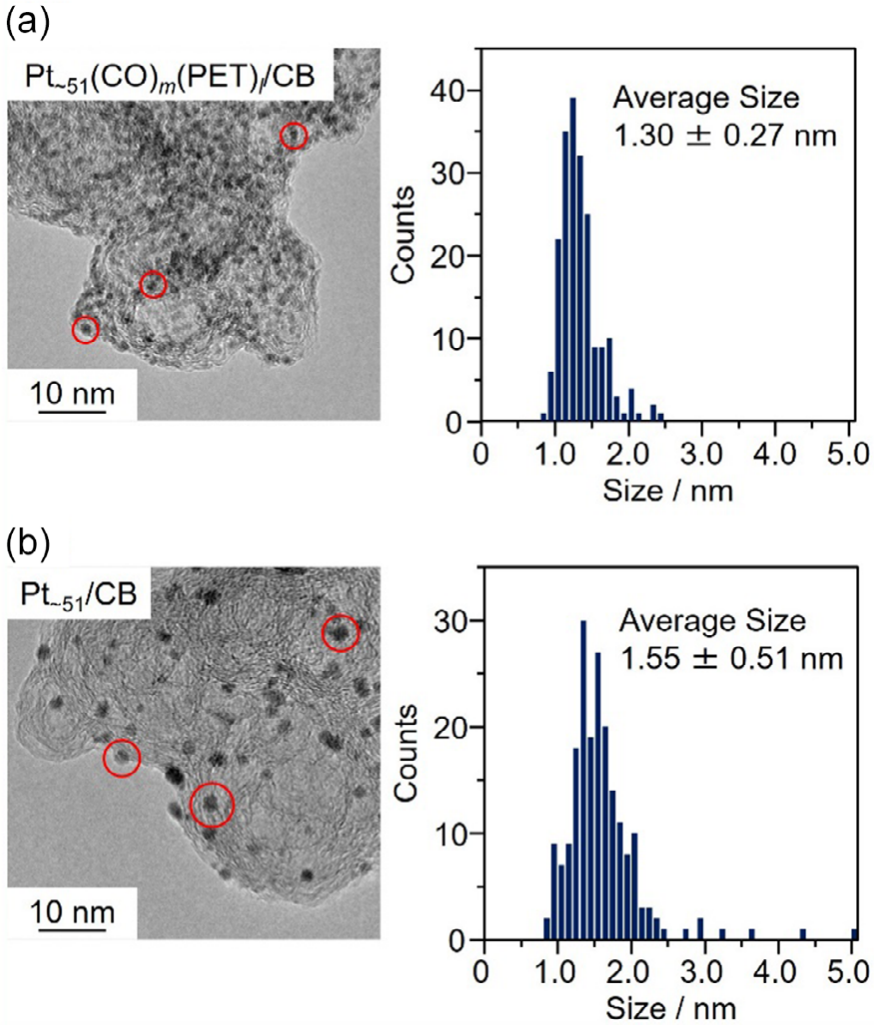
Size refinement effect of Pt NCs and melamine modification. (a) Schematics of the size refinement effects of Pt catalysts and (b) melamine modification effects on Pt catalysts for the improvement of the ORR activity.

**FIGURE 4:**
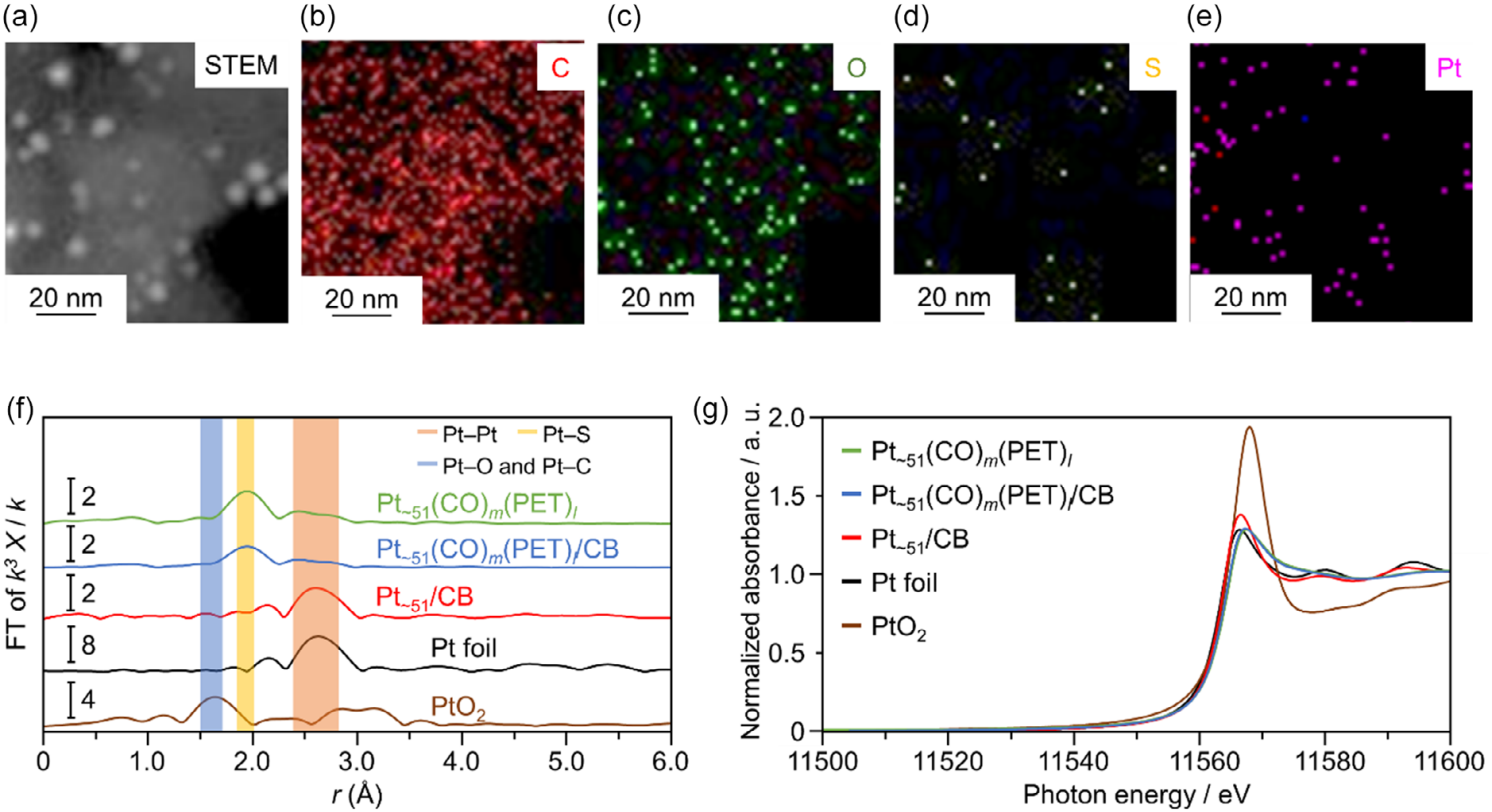
The change of bonding and charge state of Pt and different atoms during the catalyst conditioning process. (a) High‐angle annular dark‐field scanning transmission electron microscopy (STEM) images and energy‐dispersive X‐ray spectroscopy maps of (b) C‐K, (c) O‐K, (d) S‐K, and (e) Pt‐M of Pt_∼51_/CB. (f) Pt L_3_‐edge Fourier transform (FT)‐EXAFS and (g) X‐ray absorption near edge structure spectra of Pt_∼51_(CO)_∼12_(PET)_∼33_, Pt_∼51_(CO)_∼12_(PET)_∼33_/CB, and Pt_∼51_/CB. The spectra of Pt foil and PtO_2_ powder are also provided as references in (f) and (g).

**FIGURE 5:**
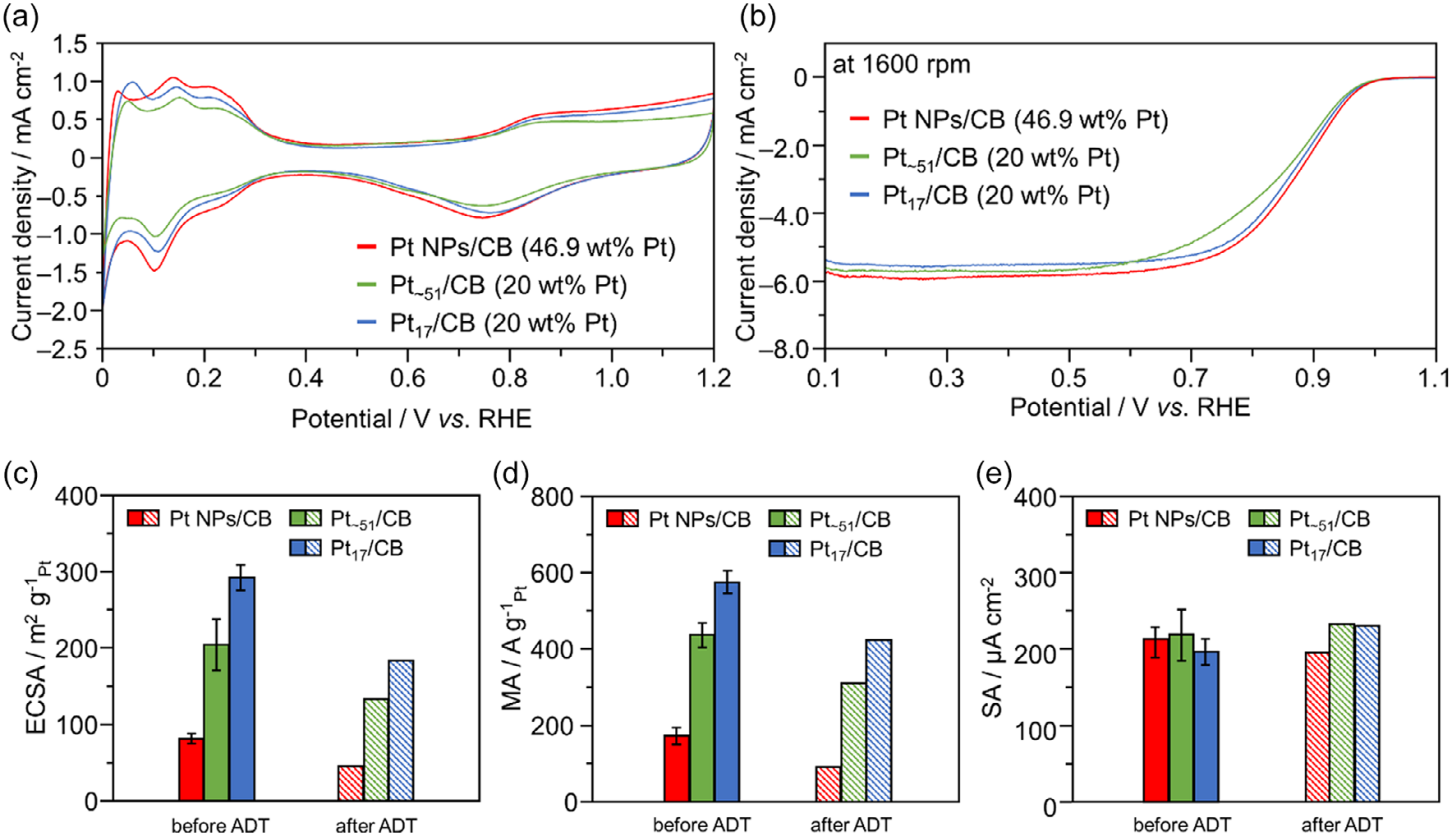
Electrochemical characterization of Pt catalyst for size dependece. Comparison of the (a) cyclic voltammograms (CVs) and (b) linear sweep voltammograms (LSVs). Resulting (c) electrochemical surfaces (ECSAs) using proton adsorption current from CVs, (d) mass activities (MAs) calculated using the Koutecký–Levich plots (Figure S19) from LSVs at 0.9 V versus reversible hydrogen electrode (RHE), and (e) specific activities (SAs) calculated from the ECSAs and MAs for Pt NP/CB, Pt_∼51_/CB, and Pt_17_/CB. In (c–e), ECSAs, MAs, and SAs of Pt NP/CB, Pt_∼51_/CB, and Pt_17_/CB after the accelerated durability test (ADT) calculated from the CVs, and Koutecký–Levich plots of LSVs (Figure S24) were also shown. The loading weights of Pt were 46.9, 20.0, and 20.0 wt% for Pt NP/CB, Pt_∼51_/CB, and Pt_17_/CB, respectively.

Based on previous reports [9, 10, 11, 12, 13, 14, 15, 16], we prepared Pt_*x*_ NC/CB catalysts (Figures 2–4, S3–13 and Scheme S1). The Pt particles of Pt_17_/CB (1.44 ± 0.34 nm) and Pt_∼51_/CB (1.55 ± 0.51 nm) were supported on CB with finer sizes than that of commercial Pt NP/CB (2.99 ± 0.50 nm). Such Pt_*x*_ NC/CB catalysts had a slightly cationic electronic state compared to metallic Pt and had an essentially different electronic structure that was discrete based on the quantum size effect. The detailed characterization of Pt_*x*_ NC/CB catalysts were shown in Method section. After preparing catalyst slurries of Pt NP/CB and the prepared Pt_*x*_/CB (*x* = 17, ∼35, ∼51, and ∼66), the Pt‐supported catalysts were used to form working electrodes (Scheme S2 and Figure S14). Next, a three‐electrode system (Figure S15) was assembled in 0.1 M perchloric acid, and electrochemical cleaning [17] (Figure S16) was performed to remove the ligands remaining on the catalyst surface after the calcination process. Aggregation of the Pt NCs was not observed in transmission electron microscopy (TEM) images of the Pt NCs after the cleaning process (Figure S17). Next, cyclic voltammetry and linear sweep voltammetry were performed under the experimental conditions of the unified standard in Japan (Figure 5a,b) [18]. Using these results, the electrochemical surface area (ECSA) based on proton (H^+^) adsorption, the MA, and the specific activity (SA) were calculated (Figure 5c–e).

The cyclic voltammograms (CVs) of the Pt‐supported catalysts under a nitrogen (N_2_) atmosphere are shown in Figure 5a. No significant differences in the CV shapes of the Pt catalysts were observed. Additionally, because Pt NP/CB had higher Pt loading than Pt_*x*_/CB (*x* = 17 and ∼51) (46.9 vs. 20.0 wt% Pt, respectively), a larger current associated with H^+^ adsorption was observed for Pt NP/CB. The Pt mass‐specific ECSAs calculated from the currents associated with H^+^ adsorption are shown in Figure 5c. The ECSA of Pt NP/CB was 81.2 m^2^/g_Pt_ (Figure 5c), which is in agreement with values reported in the literature [19, 20]. This indicates that the ORR activity evaluation method is appropriate. Using a similar method, the ECSA of each Pt_*x*_/CB was calculated from the CV results (Figure 5c). Pt_∼51_/CB and Pt_17_/CB exhibited ECSAs of 204 and 292 m^2^/g_Pt_, respectively. These ECSAs are extremely high, and they were 2.51 and 3.60 times higher than that of Pt NP/CB. The miniaturization of the Pt particles increased their specific surface area, which in turn probably led to a significant increase in the number of surface Pt atoms that can act as active sites, resulting in a substantial increase of the ECSA.

The linear sweep voltammogram (LSV) of each Pt CB‐supported catalyst under an O_2_ atmosphere (at a rotation speed of 1600 rpm) is shown in Figure 5b. Despite Pt_*x*_/CB having lower Pt loading than Pt NP/CB (20.0 vs. 46.9 wt% Pt, respectively), a similar onset voltage for the ORR current rise to Pt NP/CB was observed. The MA of each Pt CB‐supported catalyst at 0.9 V versus reversible hydrogen electrode (RHE) calculated from the LSVs measured at various rotation speeds (Figures S18 and S19) is shown in Figure 5d. The MA of Pt NP/CB was 172.3 A/g_Pt_ at 0.9 V versus RHE, which is consistent with values ranging from ∼180 A/g_Pt_ as reported in the literature [19, 20]. This confirmed that, like for the ECSA, the ORR activity evaluation method is appropriate. The MAs of the Pt NC CB‐supported catalysts were 437.0 A/g_Pt_ for Pt_∼51_/CB and 561.6 A/g_Pt_ for Pt_17_/CB (Figure 5d). These values represent extremely high activity, and the MAs of Pt_∼51_/CB and Pt_17_/CB were 2.54 and 3.25 times higher than that of the commercial Pt NP/CB catalyst, respectively. Furthermore, the SA of each catalyst was calculated by dividing the MA by the ECSA (Figure 5e). Although Pt_∼51_/CB and Pt_17_/CB exhibited slightly higher SAs than Pt NP/CB, the values were almost the same. These results suggest that the improvement in the activity owing to the miniaturization of the Pt particles is largely caused by two key factors. First, the improvement in the Pt specific surface area is mainly caused by the particle size reduction [21, 22, 23]. As a result, it is believed that the number of Pt surface atoms involved in the ORR significantly increases (Figure 1b). Second, the miniaturization process generates surface Pt atoms with various charge states, which are considered to act as active sites for the ORR [4, 24, 25, 26]. By previous DFT calculations [27], we revealed that the Pt atoms in Pt_17_/CB exhibit various charge states, providing favorable active sites for the ORR. This makes ORR progression easier compared with that with the Pt(111) surface of Pt NP/CB (Figure S20). From these, it is considered that Pt_∼51_/CB and Pt_17_/CB exhibit higher activity than Pt NP/CB because of (1) an increase in the number of active sites through miniaturization and (2) the presence of active sites at which the ORR can more easily proceed.

To evaluate the durability of the catalysts, an accelerated durability test (ADT) was performed under experimental conditions based on the unified standards in Japan [18] (Figures S21–S24). The ECSA and MA of each Pt CB‐supported catalyst before and after the ADT are shown in Figure 5c,d. Pt_∼51_/CB and Pt_17_/CB showed higher ECSAs and MAs after the ADT than Pt NP/CB before the ADT. The decrease rates were 31.2% for Pt_17_/CB and 33.3% for Pt_∼51_/CB, which were smaller than that of Pt NP/CB (50.8%). In general, the ADT promotes Ostwald ripening of Pt particles, which encourages their aggregation and leads to a decrease in the specific surface area, resulting in a decrease in the MA. It is considered that small Pt particles are more likely to aggregate owing to their high surface energy [11], but it is speculated that the aggregation of the Pt particles in Pt_*x*_/CB was suppressed by the strong interactions with the remaining ligands and support. From the Pt particle sizes in TEM images of the catalysts after the ADT, while the Pt particle sizes increased after the ADT for all of the catalysts (2.95 ± 0.57–5.44 ± 1.62 nm for Pt NP/CB, 1.50 ± 0.44–3.24 ± 1.16 nm for Pt_∼51_/CB, and 1.66 ± 0.51–3.09 ± 1.38 nm for Pt_17_/CB (Figures S17 and S25), the Pt particles in Pt_*x*_/CB (*x* = 17 and ∼51) remained smaller than those in Pt NP/CB even after the ADT. It is speculated that the suppression of the Pt particle aggregation led to smaller decreases in the ECSAs and MAs for Pt_*x*_/CB (*x* = 17 and ∼51) than Pt NP/CB during the ADT. Regarding SA (Figure 5e), a slight improvement was observed due to the relatively suppressed MA decrease rate in the ADT for the Pt_17_/CB. This suggests that the Pt particles in Pt_*x*_/CB and Pt NP/CB exhibit different degradation processes.

### 2.2 Improvement of the ORR Activity and Durability of the Pt NC/CB Catalysts by Melamine Modification

In recent years, it has been reported that modifying the surface of Pt NP/CB with melamine can significantly enhance the ORR activity [28, 29, 30, 31, 32] by suppressing an adsorption of H_2_O and the resulting formation of –OH and –O [30, 31, 32]. In this study, to further improve the ORR activity and durability of Pt_*x*_ NC/CB and to elucidate the factors that contribute to the enhancement of their activity, appropriate organic compounds were added to Pt_*x*_ NC/CB.

**FIGURE 6:**
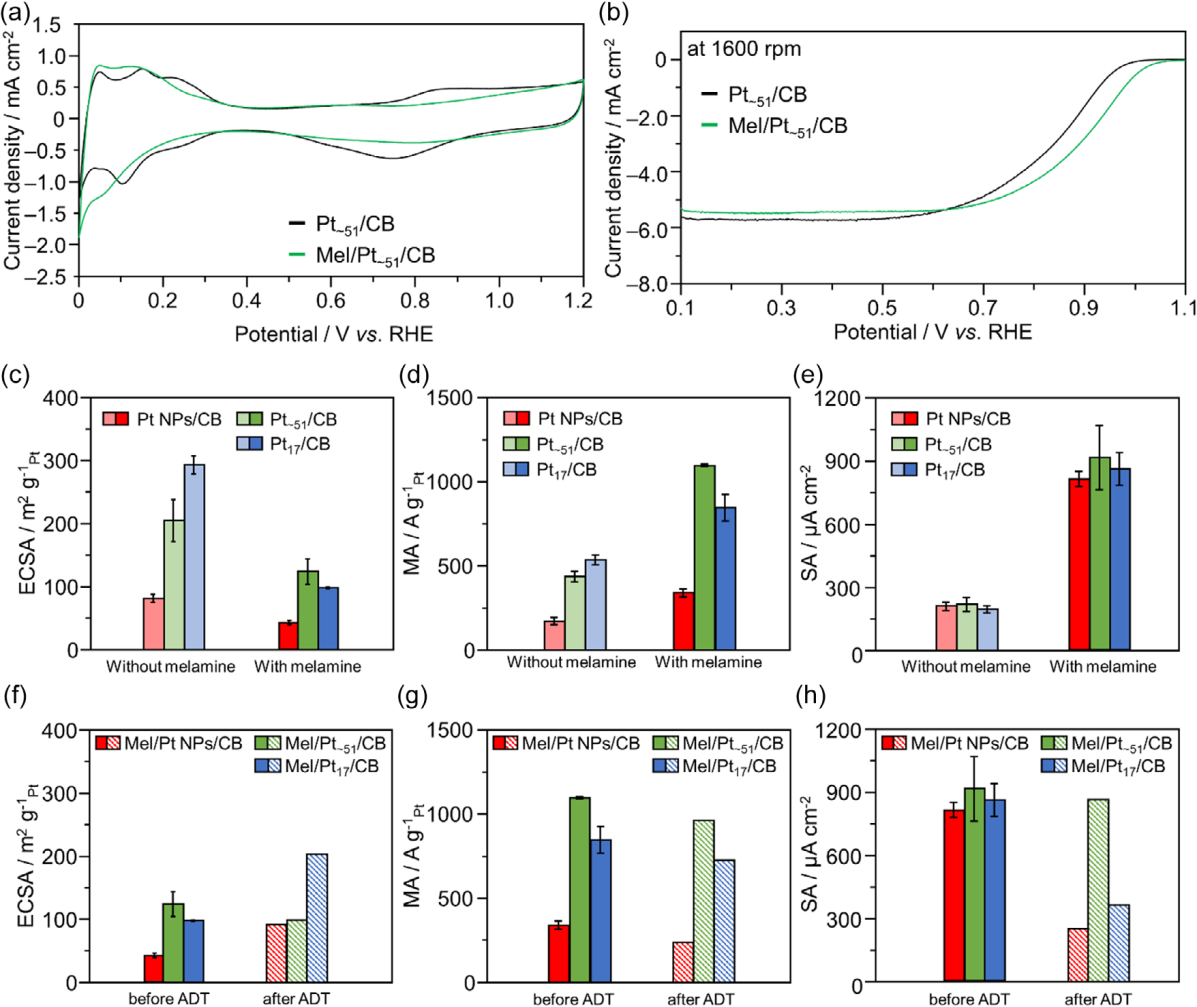
Electrochemical characterization of Pt catalyst for melamine modification effect. Comparison of the (a) CVs and (b) LSV of Pt_∼51_/CB before and after melamine modification. Resulting (c) ECSAs, (d) MAs, and (e) SAs of Pt NP/CB, Pt_∼51_/CB, and Pt_17_/CB before and after melamine modification. Resulting (f) ECSAs, (g) MAs, and (h) SAs of Mel/Pt NP/CB, Mel/Pt_∼51_/CB, and Mel/Pt_17_/CB before and after the ADT. In (b), LSVs were obtained at 1600 rpm of a rotation speed. In (c,f), ECSAs were obtained using proton adsorption current from the CVs. In (d,g), MAs were calculated from the Koutecký–Levich plots. The loading weights of Pt were 46.9, 20.0, and 20.0 wt% for Mel/Pt NP/CB, Mel/Pt_∼51_/CB, and Mel/Pt_17_/CB, respectively.

Pt NP/CB and Pt_*x*_/CB modified with melamine are referred to as Mel/Pt NP/CB and Mel/Pt_*x*_/CB, respectively (Scheme S3). The adsorption of melamine onto these catalyst surfaces was also confirmed by FT‐IR and Pt L_3_‐edge extended X‐ray absorption fine structure (EXAFS) spectra (Figures S13 and S26). Electrochemical measurements were similarly performed using a melamine‐protected Pt catalyst (Figure 6). From the CV results of each Pt‐supported catalyst (Figure 6a and S27), melamine modification led to a decrease in H^+^ adsorption, that is, a reduction in the ECSA (Figure 6c). A similar decrease in the ECSA has been reported for melamine‐modified Pt NP/CB [30]. This suggests that the Pt particle surface is protected by melamine, inhibiting H^+^ adsorption. Furthermore, the onset of the oxidation current based on Pt–OH formation at 0.7 V versus RHE shifted to a more positive potential after melamine modification, suggesting that the excessive formation of Pt–OH was suppressed [31]. Conversely, from the LSVs under an O_2_ atmosphere (Figure 6b, and Figures S28 and S29), for all of the Pt‐supported catalysts, the onset of the reduction current based on the ORR shifted to a more positive potential, and the overpotential was suppressed. The MA at 0.9 V versus RHE significantly improved for all of the Pt‐supported catalysts after melamine modification (Figure 6d). For Pt NP/CB, melamine modification resulted in a 1.97‐fold increase in the MA (from 172.3–340.2 A/g_Pt_), which is consistent with a previous study [30]. Pt_*x*_ NC/CB showed a significantly better improvement in the MA than Pt NP/CB after melamine modification. In particular, Mel/Pt_∼51_/CB exhibited a MA of 1098 A/g_Pt_, which was approximately 2.51 times higher than that of Pt_∼51_/CB (Figure 6d). As a result, Mel/Pt_∼51_/CB achieved 6.37 times higher MA than the commercial Pt NP/CB catalyst. The SAs of all of the catalysts significantly improved, similar to previously reported studies [7, 8, 29, 30, 32], in conjunction with the decrease in the ECSA and the increase in the MA (Figure 6e). Furthermore, it was confirmed that the ORR proceeds via the ideal four‐electron reaction for all catalysts (Table S1).

Next, to investigate the effect of melamine modification on the durability of the Pt catalysts, we also performed ADT using the melamine‐modified Pt CB‐supported catalysts. Generally, in the ADT, the degradation of the Pt catalyst is evaluated by measuring the ECSA after a certain number of overloading cycles. However, because melamine modification induces significant suppression of the ECSA owing to H^+^ adsorption, we considered that it was important to compare not only the ECSA, but also the MA. The ECSAs and MAs of Mel/Pt_∼51_/CB and Mel/Pt NP/CB before and after the ADT are shown in Figure 6f,g and Figures S30–S32. The MA of Mel/Pt NP/CB decreased by 32.3% during the ADT (from 350.4–237.4 A/g_Pt_). As previously mentioned, for Pt NP/CB without melamine modification, the MA decreased by 50.8% after the same number of ADT cycles. Similar to a previous study [30], melamine modification resulted in a slight improvement in the durability (from 50.8% to 32.3% decrease rate). Surprisingly, the MA of Mel/Pt_∼51_/CB decreased by only 12.8% after the ADT (Figure 6g; from 1105 to 964.0 A/g_Pt_). This value represents a 2.5‐times smaller MA decrease rate than that of Mel/Pt NP/CB (32.3% vs. 12.8%). This indicates that melamine modification of Pt NC CB‐supported catalysts not only improves their MA, but it also significantly improves their durability (Figure 6g). The MA of Mel/Pt_∼51_/CB after the ADT was 11.0 times higher than that of commercial Pt NP/CB (87.5 vs. 964.0 A/g_Pt_).

**FIGURE 7:**
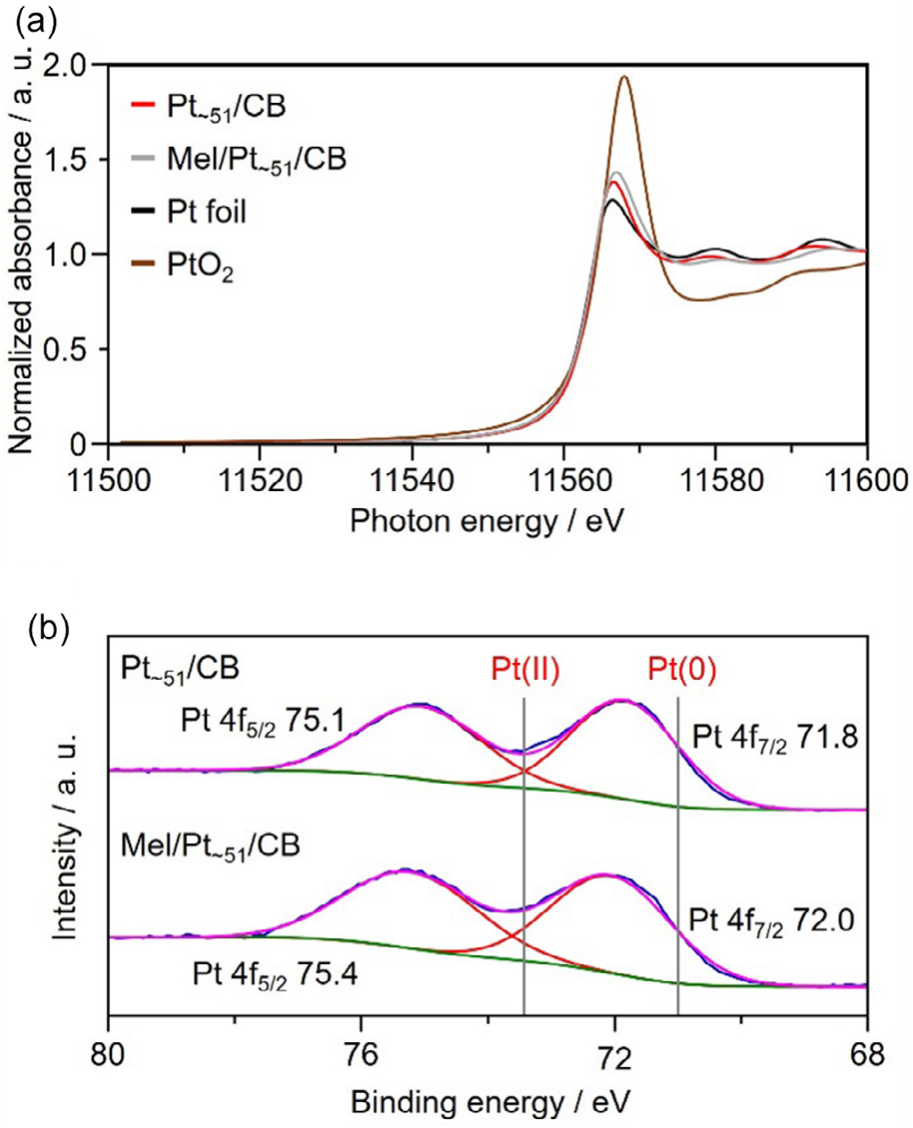
Change in charge state of Pt by melamine modification. (a) Pt L_3_‐edge X‐ray absorption near edge structure and (b) Pt 4f X‐ray photoelectron spectra of Pt_∼51_/CB before and after modification with melamine. In (a), the results of Pt foil and PtO_2_ powder are also shown for comparison. These changes in the electronic states were observed under conditions of forced excess adsorption of melamine. At the ultratrace adsorption levels associated with high activity, these signals remained below the detection limit of the current measurement system.

This large difference in the durability is presumed to be because of the stronger adsorption ability of melamine on the Pt NCs than on the Pt NPs. Therefore, the adsorption ability of melamine was evaluated by the ECSA obtained from the CVs before and after the ADT (Figure 6f and Figure S32). For Mel/Pt NP/CB, a significant increase in the ECSA was observed after approximately 2500 cycles of the ADT. This is probably because of the re‐exposure of surface Pt atoms following the desorption of melamine. From this, it can be inferred that melamine is relatively weakly coordinated to the Pt NPs, and that the adsorbed melamine gradually desorbed owing to the overloading during the ADT. This trend has also been observed in a previous study [31]. In contrast, Mel/Pt_∼51_/CB maintained almost the same ECSA even after 10 500 cycles of the ADT (Figure 6f). Significant differences in SAs were observed due to melamine adsorption strength. While Mel/Pt_∼51_/CB maintained its uniquely high SA attributable to melamine adsorption even after ADT, SA decreased substantially in Mel/Pt NP/CB and Mel/Pt_17_/CB (Figure 6h). This suggests that melamine is relatively strongly attached to the Pt particles of Pt_∼51_/CB, and melamine desorption did not occur even under the overloading conditions of the ADT (Figure S33). Therefore, the change in the electronic state of the Pt particles due to melamine coordination was evaluated (Figure 7). From the results of Pt L_3_‐edge X‐ray absorption near‐edge structure (XANES; Figure 7a and Figure S34), Mel/Pt_∼51_/CB showed a higher peak intensity for the white line at 11 568 eV than Pt_∼51_/CB, indicating that the electronic state of Pt shifted to a more positive state (Figure 7a). Furthermore, the magnitude of this shift was larger than that of Pt NP/CB before and after melamine modification (Figure S34a). This indicates that melamine is more strongly adsorbed to Pt_∼51_/CB than Pt NP/CB, leading to stronger electron withdrawal from the Pt particles. Based on the above results, it is believed that melamine binds to the Pt atoms of Pt_∼51_/CB and causes changes in their electronic state, which in turn enhances the ORR activity [33, 34]. Melamine modification is expected to suppress the movement of the Pt particles on CB owing to temperature‐ or potential‐induced fluctuations, thus preventing their aggregation [31, 35]. The sizes of the Pt particles before and after the ADT were determined from TEM images (Figure S35). For Mel/Pt NP/CB (2.99 ± 0.50–3.21 ± 0.69 nm), the increase in the Pt particle size due to aggregation was slightly suppressed compared with that for Pt NP/CB (2.99 ± 0.50–5.44 ± 1.62 nm). The Pt particle size of Mel/Pt_∼51_/CB after the ADT was 1.89 ± 0.74 nm (from 1.55 ± 0.51 nm), showing significant suppression of the increase in the Pt particle size. This represents remarkable suppression of aggregation, especially when compared with the change in the Pt particle size for Pt_∼51_/CB without melamine modification before and after the ADT (1.55 ± 0.51–3.24 ± 1.16 nm). After the ADT, the Pt particle sizes of Pt NP/CB and Mel/Pt_∼51_/CB were 5.44 ± 1.62 and 1.89 ± 0.74 nm, respectively. This significant suppression of the Pt particle aggregation suggests that even after the ADT, Mel/Pt_∼51_/CB was able to maintain a significantly higher MA than commercial Pt NP/CB.

### 2.3 Size Effects of the Pt NC/CB Catalysts

**FIGURE 8:**
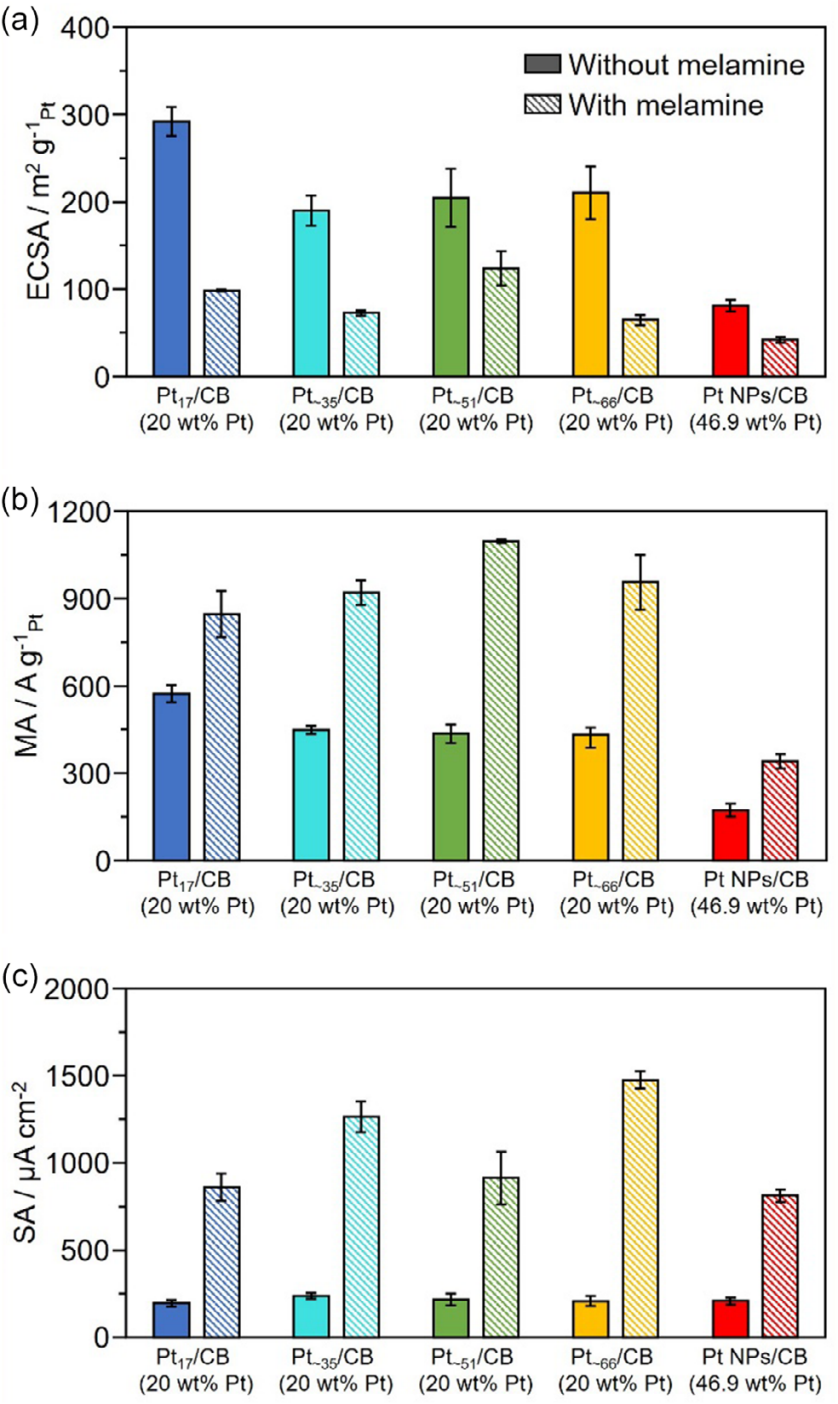
Effect of size and number of constituent atoms on ORR activity. (a) Comparison of the ECSAs, (b) MAs, and (c) SAs of Pt_*x*_/CB (*x* = 17, ∼35, ∼51, and ∼ 66) and Pt NP/CB with/without melamine modification. The loading weights of Pt were 46.9, and 20.0 wt% for Pt NP/CB and Pt_*x*_/CB, respectively.

The size dependence of the ECSA and MA was investigated using Pt_*x*_ NC/CB (*x* = 17, ∼35, ∼51, and ∼ 66) and Pt NP/CB. In addition to the previously discussed Pt_∼51_/CB, Pt_17_/CB, and Pt NP/CB, the MAs, ECSAs, and SAs of Pt_∼35_ NC/CB and Pt_∼66_ NC/CB before and after melamine modification are summarized in Figure 8.

Compared with Pt NP/CB, all of the Pt_*x*_/CB catalysts exhibited higher ECSAs, which is believed to be because of the increase in the number of Pt surface atoms resulting from the reduction in the particle size. The MA was also higher for smaller Pt_*x*_ NCs, Pt_17_/CB exhibited the maximum MA of 561.6 A/g_Pt_, which was 3.25 times higher than that of Pt NP/CB (172.3 A/g_Pt_). This improvement in the MA is believed to be because of mainly (1) the increase in the number of active sites resulting from the miniaturization of the particles and (2) the presence of active sites that facilitate the ORR, as previously mentioned. The lack of significant difference in SA among Pt‐loaded catalysts of each size also suggests that the main factor behind the increase in MA with size reduction is (1) the increase in surface Pt atoms.

In contrast, for the melamine‐modified Pt_*x*_/CB (Mel/Pt_*x*_/CB) catalysts, a slight change in the size dependence was observed. The highest MA among the Mel/Pt_*x*_/CB catalysts was observed for Mel/Pt_∼51_/CB (1098 A/g_Pt_), which was approximately 2.51 times higher than that of Pt_∼51_/CB. For Pt_17_/CB, which showed the highest activity without melamine modification, the MA only increased by approximately 1.51 times by melamine modification (from 561.6–845.4 A/g_Pt_). As previously mentioned, the important factors for the melamine modification effect on the ORR are believed to be based on (1) the coordination ability of melamine to the Pt particles and (2) the charge transfer interaction between the Pt particles and melamine. As the particle size decreases, the number of coordinatively unsaturated Pt atoms increases and melamine adsorption occur more preferentially on such Pt atoms. In this case, steric hindrance between melamine molecules may occur in fine Pt NCs. The reason why the melamine effect was smaller on Pt_17_/CB than on Pt_∼51_/CB might be that further miniaturization of Pt NCs reduced the adsorption coverage of melamine, suppressing the activity improvement effect. Additionally, for Pt NCs with a particle size of ≤2 nm, because the electronic/geometrical structure does not uniformly change with the size, it is believed that there are Pt NCs with specific electronic/geometrical structures that exhibit exceptionally high melamine modification effects. From the Pt L_3_‐edge XANES spectrum (Figure 7a), Pt_∼51_/CB showed a significantly larger change in the peak intensity of the white line with melamine modification than Pt NP/CB (Figure S34a) and Pt_17_/CB (Figure S34b), which is believed to have led to the exceptionally high melamine modification effect. Although there was some variation, the Mel/Pt_*x*_/CB showed a higher SA than the Mel/Pt NPs/CB. It indicates that the melamine effect in the NC‐loaded catalyst is obviously higher than that in the NPs‐loaded catalyst. Furthermore, such differences of SA were even more pronounced after the durability test (Figure 6h).

Unfortunately, for the current Pt_*x*_/CB catalysts, aggregation of some of the Pt particles occurred during the calcination process, making it difficult to perform more detailed investigations. It is expected that further improvement in the ORR activity can be achieved by the melamine modification effect through more precise control of the Pt NC size on CB.

### 2.4 Understanding the Melamine Modification Effects of the Pt NC/CB Catalysts

Previous studies have found that melamine adsorbed on the Pt(111) surface, as in Pt NPs, can enhance the ORR activity by (1) inhibiting the supply of water molecules as a source for Pt oxide species, thus preventing excessive stabilization of Pt–OH [31, 32] and (2) blocking the adsorption of toxic species such as sulfate ions (Figure S36) [29].

**FIGURE 9:**
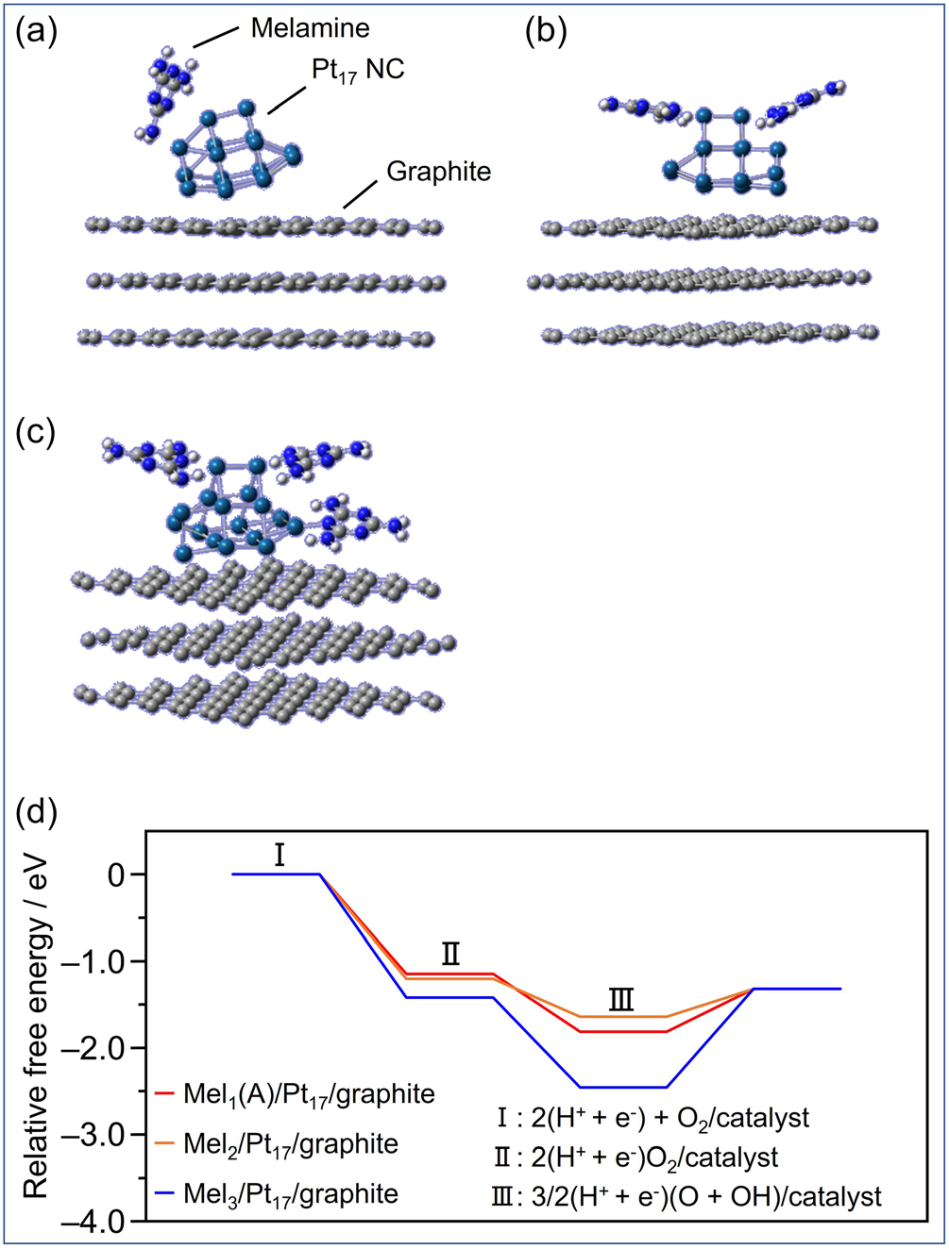
Results of density group function theory calculation. Optimized structures for (a) Mel_1_(A)/Pt_17_/graphite, (b) Mel_2_/Pt_17_/graphite, and (c) Mel_3_/Pt_17_/graphite. (d) Free‐energy diagrams of the ORR through a direct four‐electron pathway under the potential of 0.9 V versus standard hydrogen electrode.

However, since small Pt NCs exhibit electronic properties distinct from bulk Pt, it remains unclear if this phenomenon persists at the cluster scale. Thus, DFT calculations of Pt_17_ NC as the model for the NC were performed using the Quantum Espresso package. To reduce computational costs, the structure of the carbon support was modeled as graphite with a well‐defined geometric structure instead of CB [36], and the ligand eliminated Pt_17_ NC was used. In this process, the initial structure was based on the Pt_17_ NC on the support, which has been previously observed by our group using high‐angle annular dark‐field‐STEM [37, 38]. Structural optimization was then performed to obtain the optimized Pt_17_/graphite structure (Figure S37a–c). In a previous study [27], we demonstrated that (1) a Pt_17_ NC on graphite possesses multiple active sites (Figure S37d) and (2) under operation at 0.9 V versus standard hydrogen electrode (SHE) during the ORR, each reaction pathway of specific Pt sites solely consists of downhill reactions, resulting in high ORR activity (Figure S20). Conversely, the Pt(111) surface of Pt NPs includes uphill reactions within its reaction pathways. Using these models, we investigated the effects of melamine modification (Figure 9).

Structural optimization of Pt_17_/graphite after melamine adsorption revealed that several stable structures exist in which melamine is adsorbed to Pt atoms of Pt_17_ (Figure 9a–c, and Figures S38 and S39). Additionally, to investigate the effect of the number of melamine molecules adsorbed on Pt_17_/graphite, structural optimization was performed for stable structures with one to three adsorbed melamine molecules (Mel_*x*_; *x* = 1–3). Next, using each stable structure of melamine‐modified Pt_17_/graphite (Mel_*x*_(*X*)/Pt_17_/graphite; *x* = 1–3), the free energy diagrams of the ORR at 0.9 V versus SHE were calculated, and the results are shown in Figure 9d and Figure S40. In some of the Pt_17_/graphite structures with one or two adsorbed melamine molecules (Mel_1_(A)/Pt_17_/graphite and Mel_2_/Pt_17_/graphite; Figure 9a,b), the (OH + H)/Mel_*x*_(*X*)/Pt_17_/graphite configuration becomes destabilized compare with Mel_3_/Pt_17_/graphite. In such Mel_*x*_(*X*)/Pt_17_/graphite structures, it is inferred that the ORR is more easily facilitated owing to the smaller uphill energy from (OH + H)/Mel_*x*_(*X*)/Pt_17_/graphite (step III) to H_2_O/Mel_*x*_(*X*)/Pt_17_/graphite (step IV).

Theoretical calculations for heterogeneous catalytic reactions are inherently sensitive to the assumed atomic configurations of active sites, interfacial structures, and defect distributions. Therefore, while such pathways may not be universal across all NC catalysts, melamine modification could potentially enhance ORR activity in Pt NCs by facilitating a reaction pathway.

### 2.5 Origin of the Melamine Modification Effects of Pt NC/CB Catalysts

**FIGURE 10:**
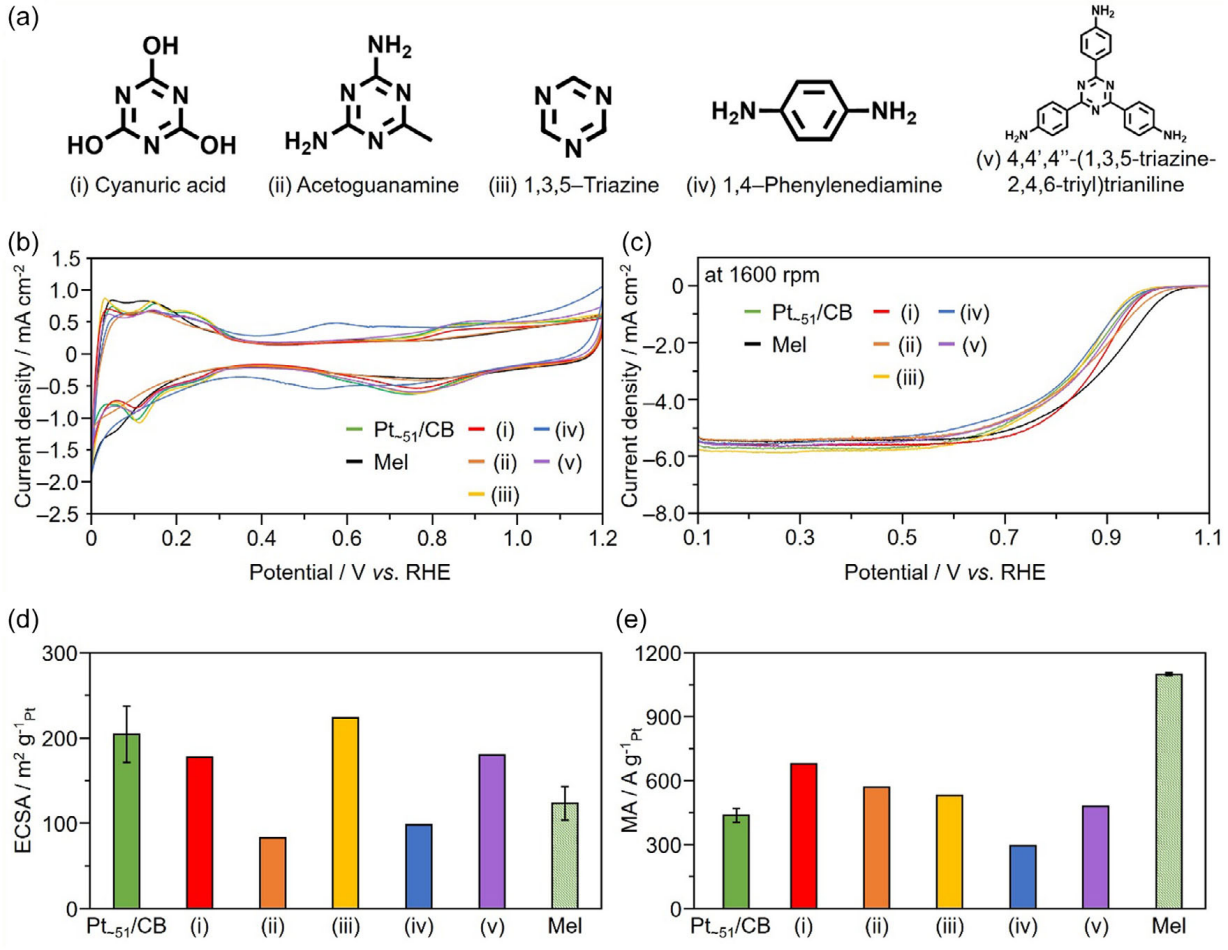
Attempts and results of ORR activity enhancement using organic compounds other than melamine. (a) Structures of the organic additives: (i) cyanuric acid, (ii) acetoguanamine, (iii) 1,3,5‐triazine, (iv) 1,4‐phenylenediamine, and (v) 4,4′, 4′′‐(1,3,5‐triazine‐2,4,6‐triyl)trianiline. Comparison of the (b) CVs and (c) LSVs of Pt_∼51_/CB with and without each additive. (d) ECSAs calculated from H^+^ adsorption obtained from the CVs and e MAs calculated from the Koutecký–Levich plots (Figure S44) obtained from the LSVs at 0.9 V versus RHE for Pt_∼51_/CB with each additive.

We further investigated the effect of organic modification on ORR activity using melamine‐like compounds. The effects of the 15 types of organic compounds [39] on the ORR activity can be categorized into four main groups (Figures S41–S45): (a) compounds that cause a decrease in the MA (<0.9 times the MA of Pt NC/CB), (b) compounds that have little effect on the MA (0.9–1.1 times the MA of Pt NC/CB), (c) compounds that improve both the MA and SA (1.1–1.5 times the MA and SA of Pt NC/CB), and (d) compounds that significantly improve the MA (>1.5 times the MA of Pt NC/CB). For a clearer discussion, the results of five representative compounds are shown in Figure 10a. The cyclic voltammetry and linear sweep voltammetry results measured using Pt_∼51_/CB modified with these compounds are shown in Figure 10b,c, respectively, and the ECSAs and MAs are shown in Figure 10d,e.

Melamine consists of a 1,3,5‐triazine ring with three nitrogen atoms and three terminal NH_2_ groups coordinated at the 2, 4, and 6 positions. Therefore, to investigate the influence of the terminal NH_2_ groups, a comparison of the ORR activity was performed when these groups were changed to different functional groups. First, Pt_∼51_/CB modified with cyanuric acid (i), in which all three terminal NH_2_ groups are replaced with less electron‐donating OH groups, was investigated. A slight decrease in the ECSA was observed, while the MA increased by approximately 1.5 times. Because the decrease in the ECSA was smaller than that observed for melamine, the electron‐donating ability of the terminal functional groups is important for the adsorption ability on Pt particles. Here, the introduction of functional groups at the 2, 4, and 6 positions is likely to induce equilibrium with tautomeric forms, which not only affects the adsorption ability to Pt particles, but also influences the electron withdrawal from the Pt particles by the 1,3,5‐triazine ring, which will be discussed later [39]. When acetoguanamine (ii), in which one of the three NH_2_ groups of melamine is replaced with the less electron‐donating CH_3_ group, was used, a significant decrease in the ECSA was observed. This indicates that acetoguanamine strongly adsorbs to Pt particles. As previously mentioned, melamine is presumed to adsorb to the Pt particles through the NH_2_ groups or the nitrogen atoms of the 1,3,5‐triazine ring. From DFT calculations, it is predicted that the 1,3,5‐triazine ring coordinates vertically to the Pt(111) surface rather than in a planar orientation [40]. Therefore, it can be inferred that not all of the nitrogen atoms in melamine adsorb to the Pt particles, and the absence of one or two NH_2_ groups does not hinder the adsorption ability to Pt particles. This is consistent with our DFT calculation results (Mel_*x*_/Pt_17_/graphite). Therefore, Pt_∼51_/CB modified with 1,3,5‐triazine (iii), which lacks terminal NH_2_ groups, was also investigated. No change in the ECSA was observed after modification with 1,3,5‐triazine, and it was revealed that 1,3,5‐triazine does not adsorb to Pt particles. From these findings, it can be inferred that the presence of at least one electron‐donating terminal functional group is necessary for adsorption to Pt particles.

Next, to investigate the influence of the 1,3,5‐triazine structure, Pt_∼51_/CB was modified with 1,4‐phenylenediamine (iv), which is composed of two terminal NH_2_ groups attached to a benzene ring. A similar decrease in the ECSA to that observed for melamine modification was observed (Figure 10d). This result supports the previous assumption that the presence of terminal NH_2_ groups plays a significant role in the adsorption to Pt particles. However, the MA decreased by approximately 35% compared with that before modification (Figure 10e). The 1,3,5‐triazine ring becomes electron‐deficient owing to the three electronegative nitrogen atoms, which results in a stronger electron‐withdrawing character compared with that of the benzene ring owing to the lack of π electrons [41]. It is inferred that shifting the charge state of the adsorbed Pt particles more toward the positive side controls the stabilization energies of the ORR intermediates. Therefore, to weaken the interaction between the Pt particles and 1,3,5‐triazine ring, Pt_∼51_/CB was modified with 4,4′, 4′′‐(1,3,5‐triazine‐2,4,6‐triyl)trianiline (v), which contains a phenyl group between the 1,3,5‐triazine ring and terminal NH_2_ groups. Modification with 4,4′, 4′′‐(1,3,5‐triazine‐2,4,6‐triyl)trianiline led to a smaller decrease in the ECSA associated with adsorption to the Pt particles, but there was no change in the MA compared with that before modification. Furthermore, when compounds with a five‐membered structure containing heteroatoms, which are π‐electron‐rich aromatics, were used, the MA instead decreased after modification (Figures S41 and S42). From these observations, it is believed that the π‐electron‐deficient aromatic 1,3,5‐triazine ring causes a slight positive shift in the electronic state of the Pt particles, and this shift contributes to the destabilization of the ORR intermediates. These results are consistent with the previously mentioned Pt L_3_‐edge XANES spectra (Figure 7a and Figure S34).

From the above results, to improve the ORR activity through organic modification, it is crucial for the organic compound to have both terminal NH_2_ groups that induce adsorption to Pt particles and the 1,3,5‐triazine structure that promotes a positive shift in the electronic state of the Pt_∼51_ particles. Therefore, we conclude that, modification with melamine is optimal for enhancing the ORR activity of Pt NC supported catalysts [30]. Furthermore, compared with Pt NP/CB, Pt NC/CB exhibits a larger electron‐withdrawing electronic state change of the Pt particles (Figure 7 and Figure S34). Such a large electronic state change in the Pt NCs likely promotes the destabilization of the ORR intermediates to a greater extent, which is why Mel/Pt NC/CB shows a more pronounced melamine modification effect than Mel/Pt NP/CB.

## 3 Conclusion

In this study, we developed highly active ORR electrocatalysts using Pt NCs with a particle size of approximately 1 nm as precursors. The aim was to improve the ORR activity and durability through modifying the Pt NCs with melamine. Additionally, the origin of the activity enhancement due to organic modification was clarified through DFT calculations and various property evaluations. The main findings were as follows:

1) We successfully prepared Pt_*x*_ NC CB‐supported catalysts (Pt_*x*_/CB) using Pt_*x*_ NCs (*x* = 17, ∼35, ∼51, and ∼ 66) as precursors and demonstrated that they show higher ORR activity than commercial Pt NP/CB. The MA of the Pt_17_ NC/CB catalyst was 3.25 times higher than that of the commercial Pt NP/CB catalyst, and it also showed excellent durability.
2) Pt_*x*_/CB had a mainly smaller Pt particle size than Pt NP/CB, resulting in a significant improvement in the MA.
3) Melamine modification of the Pt_*x*_/CB catalysts further significantly improved the ORR activity. In particular, the MA of Mel/Pt_∼51_/CB (1098 A/g_Pt_) was 2.51 times higher than that before melamine modification and approximately 6.37 times higher than that of the commercial Pt NP/CB catalyst, demonstrating a superior enhancement in the activity by melamine modification compared with the activity of the Pt NP/CB catalyst.
4) Mel/Pt_∼51_/CB showed a lower MA decrease rate (12.8%) after the ADT than Mel/Pt NP/CB (32.3%), representing 2.5 times better durability than Mel/Pt NP/CB. After the ADT, Mel/Pt_∼51_/CB had 11.0 times higher MA than Pt NP/CB (87.5 vs. 964.0 A/g_Pt_). Particularly in Mel/Pt_∼51_/CB, the exceptionally high SA associated with melamine modification persists even after ADT. Such a strong melamine modification effect is presumed to be due to the stronger adsorption ability of Pt NCs, which have a unique electronic/geometrical structure, to melamine.
5) To enhance the ORR activity through organic modification, the structure of melamine, which possesses both terminal NH_2_ groups that induce adsorption to Pt particles and the 1,3,5‐triazine structure that promotes a positive shift in the electronic state of the adsorbed Pt particles, is crucial.

These findings are expected to significantly contribute to the development of highly active and durable ORR catalysts using small Pt NCs and the widespread use of PEFCs with reduced Pt usage. Furthermore, it is expected that this study will greatly contribute to a better understanding of the mechanisms through which Pt NC‐supported catalysts and melamine modification enhance the ORR activity.

## 4 Methods

### 4.1 Preparation of the Pt NC CB‐Loaded Catalysts

Based on previous reports [9, 10, 11, 12, 13, 14, 15, 16], we synthesized Pt_∼51_(CO)_*m*_(PET)_*l*_ (CO = carbon monoxide, PET = 2‐phenylethanethiolate) (Scheme S1a). In the synthesis, chloroplatinic acid and sodium hydroxide were first uniformly dissolved in an ethylene glycol solution by ultrasonic treatment, followed by heating at 120°C in air. At this time, Pt NCs are presumed to be weakly protected by OH^**−**^ groups, and CO molecules generated by the oxidation of ethylene glycol [9]. The solution was then rapidly cooled to room temperature, and 2‐phenylethanethiol was added, followed by stirring for 1 h to perform ligand exchange, yielding Pt_*n*_(CO)_*m*_(PET)_*l*_. The obtained Pt_*n*_(CO)_*m*_(PET)_*l*_ was washed with a mixed solvent of ultrapure water and methanol. A strong peak attributed to Pt_∼51_(CO)_*m*_(PET)_*l*_ was observed at approximately *m*/*z* = 11 000 in the matrix‐assisted laser desorption/ionization mass spectrum (MALDI–MS) of the product (Figure 2a). Using the same method, Pt_∼35_(CO)_*m*_(PET)_*l*_ and Pt_∼66_(CO)_*m*_(PET)_*l*_ were synthesized by varying the thermal reduction time. In addition, by using triphenylphosphine (PPh_3_) as the ligand instead of PET, [Pt_17_(CO)_12_(PPh_3_)_8_]^*z*^ (Figure S3a) was synthesized (Scheme S1b). [Pt_17_(CO)_12_(PPh_3_)_8_]^*z*^ was confirmed to be synthesized with atomic precision and purity by electrospray ionization mass spectrometry (ESI‐MS; Figure S3b).

TEM images of Pt_∼51_(CO)_*m*_(PET)_*l*_ and [Pt_17_(CO)_12_(PPh_3_)_8_]^*z*^ are shown in Figure 2b and S3c, respectively. From Figure 2b, the average particle size of Pt_∼51_(CO)_*m*_(PET)_*l*_ was found to be 1.18 ± 0.21 nm, confirming that it was synthesized with extremely small and monodisperse sizes. These Pt NCs were used as precursors for the preparation of supported catalysts. Ketjenblack (EC300J) [42], which has high conductivity, was used as the support material. For the catalyst preparation (Figure S4), the Pt_*x*_ NCs were first dissolved in dichloromethane, and the Pt concentration of the solution was adjusted by inductively coupled plasma mass spectrometry. The Pt_*x*_ NCs dispersion was then added to the CB (Ketjenblack) support to achieve a Pt loading ratio of 20.0 wt%. The adsorption of the Pt_*x*_ NCs to CB was performed by the impregnation method, rather than the liquid‐phase adsorption method used in our previous studies [9, 43]. Here, the organic ligands covering the Pt NCs inhibit the approach of the reactive substrate and act as electrical resistance between the CB support and NCs, which leads to a significant decrease in the catalytic activity [44]. By subjecting the CB‐supported Pt_*x*_ NCs to calcination treatment at an appropriate temperature under reduced pressure [45, 46], most of the ligands were removed while preventing significant oxidation of the Pt NCs. The calcination temperature was set to 250°C based on the results of thermogravimetric analysis (Figure S5). This calcination also serves to immobilize the Pt_*x*_ NCs on the support [27].

The results of Pt particle size analysis of the TEM images at each stage of the catalyst preparation using Pt_*x*_ NCs as the precursors are shown in Figure 2b and S6–S8. For Pt_∼51_(CO)_*m*_(PET)_*l*_ on CB before calcination (Pt_∼51_(CO)_*m*_(PET)_*l*_/CB), a slight increase in the Pt particle size was observed (1.30 ± 0.27 nm; Figure 3a). Furthermore, for the calcined Pt_∼51_ NC CB‐supported catalyst (Pt_∼51_/CB) derived from Pt_∼51_(CO)_*m*_(PET)_*l*_/CB, a further increase of the size of some Pt particles was observed (1.55 ± 0.51 nm; Figure 3b). Owing to the principle of the impregnation method, some of the Pt NCs were weakly supported on the CB via physical adsorption, leading to aggregation during calcination. However, the majority of the Pt particles maintained their relatively small size. The same trend was observed for the preparation of the catalysts using Pt NCs with other sizes as precursors (Figures S6–S8).

Next, from elemental mapping (C, O, S, and Pt) by scanning TEM (STEM) coupled with energy dispersive X‐ray spectroscopy (EDS), it was observed that Pt was uniformly dispersed on the CB for Pt_∼51_/CB (Figure 4a–e). However, it was difficult to determine the removal of the ligands due to calcination solely from the STEM images. Therefore, the bonding state of Pt was confirmed by Pt L_3_‐edge FT‐EXAFS measurements (Figure 4f, and Figures S10 and S13). For the calcined Pt_∼51_/CB, compared with Pt_∼51_(CO)_*m*_(PET)_*l*_/CB before calcination, distinct peaks of Pt–Pt bonds (∼2.7 Å) [47, 48, 49] and Pt—C (O) bonds (∼1.7 Å) were observed. This suggests that, by applying the calcination treatment, immobilization of Pt_∼51_(CO)_*m*_(PET)_*l*_ on CB occurred [50]. These distinct Pt–Pt bonds are presumed to be caused by the aggregation of some of the weakly adsorbed Pt_∼51_(CO)_*m*_(PET)_*l*_ NCs, as previously mentioned. Additionally, peaks of Pt–S bonds (2.0 Å) [9] were observed for both Pt_∼51_(CO)_*m*_(PET)_*l*_ and Pt_∼51_(CO)_*m*_(PET)_*l*_/CB. However, after calcination (Pt_∼51_/CB), these peaks almost disappeared. This indicates that for Pt_∼51_/CB, the calcination treatment caused PET to detach from the Pt_∼51_ NCs, leading to the cleavage of the Pt—S bonds. Considering the thermogravimetric analysis results of Pt NCs with various sizes obtained in a previous study [9], it is believed that PET similarly detached from the Pt NCs during the calcination process for Pt_∼35_(CO)_*m*_(PET)_*l*_ and Pt_∼66_(CO)_*m*_(PET)_*l*_. A similar trend was observed for the catalyst prepared using Pt_17_(CO)_12_(PPh_3_)_8_ as the precursor (Pt_17_/CB). After calcination, the peak corresponding to the Pt–P bonds (∼2.0 Å) [51] disappeared (Figure S11a,b), suggesting that PPh_3_ detached from Pt_17_(CO)_12_(PPh_3_)_8_. Next, to clarify the charge state of the Pt particles during the catalyst preparation process, the Pt L_3_‐edge XANES spectra were compared. From the peak intensities of the white lines in the XANES spectra of Pt_∼51_(CO)_∼12_(PET)_∼33_, Pt_∼51_(CO)_*m*_(PET)_*l*_, and Pt_∼51_(CO)_*m*_(PET)_*l*_/CB (Figure 4g), they exhibited spectra close to the neutral valence. After calcination, a slight shift of the electronic state toward the positive side was observed, which was attributed to the interaction between the catalyst and CB support. However, no significant oxidation was observed, and it is believed that the Pt particles maintained their metallic electronic state [44]. A similar trend was also observed for Pt_17_/CB (Figure S11c). Furthermore, from X‐ray photoelectron spectroscopy (XPS), metallic Pt similar to that in the XANES spectrum was observed (Figure S12a). It was also revealed that S and P from the ligands were oxidized and moved onto the CB support (Figure S12b). Movement of S from Pt to CB was also observed in the STEM‐EDX analysis (Figure 4a–e).

### 4.2 Preparation of the Melamine‐Modified Pt NP/CB Catalysts or Pt NC CB‐Loaded Catalysts

First, a 0.01 M melamine aqueous solution was prepared (Scheme S3). Pt NP/CB or Pt NC CB‐loaded catalysts‐coated working electrode was then immersed in this solution for 10 min, the procedure outlined in Scheme S2 was performed, and electrochemical measurements were performed [41].

## Supporting Information

Additional supporting information can be found online in the Supporting Information section. The online version contains supplementary material available from the Wiley Online Library or from the author. Additional experimental and characterization sections are included: additional ESI‐MS, TGA, XPS, FT‐IR, XANES, EXAFS spectra, TEM images, results of DFT calculations and electrocatalytic activity. **S** **upporting** **Scheme** **S1:** Synthesis scheme of (a) Pt*_x_* (CO)*_m_* (PET)_*l*_ (*x* = ∼35, ∼51 and ∼66), and (b) Pt_17_(CO)_12_(PPh_3_)_8_ by polyol reduction method. **Supporting Scheme S2:** Preparation working electrodes of (a) Pt_*x*_/CB (*x* = 17, ∼35, ∼51 and ∼66) and (b) Pt NPs/CB. **Supporting** **Schme S3:** Preparation melamine aqueous solution (0.01 mol/L). **Supporting Fig. S1:** Schematic of a polymer electrolyte fuel cell (PEFC) and the reaction at the electrode: Reproduced with permission from Ref. 1. Copyright 2021 The Royal Society of Chemistry. **Supporting Fig. S2:** Structure and detailed information of melamine. **Supporting Fig. S3:** (a) Geometric structure, (b) ESI‐MS spectrum and (c) a TEM image and the resulting Pt size of Pt_17_(CO)_12_(PPh_3_)_8_. (b) was reproduced with permission from Ref. 2. Copyright 2023, The Royal Society of Chemistry. **Supporting Fig. S4:** Schematic of the preparation procedure: adsorption of Pt NCs onto CB, calcination of the catalyst at 250 °C for 120 minutes. **Supporting Fig. S5:** TGA date for (a) Pt_∼51_(CO)_*m*_(PET)_*l*_ and (b) [Pt_17_(CO)_12_(PPh_3_)_8_]Cl_*n*_. (a) The weight of Pt_∼51_(CO)_*m*_(PET)_*l*_ was decreased by two steps. The first decrease seems to be due to the vaporization of PET caused by the P−S dissociation, whereas the second decrease the vaporization of the remaining CO. (b) The weight of [Pt_17_(CO)_12_(PPh_3_)_8_]Cl_*n*_ was decreased by two steps. The first decrease seems to be due to the vaporization of PPh_3_ caused by the Pt−P dissociation, whereas the second decrease the vaporization of the remaining CO. **Supporting Fig. S6:** TEM images and the resulting Pt size histogram of Pt_17_ NCs after (a) adsorption on CB (Pt_17_(CO)_12_(PPh_3_)_8_/CB) and (b) calcination of Pt_17_(CO)_12_(PPh_3_)_8_/CB (Pt_17_/CB; the loading weight of Pt: 20.0 wt%). **Supporting Fig. S7:** TEM images and the resulting Pt size histogram of Pt_∼35_ NCs after (a) adsorption on CB (Pt_∼35_(CO)_*m*_(PET)_*l*_/CB) and (b) calcination of Pt_∼35_(CO)*_m_* (PET)_*l*_/CB (Pt_∼35_/CB; the loading weight of Pt: 20.0 wt%). **Supporting Fig. S8:** TEM images and the resulting Pt size histogram of Pt_∼66_ NCs after (a) adsorption on CB (Pt_∼66_(CO)_*m*_(PET)_*l*/CB_) and (b) calcination of Pt_∼66_(CO)*_m_* (PET)_*l*_/CB (Pt_∼66_/CB; the loading weight of Pt: 20.0 wt%). **Supporting Fig. S9:** TEM images and the resulting histogram of commercial Pt NPs/CB (TEC10E50E; the loading weight of Pt: 46.9 wt%). **Supporting Fig. S10:** Pt L_3_‐edge EXAFS spectra of Pt_∼51_(CO)_*m*_(PET)_*l*_, Pt_∼51_(CO)_*m*_(PET)_*l*_/CB and Pt_∼51_/CB together with Pt foil and PtO_2_ powder as reference. **Supporting Fig. S11:** Pt L_3_‐edge (a) FT‐EXAFS, (b) EXAFS and (c) XANES spectra of Pt_17_(CO)_12_(PPh_3_)_8_, Pt_17_(CO)_12_(PPh_3_)_8_/CB and Pt_17_/CB together with Pt foil and PtO_2_ powder as reference. **Supporting Fig. S12:** (a) Pt 4f and (b) S 2p XPS spectra of Pt_∼51_(CO)*_m_* (PET)*_l_*, Pt_∼51_(CO)*_m_* (PET)*_l_*/CB and Pt_∼51_/CB. (c) Pt 4f and (d) P 2p XPS spectra of Pt_17_(CO)_12_(PPh_3_)_8_, Pt_17_(CO)_12_(PPh_3_)_8_/CB and Pt_17_/CB. **Supporting Fig. S13:** Wavelet transform (WT) images of Pt L_3_‐edge EXAFS spectra for (a) Pt_∼51_/CB and (b) Mel/Pt_∼51_/CB. WT analysis of the Pt L_3_‐edge successfully resolved the overlapping adsorption species that are difficult to distinguish in the conventional radial structure function. Specifically, the intense peak at *r* ∼ 2.7 Å exhibited a maximum intensity in the higher wavenumber region (*k* > 10 Å^–1^), which is a characteristic feature of Pt–Pt bonding involving heavy atom scattering. In contrast, the intensities observed at *r* ∼1.6 Å and ∼1.9 Å were clearly identified in the lower wavenumber region; the former is attributed to Pt–C/N scattering from light atoms, while the latter, appearing at a slightly higher *k*‐range than the light atoms, is consistent with the Pt–S bond. **Supporting Fig. S14:** A schematic of the preparation method of catalyst slurry and working electrode. **Supporting Fig. S15:** Photographs of the apparatus used in this work for electrochemical experiments. The setup modified from our previous study (reference electrode) is highlighted in red color. Reproduced with permission from Ref. 2. Copyright 2023, The Royal Society of Chemistry. **Supporting Fig. S16:** CV spectra after electrochemical cleaning of (a) Pt NPs/CB, (b) Pt_∼51_/CB and c Pt_17_/CB. The loading weight of Pt were 46.9, 20.0 and 20.0 wt% for Pt NPs/CB, Pt_∼51_/CB and Pt_17_/CB, respectively. **Supporting Fig. S17:** TEM images and the resulting Pt size histograms of (a) Pt NPs/CB, (b) Pt_∼51_/CB and (c) Pt_17_/CB after CV cleaning. The loading weight of Pt were 46.9, 20.0 and 20.0 wt% for Pt NPs/CB, Pt_∼51_/CB and Pt_17_/CB, respectively. **Supporting Fig. S18:** The obtained Koutecky−Levich plots and mass activities (MAs) of Pt NPs/CB(46.9 wt% Pt) and Pt NPs/CB(20.0 wt% Pt). The loading weight of Pt were 46.9 and 20.0 wt% for Pt NPs/CB(46.9 wt% Pt) and Pt NPs/CB(20.0 wt% Pt), respectively. The Pt loadings for Pt NPs/CB(46.9 wt% Pt) and Pt NPs/CB(20.0 wt% Pt) on the RDE were set to 17.8 and 5.05 μg/cm^2^, respectively. Evaluations using Koutecky–Levich plots theoretically assume a smooth, planar electrode surface. In our case, the ORR activity of Pt/CB with a thicker catalyst layer (i.e., those with lower Pt weight content; 20.0 wt% Pt) may be underestimated. **Supporting Fig. S19:** The obtained Koutecky−Levich plots of Pt NPs/CB, Pt_∼51_/CB and Pt_17_/CB. The loading weight of Pt were 46.9, 20.0 and 20.0 wt% for Pt NPs/CB, Pt_∼51_/CB and Pt_17_/CB, respectively. To ensure a uniform thickness of the CB layer, the Pt loadings for Pt NPs/CB and Pt_*x*_/CB on the RDE were set to 17.8 and 5.05 μg/cm^2^, respectively. **Supporting Fig. S20:** (a) Optimized structure for Pt_17_/graphite(X) (X = A, B, C or D) and (b) free‐energy diagram for ORR through a direct four‐electron pathway on Pt_17_/graphite(*X*) (*X* = A, B, C or D) or Pt(111) under the potential of 0.9 V vs. SHE. In (b), Pt_17_/graphite(X) and Pt(111) are abbreviated as Catalyst. Reproduced with permission from Ref. 2. Copyright 2023, The Royal Society of Chemistry. **Supporting Fig. S21:** The protocol for the ADT of electrochemical measurements. **Supporting Fig. S22:** CVs before and after ADT of (a) Pt NPs/CB, (b) Pt_∼51_/CB and c Pt_17_/CB. The loading weight of Pt were 46.9, 20.0 and 20.0 wt% for Pt NPs/CB, Pt_∼51_/CB and Pt_17_/CB, respectively. **Supporting Fig. S23:** LSVs before and after ADT of (a) Pt NPs/CB, (b) Pt_∼51_/CB and (c) Pt_17_/CB. The loading weight of Pt were 46.9, 20.0 and 20.0 wt% for Pt NPs/CB, Pt_∼51_/CB and Pt_17_/CB, respectively. **Supporting Fig. S24:** The obtained Koutecky−Levich plots of (a) Pt NPs/CB, (b) Pt_∼51_/CB and (c) Pt_17_/CB before and after ADT. The loading weight of Pt were 46.9, 20.0 and 20.0 wt% for Pt NPs/CB, Pt_∼51_/CB and Pt_17_/CB, respectively. To ensure a uniform thickness of the CB layer, the Pt loadings for Pt NPs/CB and Pt_*x*_/CB on the RDE were set to 17.8 and 5.05 μg/cm^2^, respectively. **Supporting Fig. S25:** TEM images and the resulting Pt size histograms of (a) Pt NPs/CB, (b) Pt_∼51_/CB and (c) Pt_17_/CB after ADT. **Supporting Fig. S26:** FT‐IR spectra before and after melamine modification for (a) Pt_∼51_/CB and (b) Pt NPs/CB. In (a), CO bond is attributed from the protective ligands of Pt_∼51_ NCs in the synthesis process. **Supporting Fig. S27:** Comparison of CVs before and after melamine modification for (a) Pt NPs/CB and (b) Pt_17_/CB. The loading weight of Pt were 46.9 and 20.0 wt% for Pt NPs/CB and Pt_17_/CB, respectively. **Supporting Fig. S28:** Comparison of LSVs before and after melamine modification for (a) Pt NPs/CB and (b) Pt_17_/CB. The loading weight of Pt were 46.9 and 20.0 wt% for Pt NPs/CB and Pt_17_/CB, respectively. **Supporting Fig. S29:** Comparison of LSVs before and after melamine modification for (a) Pt NPs/CB and (b) Pt_17_/CB. The loading weight of Pt were 46.9 and 20.0 wt% for Pt NPs/CB and Pt_17_/CB, respectively. **Supporting Fig. S30:** Comparison of LSVs before and after ADT for (a) Mel/Pt NPs/CB, (b) Mel/Pt_∼51_/CB and (c) Mel/Pt_17_/CB. The loading weight of Pt were 46.9, 20.0 and 20.0 wt% for Mel/Pt NPs/CB, Mel/Pt_∼51_/CB and Mel/Pt_17_/CB, respectively. **Supporting Fig. S31:** Koutecky−Levich plots before and after ADT (modification melamine) for (a) Mel/Pt NPs/CB, (b) Mel/Pt_∼51_/CB and (c) Mel/Pt_17_/CB. The loading weight of Pt were 46.9, 20.0 and 20.0 wt% for Mel/Pt NPs/CB, Mel/Pt_∼51_/CB and Mel/Pt_17_/CB, respectively. To ensure a uniform thickness of the CB layer, the Pt loadings for Pt NPs/CB and Pt_*x*_/CB on the RDE were set to 17.8 and 5.05 μg/cm^2^, respectively. **Supporting Fig. S32:** Comparison of CVs before and after ADT for (a) Mel/Pt NPs/CB, (b) Mel/Pt_∼51_/CB and (c) Mel/Pt_17_/CB. The loading weight of Pt were 46.9, 20.0 and 20.0 wt% for Mel/Pt NPs/CB, Mel/Pt_∼51_/CB and Mel/Pt_17_/CB, respectively. **Supporting Fig. S33:** SEM images and energy‐dispersive X‐ray spectroscopy maps of Pt‐M, C‐K, O‐K, S‐K, and N‐K of Mel/Pt_∼51_/CB (a) before and (b) after ADT. **Supporting Fig. S34:** Pt L_3_‐edge XANES spectra before and after melamine modification for (a) Pt NPs/CB and (b) Pt_17_/CB together with Pt foil and PtO_2_ powder as reference. These changes in the electronic states were observed under conditions of forced excess adsorption of melamine. **Supporting Fig. S35:** TEM images and the resulting Pt size histograms of (a) Mel/Pt NPs/CB, (b) Mel/Pt_∼51_/CB and (c) Mel/Pt_17_/CB after ADT. **Supporting Fig. S36:** Comparison of electrochemical measurements in sulfuric acid or perchloric acid. Comparison of the (a, c) CVs and (b, d) LSVs for (a, b) Pt_∼51_/CB and (c, d) Mel/Pt_∼51_/CB in 0.1 M HClO_4_ or H_2_SO_4_ aq. Resulting (e) ECSAs using proton adsorption current from CVs and (f) MAs calculated using the Koutecký–Levich plots from LSVs at 0.9 V vs. RHE. **Supporting Fig. S37:** Results of DFT calculations. (a) Optimized structure of Pt_17_/graphite. In this figure, the atom index (i) of each Pt atom and the site (A−D) for the reaction with O_2_ are also described. (b) Optimized intermediate structure for O_2_/Pt_17_/graphite(A). (c) Optimized intermediate structure for (O + OH)/Pt_17_/graphite(A). The other intermediate structures, O_2_/Pt_17_/graphite(*X*) and (O + OH)/Pt_17_/graphite(*X*), obtained by starting Pt_17_/graphite(*X*) (*X* = B, C, or D). (d) Electric charge of each Pt atom for Pt_17_/graphite. Reproduced with permission from Ref. 2. Copyright 2023, The Royal Society of Chemistry. **Supporting Fig. S38:** Results of DFT calculations. (A) (a−d) Optimized several Mel1(*X*)/Pt_17_/graphite (*X* = A−D) structure, where I, II and III correspond to the respective reaction steps in Figure 9(d). (B) Electric charge of each Pt atom for Pt_17_/graphite and Mel1(I)/Pt_17_/graphite. **Supporting Fig. S39:** Results of DFT calculations. (a), Optimized Mel_2_/Pt_17_/graphite and (b) Mel_3_/Pt_17_/graphite structure, where I, II and III correspond to the respective reaction steps in Figure 9(d). **Supporting Fig. S40:** Free‐energy diagram for ORR through a direct four‐electron pathway on Mel_*x*_(*X*)/Pt_17_/graphite (*x* = 1–3) under the potential of 0.9 V vs. SHE, where I, II and III correspond to [2(H^+^ + e^–^) + O_2_(gas) + Mel*_x_* (*X*)/Pt_17_/graphite], [2(H^+^ + e^–^)O_2_/Mel*_x_* (*X*)/Pt_17_/graphite] and [3/2(H^+^ + e^–^)(O+OH)/Mel*_x_* (*X*)/Pt_17_/graphite]. This diagram is derived from the optimized structure in Figures S33 and 34. **Supporting Fig. S41:** Organic chemicals for the modification of Pt_∼51_/CB. Organic chemicals with (a) reduced MA, (b) maintained MA, (c) improved MA and (d) significantly improved MA. **Supporting Fig. S42:** Results of electrochemical measurements of the organic chemicals modified Pt_∼51_/CB. Comparison of (a) ECSAs calculated from CVs, (b) MAs calculated from LSVs and c SA calculated from ECSA and MA. (i)—(xv) correspond to Figure. S36. The loading weight of Pt was 20.0 wt%. **Supporting Fig. S43:** Comparison of SA calculated from ECSA and MA for (i) cyanuric acid, (ii) acetoguanamine, (iii) 1,3,5–triazine, (iv) 1,4–phenylenediamine, (v) 4,4’,4’’‐(1,3,5‐triazine‐2,4,6‐triyl)trianiline and (Mel) melamine modified Pt_∼51_/CB. The loading weight of Pt was 20.0 wt%. **Supporting Fig. S44:** Comparison of Koutecky−Levich plots for (i) cyanuric acid, (ii) acetoguanamine, (iii) 1,3,5 triazine, (iv) 1,4–phenylenediamine, (v) 4,4’,4’’‐(1,3,5‐triazine‐2,4,6‐triyl)trianiline and (Mel) melamine modified Pt_∼51_/CB. The loading weight of Pt was 20.0 wt%. **Supporting Fig. S45:** The structures of 1,3,5‐triazine derivatives with low water solubility, (a) thiocyanuric acid and (b) cyanuric chloride. **Supporting Table S1:** The number of transferred electrons for each catalyst at diffusion‐limited region.

## Author Contributions

**Ryuki Kurosaki:** investigation (lead). **Tokuhisa Kawawaki:** conceptualization (equal), funding acquisition (equal), supervision (equal), writing – original draft (lead), review and editing (equal). **Kaoru Ikeda:** investigation (supporting). Kazutaka Oiwa: investigation (supporting). **Kotaro Sato:** investigation (supporting). **Haruna Tachibana:** investigation (supporting). **Minoru Inaba:** writing – review and editing (supporting). **Kenji Iida:** investigation (equal), writing – review and editing (supporting). **Yuichi Negishi** **:** conceptualization (lead), funding acquisition (lead), supervision (lead), writing – review and editing (lead).

## Funding

This work was supported by New Energy and Industrial Technology Development Organization (NEDO), Japan Society for the Promotion of Science (JSPS) through KAKENHI grants (Grants 23H00289, 22K19012, and 24K01459), Joint Usage/Research Center for Catalysis (Grant 22AY0056, 23AY0189), Takahashi Industrial and Economic Research Foundation, Yazaki Memorial Foundation for Science and Technology, Kumagai Foundation for Science and Technology, Iwatani Naoji Foundation, Ichimura Foundation of New Technology, Suzuki Foundation, and Japan Keirin Autorace Foundation.

## Conflicts of Interest

The authors declare no conflicts of interest.

## Supporting information

Supplementary Material

## Data Availability

The data that support the findings of this study are available from the corresponding author upon reasonable request
