# Supplementary material for: Drastic Enhancement of Activity and Durability for Oxygen Reduction Reaction by Melamine Modification for Platinum Nanocluster‐Loaded Electrocatalysts
Source: Small Sci. 2026 Mar 15;6(3):e202500632. doi: 10.1002/smsc.202500632 (PMC13098022; doi:10.1002/smsc.202500632)
Supplement: Supplementary file 1 — Supplementary Material [file SMSC-6-e202500632-s001.pdf]

Supporting Information

Drastic enhancement of oxygen reduction reaction activity and durability by melamine modification for Pt nanocluster-loaded electrocatalysts

Ryuki Kurosaki,[1] Tokuhisa Kawawaki,[1,2,*] Kaoru Ikeda,[1,2] Kazutaka Oiwa,[1] Kotaro Sato,[1,2] Haruna Tachibana,[1] Minoru Inaba,[3] Kenji Iida,[4,*] Yuichi Negishi[1,2,*]

[1]Carbon Value Research Center, Research Institute for Science and Technology, Tokyo University of Science, Kagurazaka, Shinjuku-ku, Tokyo 162−8601, Japan
[2]Institute of Multidisciplinary Research for Advanced Materials, Tohoku University, Katahira 2-1-1, Aoba−ku, Sendai 980−8577, Japan
[3]Department of Molecular Chemistry and Biochemistry, Doshisha University, 1-3 Tatara-Miyakodani, Kytotanabe, Kyoto 610−0321, Japan
[4]Institute for Catalysis, Hokkaido University, Sapporo, Hokkaido 001−0021, Japan

Corresponding Author E-mail: tokuhisa.kawawaki.d8@tohoku.ac.jp (T. Kawawaki); k-iida@cat.hokudai.ac.jp (K. Iida); yuichi.negishi.a8@tohoku.ac.jp (Y. Negishi)

S1. Chemicals

All chemicals were commercially obtained and used without further purification. $H_2PtCl_6·6H_2O$ and Commercial Pt nanoparticles/carbon black (Pt NPs/CB; TEC10E50E; 46.9 wt% Pt) were purchased from Tanaka Kikinzoku (TKK, Tokyo, Japan). Sodium hydroxide (NaOH), Triphenyl Phosphine (PPh_3), Ultrapure water (18.2 Ω), Nafion^{TM}, 2-propanol, Pt standard solution (1000 mg/L), Bismuth (Bi) standard solution (1000 mg/L), Imidazole and 2,2'-Azobis(2-methylpropionamidine) Dihydrochloride were obtained from FUJIFILM Wako Pure Chemical Corporation (Osaka, Japan). Ethylene glycol, Methanol, Dichloromethane, Acetone, Perchloric acid, Nitric acid and Hydrochloric acid were sourced from Kanto Chemical Co., Inc. (Tokyo, Japan). Melamine monomer, Trans-2-[3-(4-tert-butylphenyl)-2-methyl-2-propenylidene]malononitrile (DCTB), 1,3,5-Triazine, 1,4-Phenylendiamine, 2,4-Diamino-6-methyl-1,3,5-triazine (Acetoguanamine), Benzimidazple, N,N'-Bis(2-pyridylmethyl)-1,2-ethylenediamine, 1,1,3,3-Tetramethylguanidine, 9*H*-Purine-2,6-diamine, 3,4-Diaminofurazan, Cyromazine, Cyanuric chloride and Thiocyanuric acid were provided from Tokyo Chemical Industry Co. Ltd. (TCI, Tokyo, Japan). 2-Phenylethanethiol (PET) and Cyanuric acid were from Sigma-Aldrich Co., LLC. (Germany). 2,4,6-Trimethyl-1,3,5-triazine was from AmBeed (China). 4,4',4''-(1,3,5-Triazine-2,4,6-triyl)trianiline was from Extension (China). Ketjen black (EC300J) was from Lion Specialty Chemicals Co., Ltd. (Tokyo, Japan). Alumina paste (ALUMINA POLISHINNG SUSPENSION) was from Maruto Instrument Co., Ltd. (Tokyo, Japan).

S2. Experimental section

S2.1. Synthesis of $Pt_x(CO)_m(PET)_l$ (x = ~35, ~51 and ~66)

$Pt_x(CO)_m(PET)_l$ (x = ~35, ~51 and ~66) was synthesized using the method reported in our previous report.[1] First, NaOH (135 mg, 3.4 mmol) was dissolved in ethylene glycol (15 mL). To control the pH of the solution, NaOH was used and thereby suppress the particle size obtained by polyol reduction. Subsequently, $H_2PtCl_6·6H_2O$ (1000 μL, 0.20 mmol) was added to the ethylene glycol solution. The mixture underwent vigorous stirring for 15 minutes at 80 °C, followed by continuous stirring at 120 °C in 4, 30 and 120 minutes for $Pt_x(CO)_m(PET)_l$ (x = ~35, ~51 and ~66), respectively. After cooling to room temperature, using an ice bath, toluene (10 mL) containing PET (268 μL, 20 mmol) was promptly added. After 60 min, a mixture of water (~10 mL) and toluene (~7 mL) was added to the reaction solution. The organic phase was then separated from the water phase and dried using a rotary evaporator. The dried

product underwent washing with water followed by methanol, repeated ten times to eliminate ethylene glycol and excess PET. Finally, the product was dried again, and the target $Pt_x(CO)_m(PET)_l$ (x = ~35, ~51 and ~66) compound was extracted from the dried mixture using dichloromethane (Scheme. S1a).

S2.2. Synthesis of $[Pt_{17}(CO)_{12}(PPh_3)_8]Cl_n$ (n = 1, 2)

$[Pt_{17}(CO)_{12}(PPh_3)_8]Cl_n$ was synthesized using the method reported in our previous report.[2,3] NaOH (90 mg, 2.3 mmol) was dissolved in ethylene glycol (25 mL). To control the pH of the solution, NaOH was used and thereby suppress the particle size obtained by polyol reduction. Then, the mixture was heated and stirred at 120 °C for 8 min and 45 s to reduce Pt ions to Pt(0) and produce CO catalyzed by Pt ions. After cooling to room temperature, using an ice bath, acetone (10 mL) containing PPh_3 (1.26 g, 4.8 mmol) was promptly added. After 5 min, a mixture of water (~10 mL) and toluene (~7 mL) was added to the reaction solution. The resulting products including $[Pt_{17}(CO)_{12}(PPh_3)_8]Cl_n$ were transferred to the organic phase. Then, the organic phase was separated from the water phase and dried with a rotary evaporator. The dried product was washed with water and then methanol to eliminate ethylene glycol and excess PPh_3. At this stage, the product was a mixture of clusters of several sizes and was thus washed with acetonitrile and toluene. The product was dried and the target $[Pt_{17}(CO)_{12}(PPh_3)_8]Cl_n$ was extracted from the dried mixture with dichloromethane (Scheme. S1b).

S2.3. Preparation of Pt_x/CB catalysts (x = 17, ~35, ~51 and ~66)

Pt_x NCs (x = 17, ~35, ~51 and ~66) were dissolved in dichloromethane. CB were impregnated with the dichloromethane containing Pt_x NCs using a mortar and pestle, allowing the solvent to evaporate from the catalyst mixture. Subsequently, a catalyst with a high loading of 20 wt% was prepared by removing the ligand through a calcination process at 250 °C. The amount of Pt supported on CB was calculated from the Pt concentration in the NCs dispersion solution using inductively coupled plasma (ICP)-mass spectrometry (MS).[4,5]

S2.4. Characterization

Matrix-assisted laser desorption/ionization (MALDI)-MS

MALDI-MS spectra were recorded on a JMS-S3000 spiral time-of-flight mass spectrometer (JEOL, Tokyo, Japan) equipped with a semiconductor laser (λ = 349 nm). DCTB was used as the MALDI matrix. To minimize nanocluster (NC) dissociation induced by laser irradiation, the NC-to-matrix ratio was fixed at 1:1000.

Electrospray Ionization (ESI)-MS

ESI-MS was performed on a compact Q-TOF (time-of-flight) mass spectrometer (Bruker, Boston, MA, USA). For the measurements, a NC solution with a concentration of ~10 μg/mL in dichloromethane was electrosprayed at a flow rate of 180 μL/h.

Inductively Coupled Plasma (ICP)-MS

ICP-MS was performed on an Agilent 7850c spectrometer (Agilent Technologies, Tokyo, Japan). Bi was used as the internal standard. Pt standard solution was used to obtain the calibration line. The ICP-MS measurements were performed on the solution before and after mixing Pt_x NCs with CB to estimate the adsorbed or loaded Pt content.

Thermogravimetric analysis (TGA)

TGA was conducted on a TGA2000SA (Bruker, Boston, MA, USA) and at a heating rate of 5 °C/min (from room temperature to 900 °C) under N_2 atmosphere.

Transmission Electron Microscope (TEM)

TEM images were recorded on a H-9500 electron microscope (HITACHI, Tokyo, Japan) or JEM-2100 electron microscope (JEOL, Tokyo, Japan) operating at 200 kV, and typically using a magnification of 600,000. A NP-C15 (Okenshoji, Tokyo, Japan) was used as a copper microgrid.

Scanning Transmission Electron Microscope (STEM)-Energy Dispresive X-ray spectroscopy (EDS)

STEM images and elemental mapping were recorded with a JEM-2100F electron microscope (JEOL, Tokyo, Japan) operating at 200 kV, typically using a magnification of 600,000. The atomic resolution STEM images were obtained with an ultrahigh-resolution transmission electron microscope (FEI Titan Cubed 60-300, JEM ARM-200F) operating at 80 kV, with a Cs corrector.

X-ray Absorption Fine Structure (XAFS)

Pt L_3-edge XAFS measurements were performed at the beamline BL01B1 of the SPring-8 facility of the Japan

Synchrotron Radiation Research Institute (proposal numbers 2023B1825, 2024A1698 and 2024B1592). The incident X-ray beam was monochromatized by a Si(111) double-crystal monochromator. As references, XAFS spectra of all samples were recorded in transmission mode using ionization chambers. The X-ray energies for the Pt L_3-edges were calibrated using Pt foil. The X-ray Absorption Near Edge Structure (XANES) and Extended X-ray Absorption Fine Structure (EXAFS) spectra were analyzed using xTunes as follows. The χ spectra were extracted by subtracting the atomic absorption background using cubic spline interpolation and normalized to the edge height. The normalized data were used as the XANES spectra. The k^3-weighted χ spectra in the k range of 3.0–14.0 Å^{-1} for the Pt L_3-edge were Fourier-transformed into r space for structural analysis.

X-ray photoelectron spectroscopy (XPS)

XPS spectra were captured using the JPS-9010MC electron spectrometer (JEOL, Tokyo, Japan) under a base pressure of approximately 2×10^{-8} Torr. Excitation was achieved using the Mg-Kα line (1253.6 eV) for X-rays. Each sample was applied onto an indium (In) plate during operation, and the spectra were calibrated using the peak energies of In 3d5/2.

S2.5. Preparation of catalyst slurry

To conduct the electrochemical measurements on Pt NPs/CB and Pt_x/CB (x = 17, ~35, ~51 and ~66), a catalyst slurry was prepared with the loading weight of Pt of Pt NPs/CB and Pt_x/CB for 46.9 and 20.0 wt%, respectively. First, the catalyst powder (12.5 mg of Pt_x/CB or 18.8 mg of Pt NPs/CB) was dispersed in a solution containing Ultrapure water (19.1 mL) and 2-propanol (6.0 mL). Then, Nafion™ solution (100 µL) was added to this solution. The vial containing this mixture was sealed and ultrasonicated for 60 min in an ice bath (Figure S14).

S2.6. Electrochemical measurements

All electrochemical measurements were performed using a CHI710D electrochemical workstation (ALS, Osaka, Japan) with a RRDE-3A rotating ring disk electrode apparatus (BAS, Tokyo, Japan). A rotating disk electrode (RDE, diameter = 5 mm) was polished with an alumina paste and then sonicated in water before usage. A Pt ring electrode was used as the counter electrode. A standard hydrogen electrode was used as the reference electrode (Figure S15) In the experiment, first, the catalyst slurry was sonicated in an ice bath for 60 min. Then, the catalyst slurry (10 µL) was carefully dropped onto the RDE by the drop-cast method. To ensure a uniform thickness of the CB layer, the Pt loadings for Pt NPs/CB and Pt_x/CB on the RDE were set to 17.8 and 5.05 µg/cm^2, respectively. The electrode was then dried by rotating it at 700 rpm. After the catalyst slurry was sufficiently dried, each electrode was set in an electrochemical measurement system containing $HClO_4$ solution (0.10 mol/L, pH = 1.0) as electrolyte. The electrodes were then cleaned by bubbling N_2 gas for 30 min, followed by cyclic voltammetry (CV) 50 times in the region of 0.00–1.20 V (*vs.* RHE) at a scanning rate of 500 mV/s. Subsequently, CV was performed 3 cycles at a rate of 50 mV/s in the region of 0.00–1.20 V (*vs.* RHE) to evaluate ECSA. After CV, LSV was performed at a rate of 10 mV/s in the region of 0.10–1.10 V (*vs.* RHE) under N_2 atmosphere. The LSV measurements were also performed at a rate of 10 mV/s in the range of 0.10–1.10 V (*vs.* RHE) under an O_2 atmosphere while the RDE was rotated at 400, 900, 1600, and 2500 rpm.[6] Evaluations using Koutecky–Levich plots theoretically assume a smooth, planar electrode surface. In our case, the ORR activity of Pt_x/CB with a thicker catalyst layer (i.e., those with lower Pt weight content) may be underestimated. This is likely due to additional mass-transport resistance within the porous structure of the thicker film, which deviates from the ideal diffusion behavior assumed in the Koutecky−Levich model (Figure S19).

S2.7. Accelerated durability test (ADT)

First, after performing the electrochemical measurements according to the procedure in S2.6, the electrolyte was again bubbled with N_2 gas for 30 minutes. Then, the ADT was started at 80 °C by setting the initial potential to 0.45 V, low potential to 0.6 V and high potential to 1.0 V (*vs.* RHE) using a scan rate of 500 mV/s. Static time to 30 min and pulse width to 3 s (Figure. S21).[7] After 500 cycles (1 cycle/6 s), CV was measured and ECSA was calculated, and finally the ADT was performed for 10500 cycles. After the end of the ADT, the electrolyte was changed to a new one, and after 30 minutes of O_2 gas bubbling, LSV was measured, from which ORR mass activity was calculated, and the activity values before and after durability were compared.

S3. Calculation

S3.1. Electrochemical surface area (ECSA)

ECSA[8] was measured according to the following equation (1)

$$ECSA = \frac{Q_H}{210 \times Pt\ mass\ loading\ on\ working\ electrode} \quad \dots(1)$$

The Q_H represents the charge of adsorption of proton, determined from CV curves within the range of 0.00 to1.20

V *vs.* the RHE electrode, indicating proton adsorption on the Pt catalyst. The amount of Pt on the working electrode was determined by the volume of electrocatalyst solution applied to the rotating disk electrode surface.

S3.2. Oxygen reduction reaction (ORR) mass activity (MA)

$$\frac{1}{J} = \frac{1}{J_k} + \frac{1}{J_d} = \frac{1}{J_k} + \frac{1}{0.62nFAC_{O_2}D_{O_2}^{\frac{2}{3}}v^{-\frac{1}{6}}\omega^{\frac{1}{2}}} \quad \dots(2)$$

MA of sample was calculated according to the Koutecky–Levich equation (2).[9,10] In the equation, J_k is kinetic current, J_d is diffusion limiting current, n is the number of transfer electrons, F is Faraday constant, A is a geometric area of the electrode, C_{O2} is the concentration of O_2 in the electrolyte (1.26×10^{-6} mol/cm^3), D_{O2} is diffusion coefficient of O_2 (1.93×10^{-5} cm^2/s), v is the viscosity of the electrolyte (1.0×10^{-2} cm^2/s), and ω is the angular rotation speed of the electrode. Mass activity was obtained from J_k by dividing the measured current by the geometric area of the electrode (A) and the loading weight of Pt.[1]

S4. Density functional theory (DFT) calculation

Spin-polarized DFT calculations were performed using the Quantum Espresso program package with the Perdew–Burke–Ernzerhof functional and the empirical dispersion correction (PBE-D3).[11-13] In the calculations, the cutoff energy for the plane-wave basis was 50 Ry and a 2 × 2 × 1 k-point mesh was used. The free energy change (ΔG) was given by $\Delta G = \Delta E + \Delta E_{ZPE} - T\Delta S$ based on the theoretical model proposed by Nørskov et al.[2,14] Here, ΔE is the total energy change obtained from the DFT calculation, ΔE_{ZPE} is the change in zero-point energy, T is temperature (298.15 K), and ΔS is the change in entropy. The effect of a bias was added by shifting the free energy by $-eU$, where e is the elementary charge and U is the electrode potential. For OOH* and O_2* species, we utilized the sum of O* + OH*[15] and O* + O*, respectively. The energy of the free O_2 molecule was estimated from the experimental value of the reaction energy of $O_2 + H^+ + e^- \rightarrow H_2O$ because the high-spin ground state of O_2 cannot be well reproduced using the conventional DFT method.[14] We consider that the present correction is enough to describe the free energy diagram because the amount of the correction is different by only less than 0.15 eV compared to that for the O_2 reduction on the Pt_{13}/graphene system. [2,16]

S5. Additional Tables

Table S1 The number of transferred electrons for each catalyst at diffusion-limited region

Sample	Pt NPs/CB		Pt_{17}/CB		$Pt_{\sim51}$/CB	
	without melamine	with melamine	without melamine	with melamine	without melamine	with melamine
The number of electrons	3.86	3.89	3.76	3.86	3.83	4.22

S6. Additional Schemes

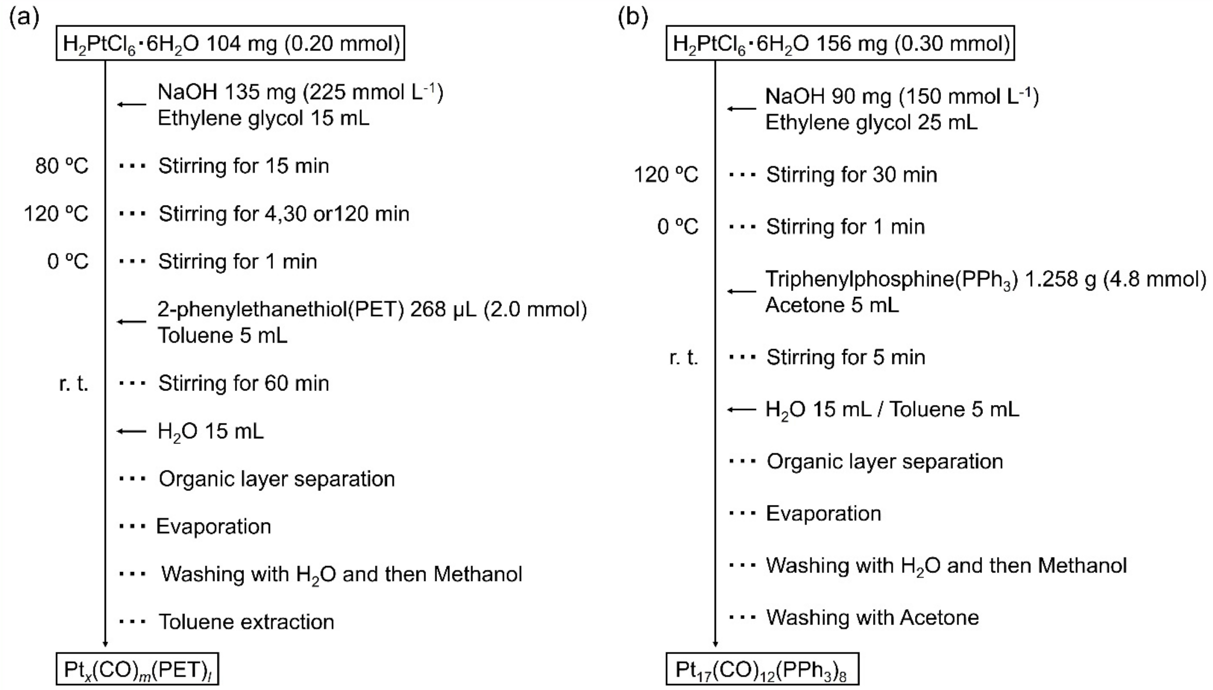

Scheme S1. Synthesis scheme of (a) $Pt_x(CO)_m(PET)_l$ (x = ~35, ~51 and ~66), and (b) $Pt_{17}(CO)_{12}(PPh_3)_8$ by polyol reduction method.

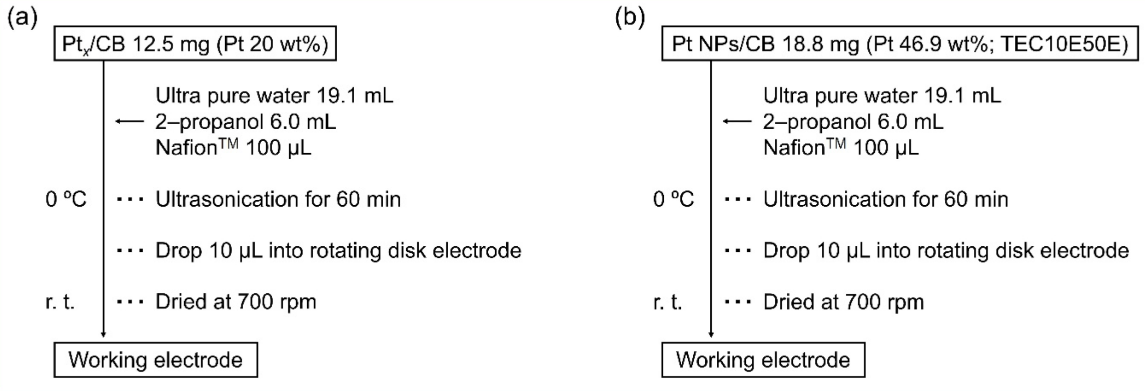

Scheme S2. Preparation working electrodes of (a) Pt_x/CB (x = 17, ~35, ~51 and ~66) and (b) Pt NPs/CB.

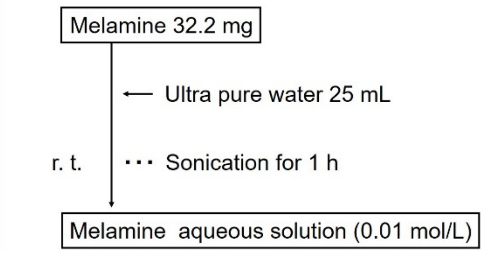

Scheme S3. Preparation melamine aqueous solution (0.01 mol/L)

S7. Additional Figures

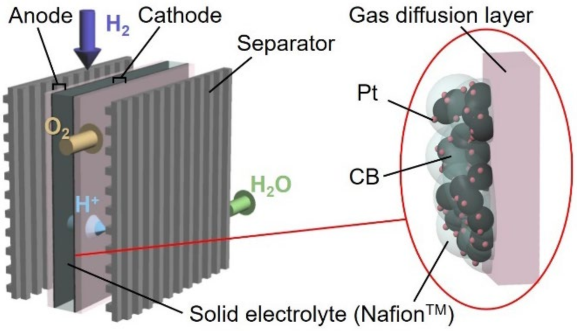

Figure S1. Schematic of a polymer electrolyte fuel cell (PEFC) and the reaction at the electrode: Reproduced with permission from Ref. 1. Copyright 2021 The Royal Society of Chemistry.

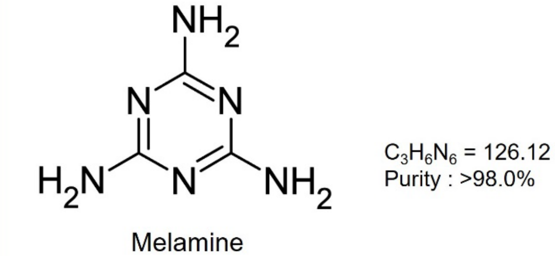

Figure S2. Structure and detailed information of melamine

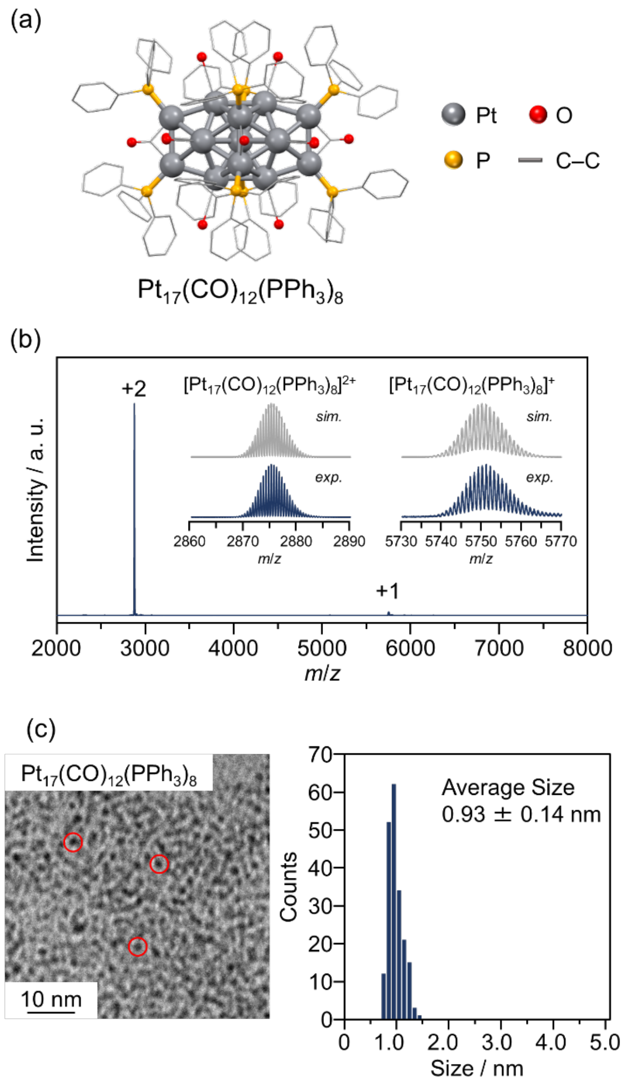

Figure S3. (a) Geometric structure, (b) ESI-MS spectrum and (c) a TEM image and the resulting Pt size of $Pt_{17}(CO)_{12}(PPh_3)_8$. (b) was reproduced with permission from Ref. 2. Copyright 2023, The Royal Society of Chemistry.

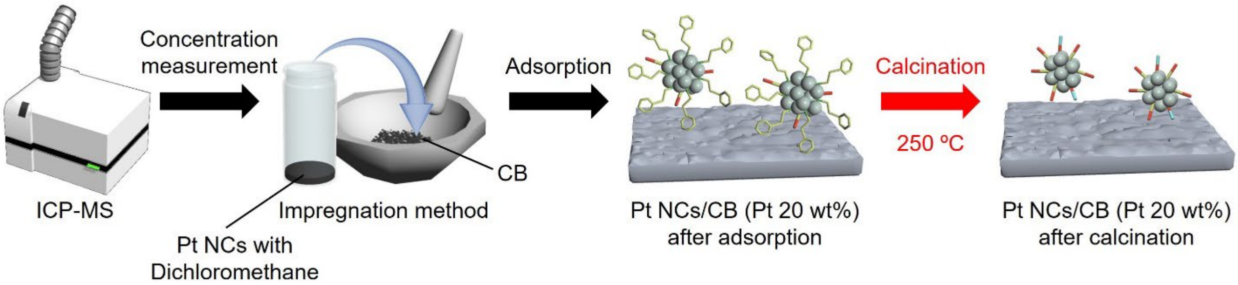

Figure S4. Schematic of the preparation procedure: adsorption of Pt NCs onto CB, calcination of the catalyst at 250 °C for 120 minutes.

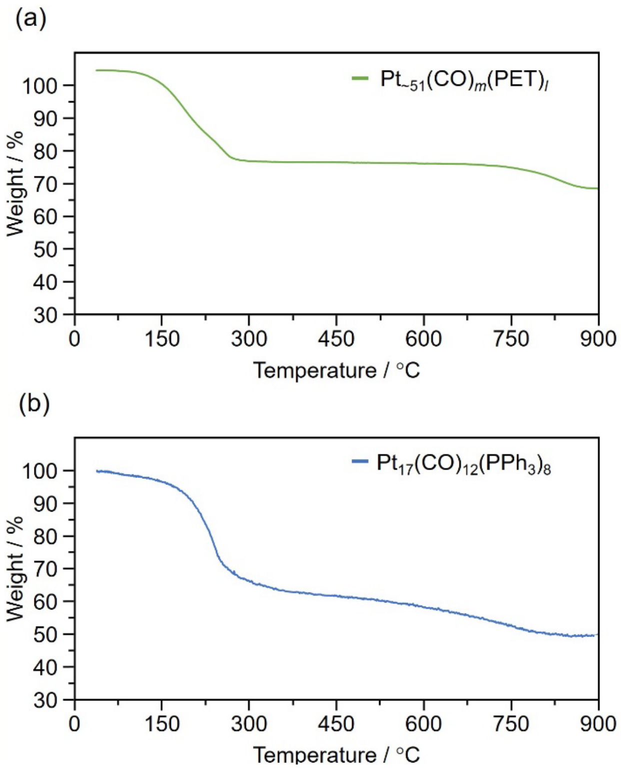

Figure S5. TGA date for (a) $Pt_{\sim51}(CO)_m(PET)_l$ and (b) $[Pt_{17}(CO)_{12}(PPh_3)_8]Cl_n$. (a) The weight of $Pt_{\sim51}(CO)_m(PET)_l$ was decreased by two steps. The first decrease seems to be due to the vaporization of PET caused by the P−S dissociation, whereas the second decrease the vaporization of the remaining CO. (b) The weight of $[Pt_{17}(CO)_{12}(PPh_3)_8]Cl_n$ was decreased by two steps. The first decrease seems to be due to the vaporization of PPh_3 caused by the Pt−P dissociation, whereas the second decrease the vaporization of the remaining CO.

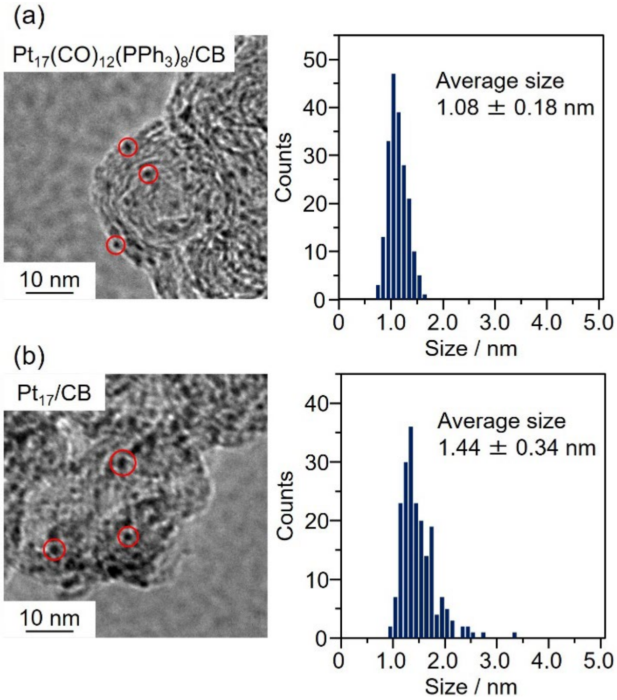

Figure S6. TEM images and the resulting Pt size histogram of Pt_{17} NCs after (a) adsorption on CB ($Pt_{17}(CO)_{12}(PPh_3)_8$/CB) and (b) calcination of $Pt_{17}(CO)_{12}(PPh_3)_8$/CB ($Pt_{17}$/CB; the loading weight of Pt: 20.0 wt%).

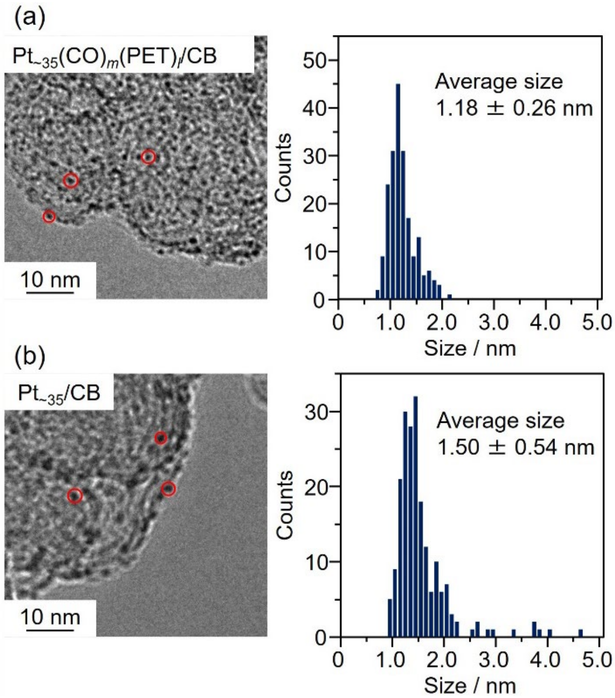

Figure S7. TEM images and the resulting Pt size histogram of $Pt_{\sim 35}$ NCs after (a) adsorption on CB ($Pt_{\sim 35}(CO)_m(PET)_l$/CB) and (b) calcination of $Pt_{\sim 35}(CO)_m(PET)_l$/CB ($Pt_{\sim 35}$/CB; the loading weight of Pt: 20.0 wt%).

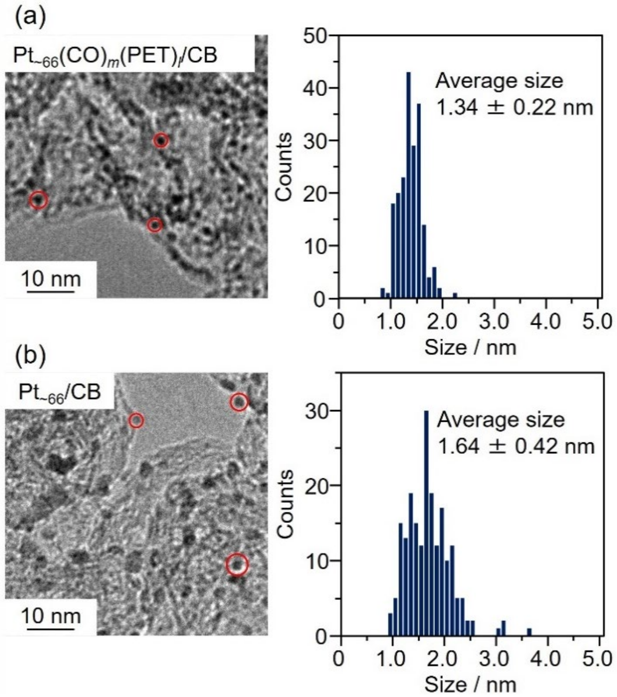

Figure S8. TEM images and the resulting Pt size histogram of Pt$_{\sim66}$ NCs after (a) adsorption on CB (Pt$_{\sim66}$(CO)$_m$(PET)$_l$/CB) and (b) calcination of Pt$_{\sim66}$(CO)$_m$(PET)$_l$/CB (Pt$_{\sim66}$/CB; the loading weight of Pt: 20.0 wt%).

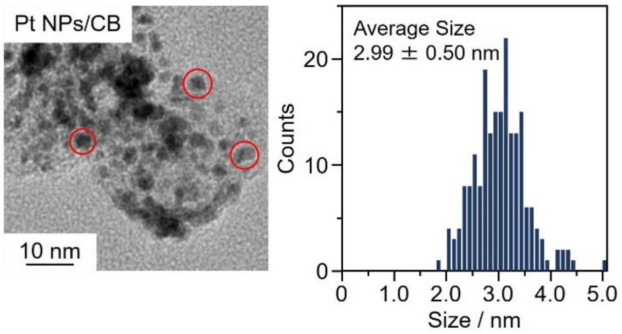

Figure S9. TEM images and the resulting histogram of commercial Pt NPs/CB (TEC10E50E; the loading weight of Pt: 46.9 wt%).

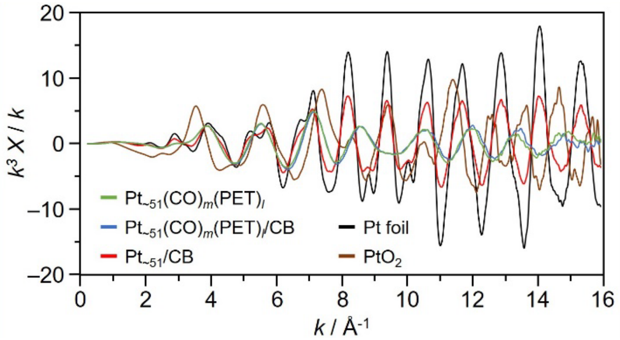

Figure S10. Pt L_3-edge EXAFS spectra of $Pt_{\sim51}(CO)_m(PET)_l$, $Pt_{\sim51}(CO)_m(PET)_l$/CB and $Pt_{\sim51}$/CB together with Pt foil and PtO_2 powder as reference.

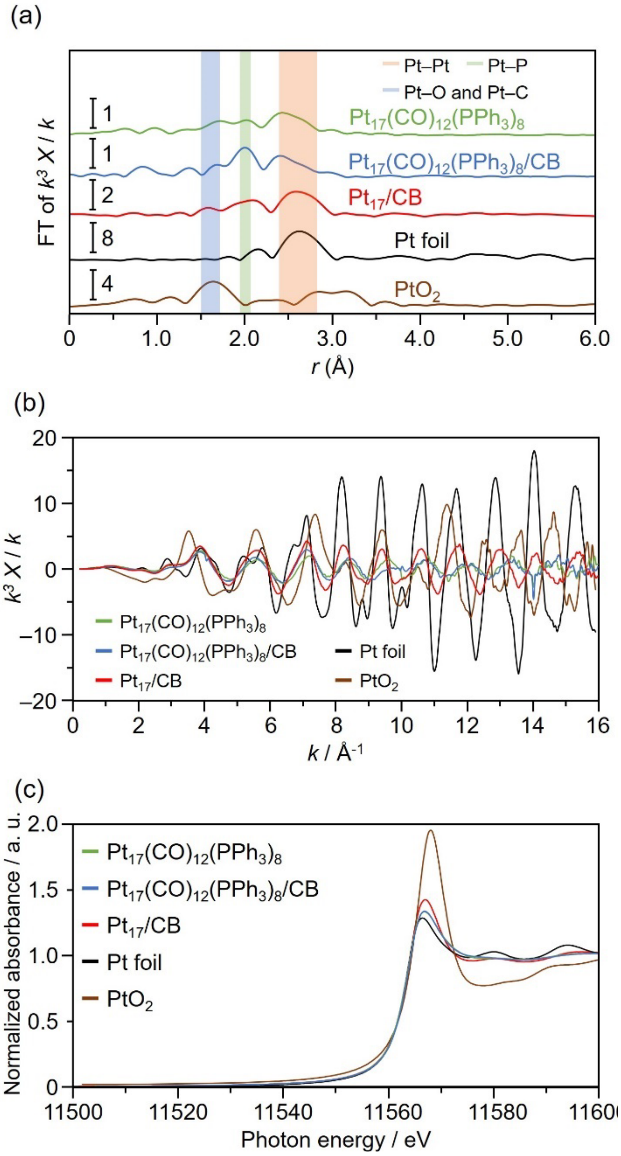

Figure S11. Pt L_3-edge (a) FT-EXAFS, (b) EXAFS and (c) XANES spectra of $Pt_{17}(CO)_{12}(PPh_3)_8$, $Pt_{17}(CO)_{12}(PPh_3)_8$/CB and Pt_{17}/CB together with Pt foil and PtO_2 powder as reference.

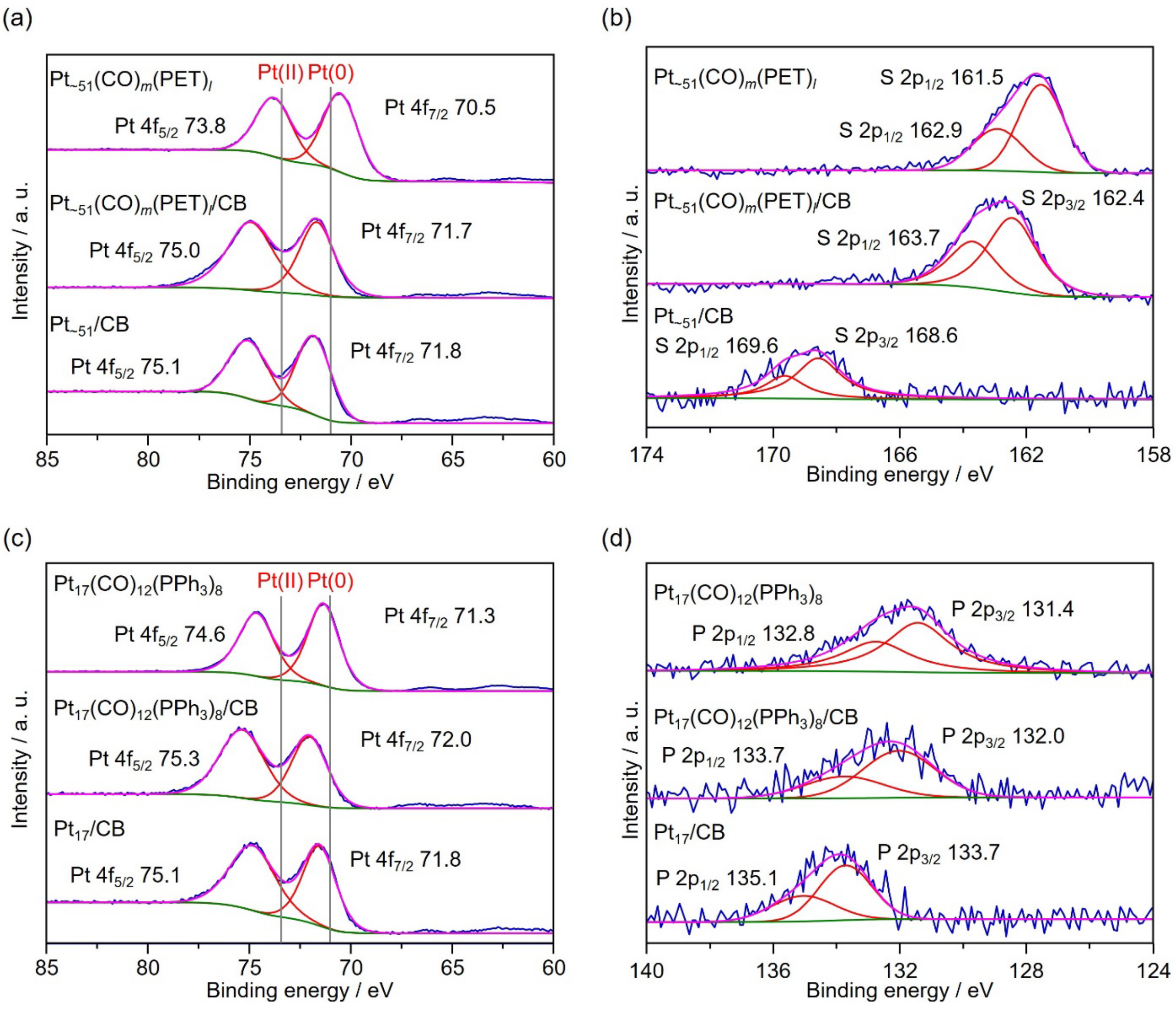

Figure S12. (a) Pt 4f and (b) S 2p XPS spectra of $Pt_{\sim 51}(CO)_m(PET)_l$, $Pt_{\sim 51}(CO)_m(PET)_l$/CB and $Pt_{\sim 51}$/CB. (c) Pt 4f and (d) P 2p XPS spectra of $Pt_{17}(CO)_{12}(PPh_3)_8$, $Pt_{17}(CO)_{12}(PPh_3)_8$/CB and Pt_{17}/CB.

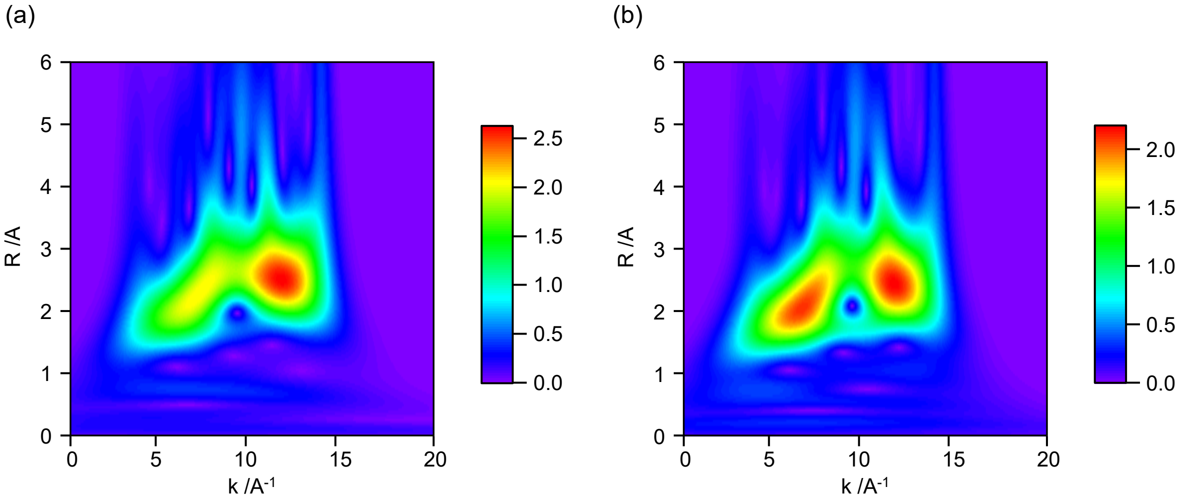

Figure S13. Wavelet transform (WT) images of Pt L_3-edge EXAFS spectra for (a) $Pt_{\sim51}$/CB and (b) Mel/$Pt_{\sim51}$/CB. WT analysis of the Pt L_3-edge successfully resolved the overlapping adsorption species that are difficult to distinguish in the conventional radial structure function. Specifically, the intense peak at r ~2.7 Å exhibited a maximum intensity in the higher wavenumber region (k > 10 $Å^{-1}$), which is a characteristic feature of Pt–Pt bonding involving heavy atom scattering. In contrast, the intensities observed at r ~1.6 Å and ~1.9 Å were clearly identified in the lower wavenumber region; the former is attributed to Pt–C/N scattering from light atoms, while the latter, appearing at a slightly higher k-range than the light atoms, is consistent with the Pt–S bond.

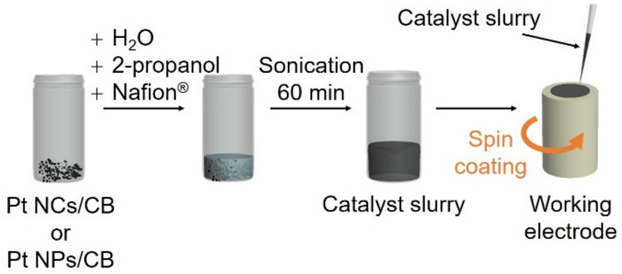

Figure S14. A schematic of the preparation method of catalyst slurry and working electrode.

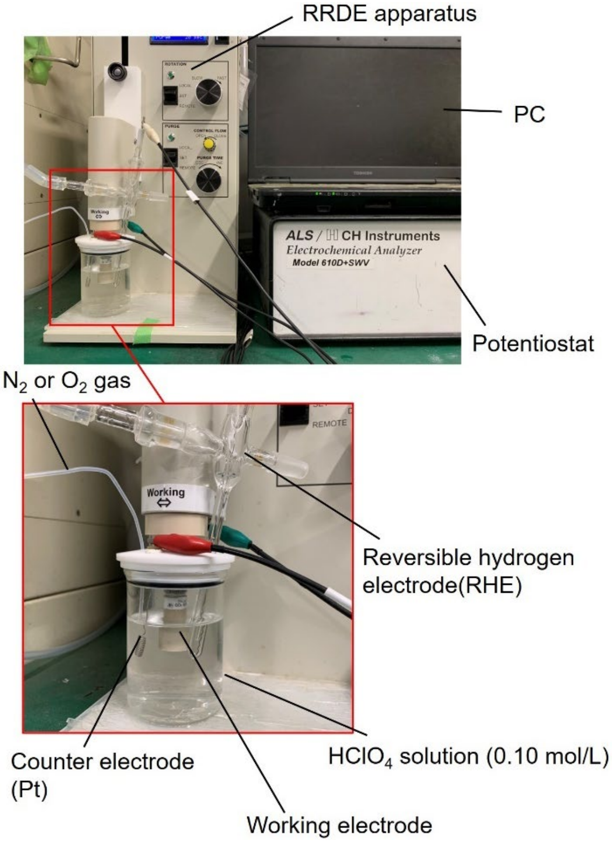

Figure S15. Photographs of the apparatus used in this work for electrochemical experiments. The setup modified from our previous study (reference electrode) is highlighted in red color. Reproduced with permission from Ref. 2. Copyright 2023, The Royal Society of Chemistry.

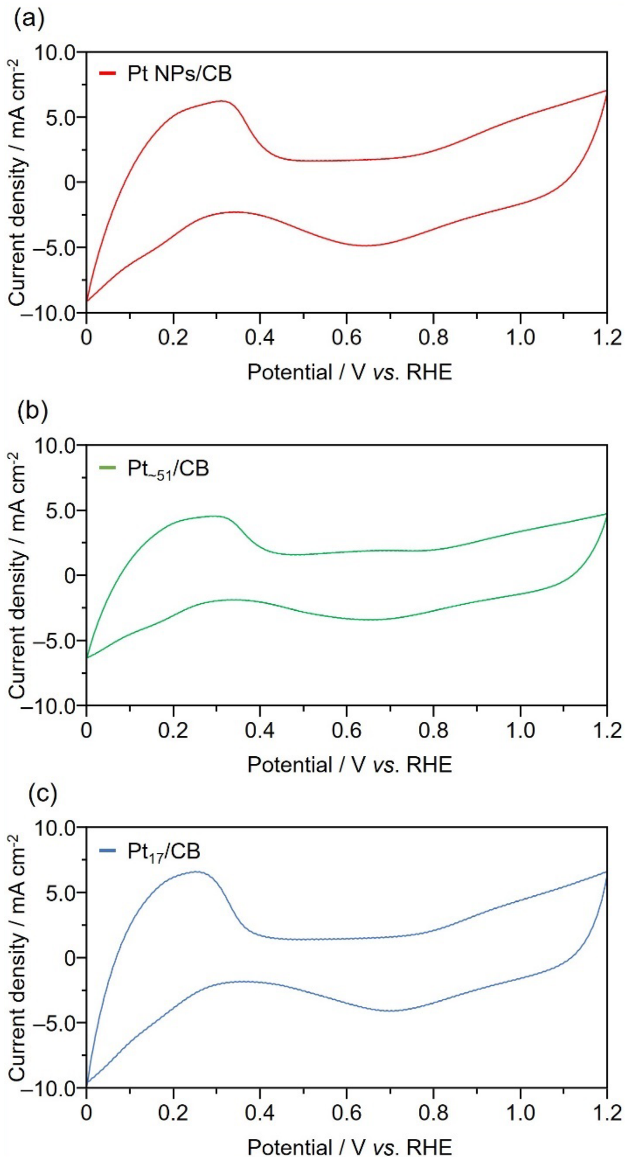

Figure S16. CV spectra after electrochemical cleaning of (a) Pt NPs/CB, (b) $Pt_{\sim 51}$/CB and c Pt_{17}/CB. The loading weight of Pt were 46.9, 20.0 and 20.0 wt% for Pt NPs/CB, $Pt_{\sim 51}$/CB and Pt_{17}/CB, respectively.

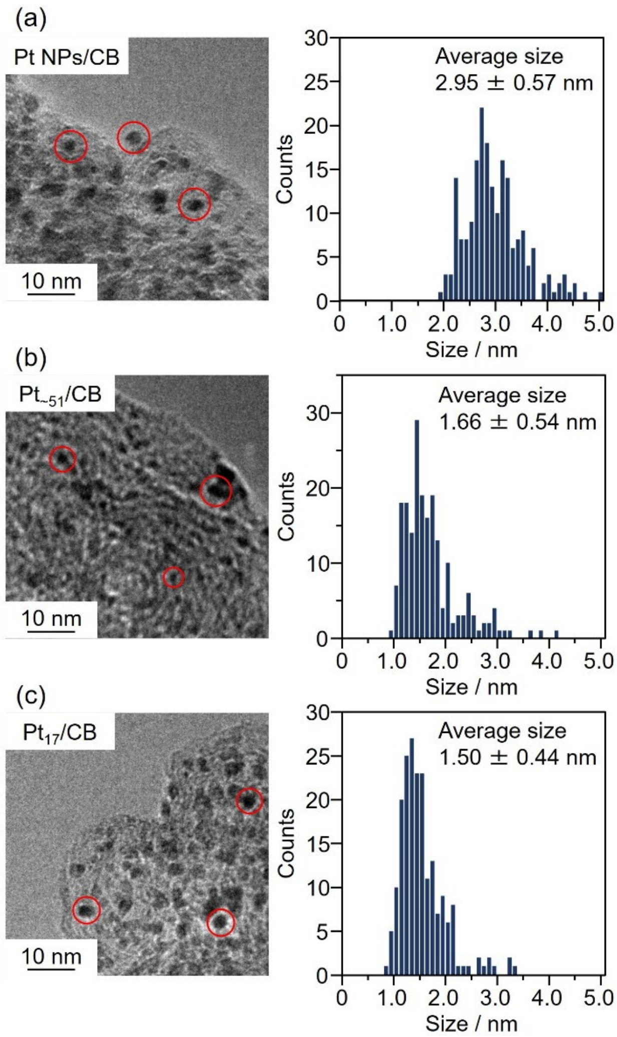

Figure S17. TEM images and the resulting Pt size histograms of (a) Pt NPs/CB, (b) $Pt_{\sim51}$/CB and (c) Pt_{17}/CB after CV cleaning. The loading weight of Pt were 46.9, 20.0 and 20.0 wt% for Pt NPs/CB, $Pt_{\sim51}$/CB and Pt_{17}/CB, respectively.

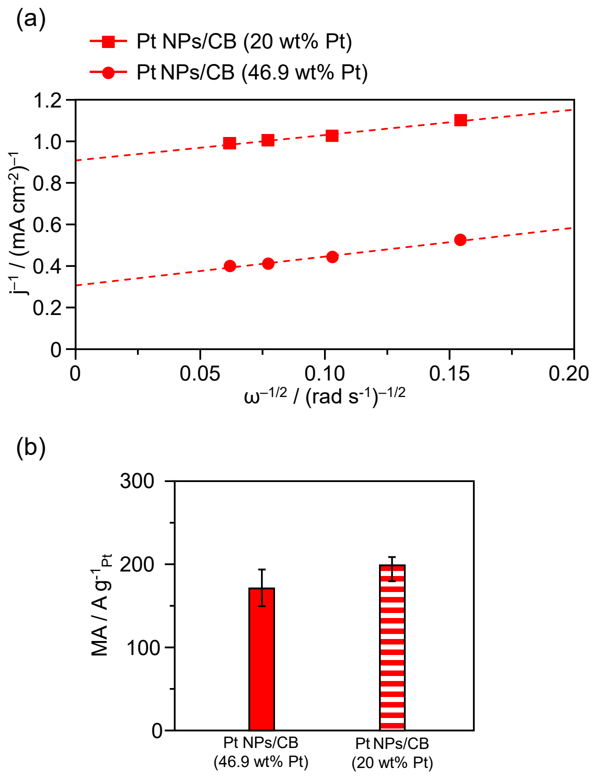

Figure S18. The obtained Koutecky−Levich plots and mass activities (MAs) of Pt NPs/CB(46.9 wt% Pt) and Pt NPs/CB(20.0 wt% Pt). The loading weight of Pt were 46.9 and 20.0 wt% for Pt NPs/CB(46.9 wt% Pt) and Pt NPs/CB(20.0 wt% Pt), respectively. The Pt loadings for Pt NPs/CB(46.9 wt% Pt) and Pt NPs/CB(20.0 wt% Pt) on the RDE were set to 17.8 and 5.05 μg/cm^2, respectively. Evaluations using Koutecky–Levich plots theoretically assume a smooth, planar electrode surface. In our case, the ORR activity of Pt_x/CB with a thicker catalyst layer (i.e., those with lower Pt weight content; 20.0 wt% Pt) may be underestimated. This is likely due to additional mass-transport resistance within the porous structure of the thicker film, which deviates from the ideal diffusion behavior assumed in the Koutecky−Levich model. However, within the range of Pt loading measured in this study, no significant difference in activity was observed even among commercially available Pt NPs/CB catalysts. This indicates that accurate mass activity comparisons can be made even across different Pt loadings from 20.0 wt% Pt to 46.9 wt%.

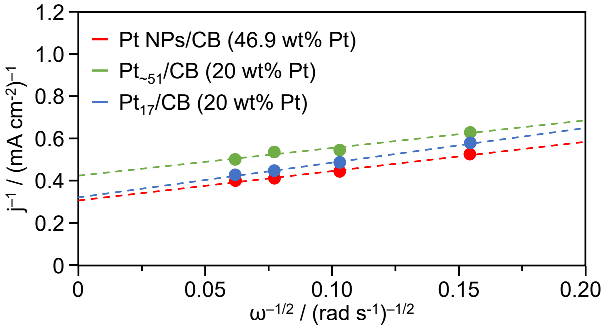

Figure S19. The obtained Koutecky−Levich plots of Pt NPs/CB, $Pt_{\sim 51}$/CB and Pt_{17}/CB. The loading weight of Pt were 46.9, 20.0 and 20.0 wt% for Pt NPs/CB, $Pt_{\sim 51}$/CB and Pt_{17}/CB, respectively. To ensure a uniform thickness of the CB layer, the Pt loadings for Pt NPs/CB and Pt_x/CB on the RDE were set to 17.8 and 5.05 μg/cm^2, respectively.

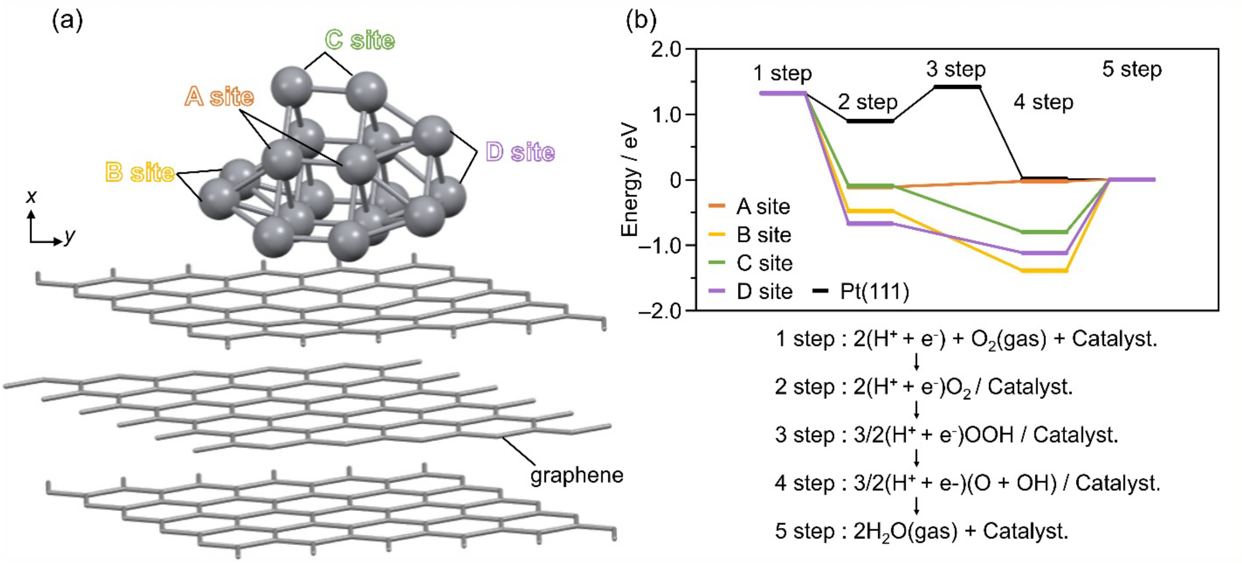

Figure S20. (a) Optimized structure for Pt_{17}/graphite(X) (X = A, B, C or D) and (b) free-energy diagram for ORR through a direct four-electron pathway on Pt_{17}/graphite(X) (X = A, B, C or D) or Pt(111) under the potential of 0.9 V vs. SHE. In (b), Pt_{17}/graphite(X) and Pt(111) are abbreviated as Catalyst. Reproduced with permission from Ref. 2. Copyright 2023, The Royal Society of Chemistry.

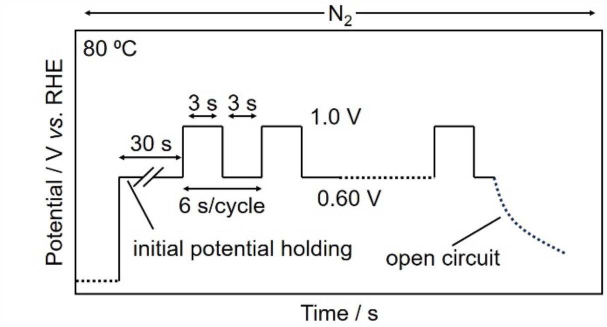

Figure S21. The protocol for the ADT of electrochemical measurements.

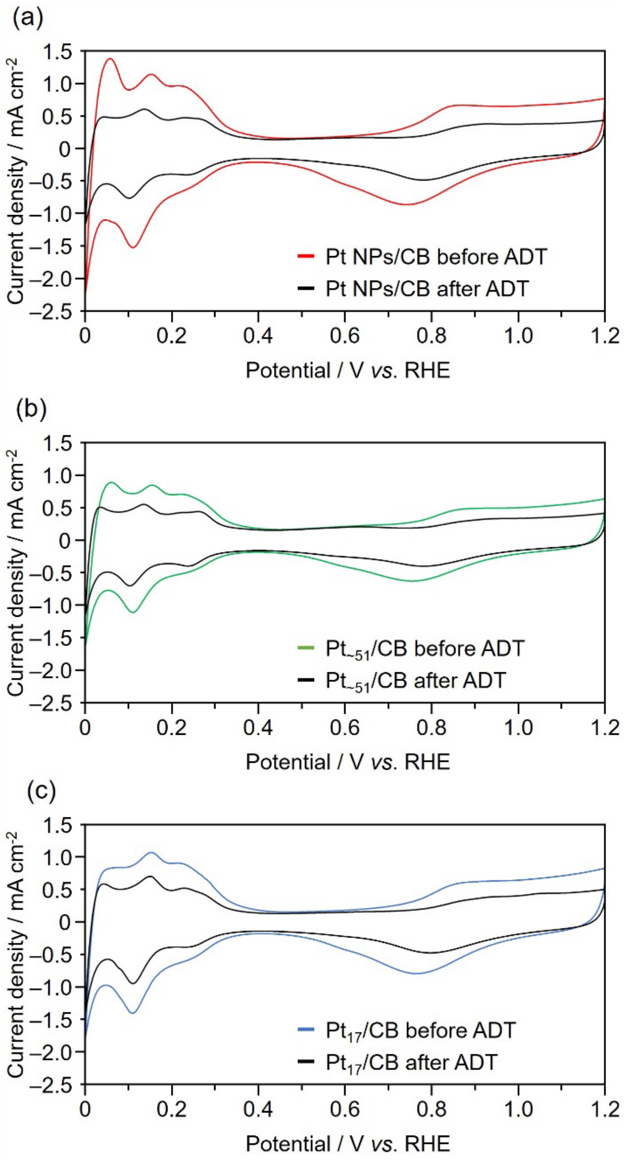

Figure S22. CVs before and after ADT of (a) Pt NPs/CB, (b) $Pt_{\sim 51}$/CB and c Pt_{17}/CB. The loading weight of Pt were 46.9, 20.0 and 20.0 wt% for Pt NPs/CB, $Pt_{\sim 51}$/CB and Pt_{17}/CB, respectively.

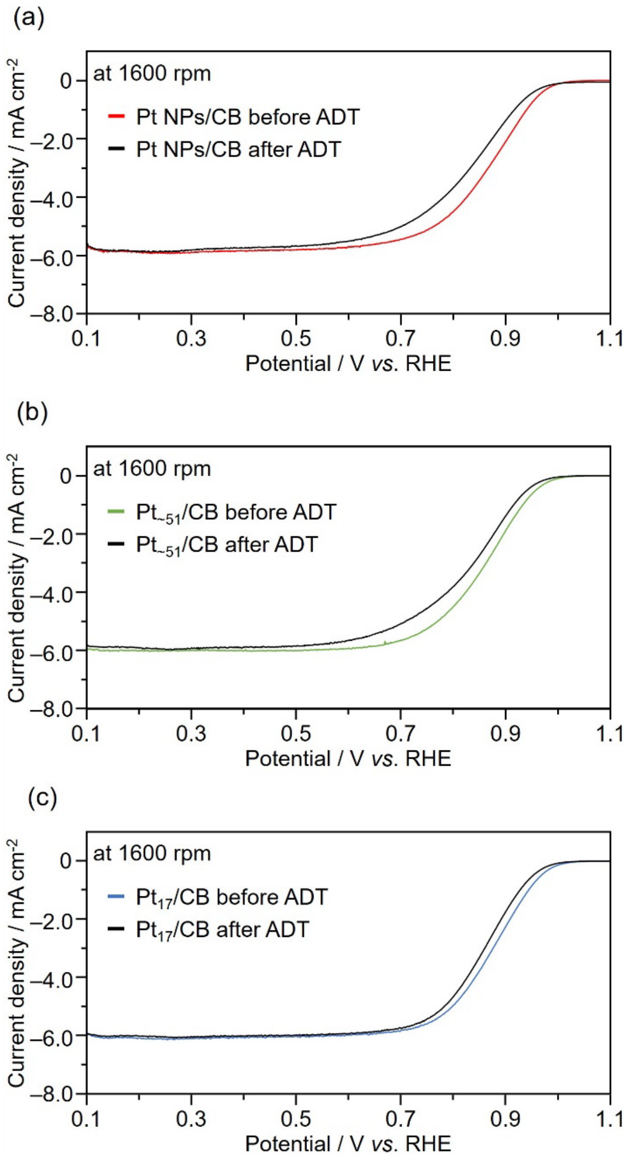

Figure S23. LSVs before and after ADT of (a) Pt NPs/CB, (b) $Pt_{\sim51}$/CB and (c) Pt_{17}/CB. The loading weight of Pt were 46.9, 20.0 and 20.0 wt% for Pt NPs/CB, $Pt_{\sim51}$/CB and Pt_{17}/CB, respectively.

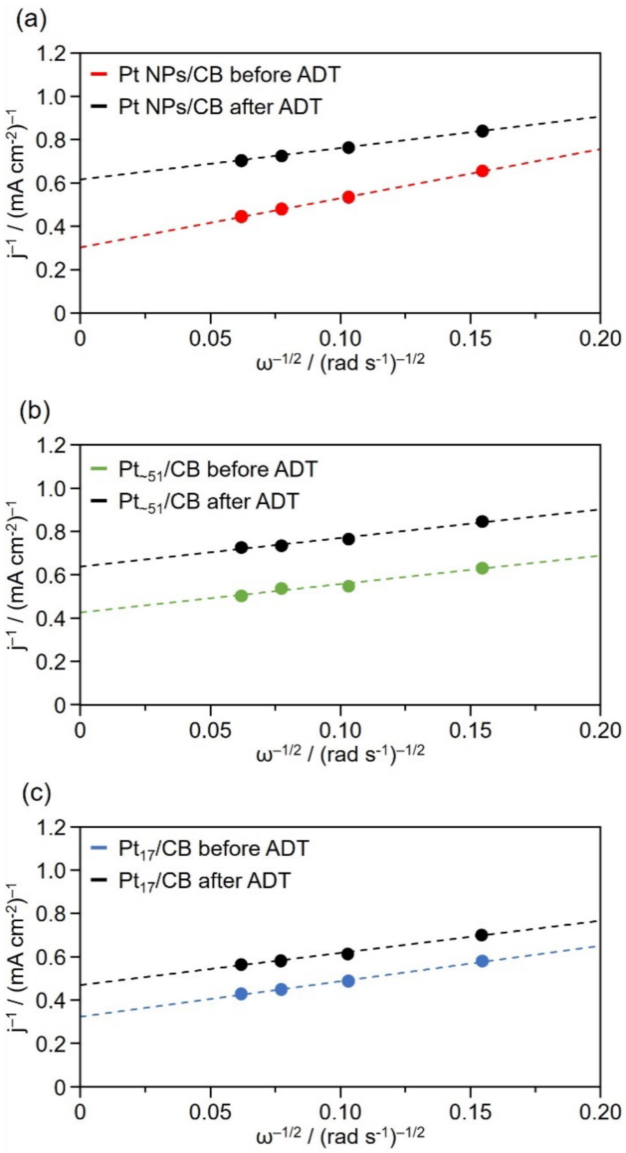

Figure S24. The obtained Koutecky−Levich plots of (a) Pt NPs/CB, (b) $Pt_{\sim 51}$/CB and (c) Pt_{17}/CB before and after ADT. The loading weight of Pt were 46.9, 20.0 and 20.0 wt% for Pt NPs/CB, $Pt_{\sim 51}$/CB and Pt_{17}/CB, respectively. To ensure a uniform thickness of the CB layer, the Pt loadings for Pt NPs/CB and Pt_x/CB on the RDE were set to 17.8 and 5.05 μg/cm^2, respectively.

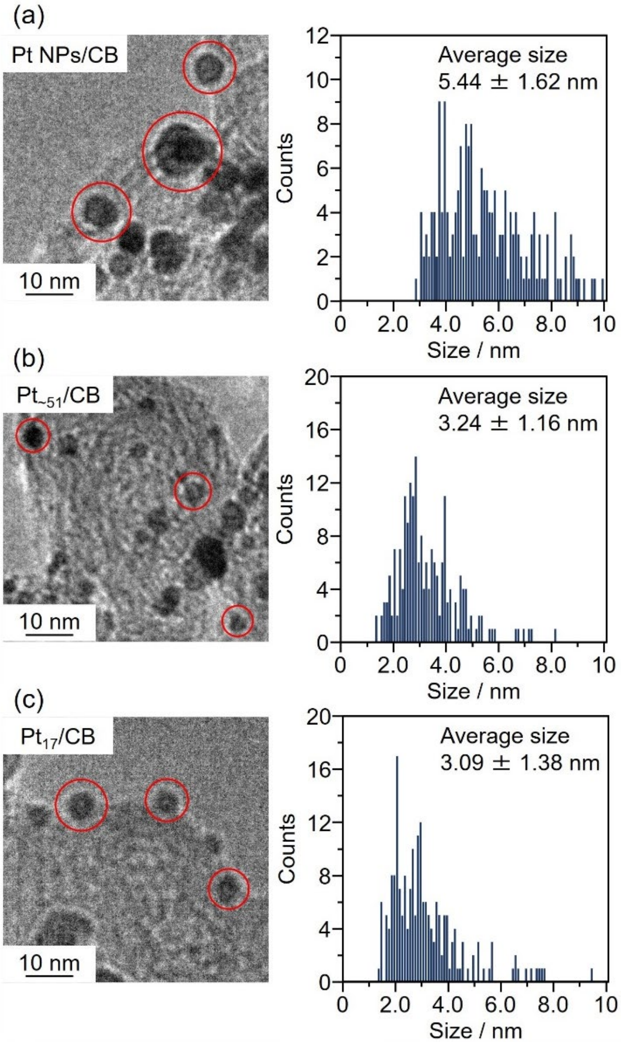

Figure S25. TEM images and the resulting Pt size histograms of (a) Pt NPs/CB, (b) $Pt_{\sim51}$/CB and (c) Pt_{17}/CB after ADT

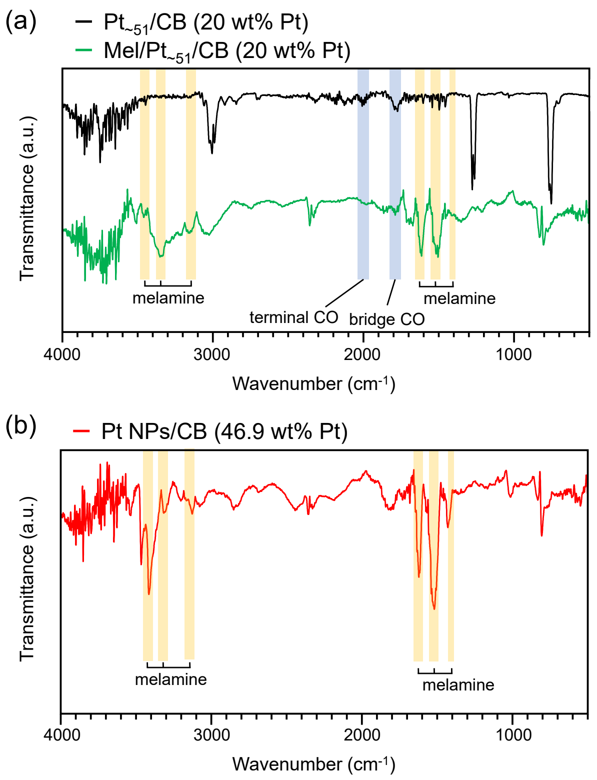

Figure S26. FT-IR spectra before and after melamine modification for (a) $Pt_{\sim 51}$/CB and (b) Pt NPs/CB. In (a), CO bond is attributed from the protective ligands of $Pt_{\sim 51}$ NCs in the synthesis process. These changes in the electronic states were observed under conditions of forced excess adsorption of melamine. At the ultra-trace adsorption levels associated with high activity, these signals remained below the detection limit of the current measurement system.

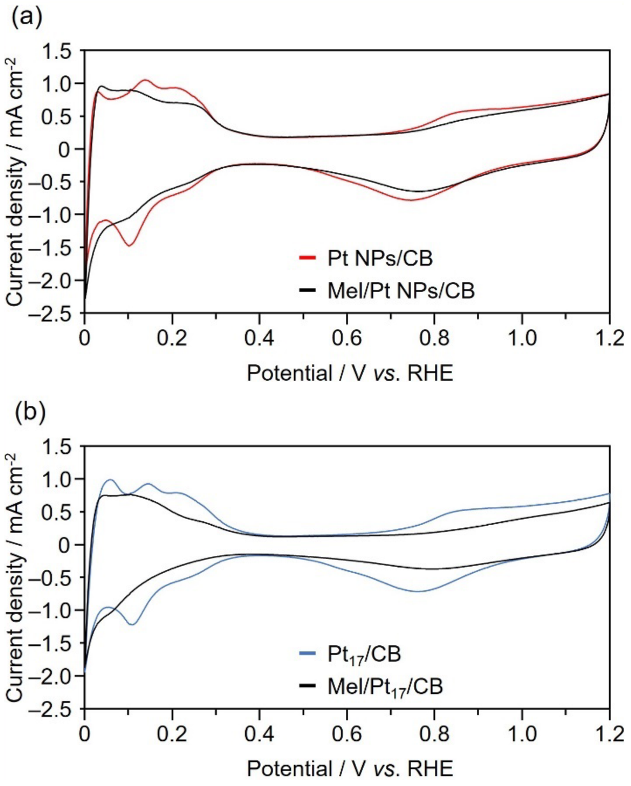

Figure S27. Comparison of CVs before and after melamine modification for (a) Pt NPs/CB and (b) Pt_{17}/CB. The loading weight of Pt were 46.9 and 20.0 wt% for Pt NPs/CB and Pt_{17}/CB, respectively.

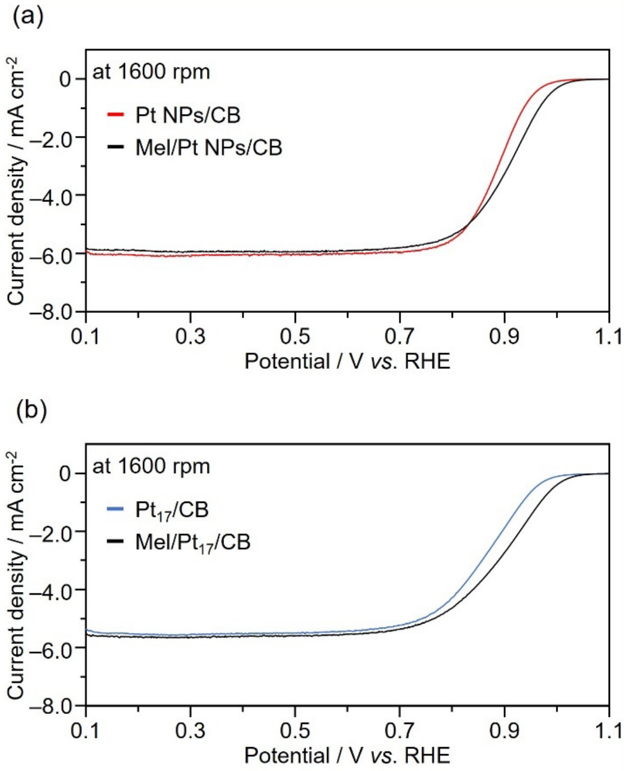

Figure S28. Comparison of LSVs before and after melamine modification for (a) Pt NPs/CB and (b) Pt_{17}/CB. The loading weight of Pt were 46.9 and 20.0 wt% for Pt NPs/CB and Pt_{17}/CB, respectively.

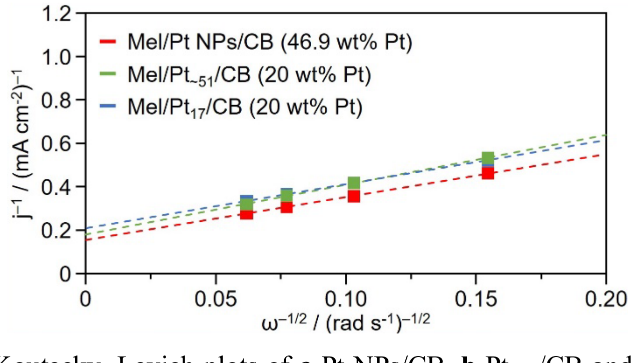

Figure S29. The obtained Koutecky−Levich plots of **a** Pt NPs/CB, **b** $Pt_{\sim 51}$/CB and **c** Pt_{17}/CB after melamine modification. The loading weight of Pt were 46.9, 20.0 and 20.0 wt% for Mel/Pt NPs/CB, Mel/$Pt_{\sim 51}$/CB and Mel/Pt_{17}/CB, respectively. To ensure a uniform thickness of the CB layer, the Pt loadings for Pt NPs/CB and Pt_x/CB on the RDE were set to 17.8 and 5.05 μg/cm^2, respectively.

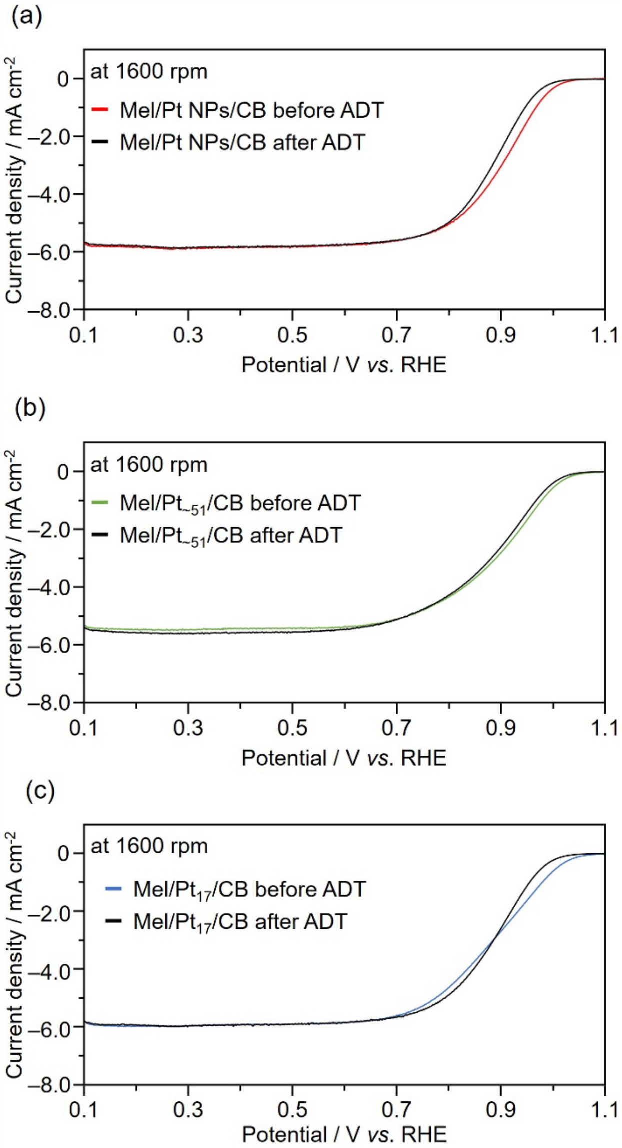

Figure S30. Comparison of LSVs before and after ADT for (a) Mel/Pt NPs/CB, (b) Mel/$Pt_{\sim51}$/CB and (c) Mel/Pt_{17}/CB. The loading weight of Pt were 46.9, 20.0 and 20.0 wt% for Mel/Pt NPs/CB, Mel/$Pt_{\sim51}$/CB and Mel/Pt_{17}/CB, respectively.

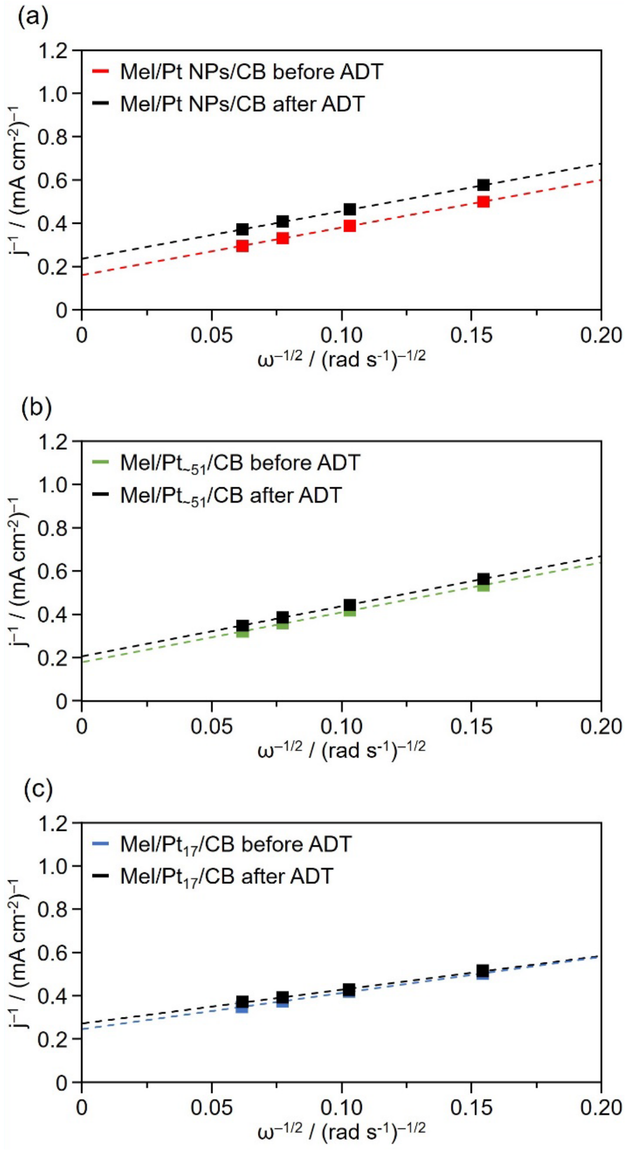

Figure S31. Koutecky−Levich plots before and after ADT (modification melamine) for (a) Mel/Pt NPs/CB, (b) Mel/$Pt_{\sim51}$/CB and (c) Mel/Pt_{17}/CB. The loading weight of Pt were 46.9, 20.0 and 20.0 wt% for Mel/Pt NPs/CB, Mel/$Pt_{\sim51}$/CB and Mel/Pt_{17}/CB, respectively. To ensure a uniform thickness of the CB layer, the Pt loadings for Pt NPs/CB and Pt_x/CB on the RDE were set to 17.8 and 5.05 μg/cm^2, respectively.

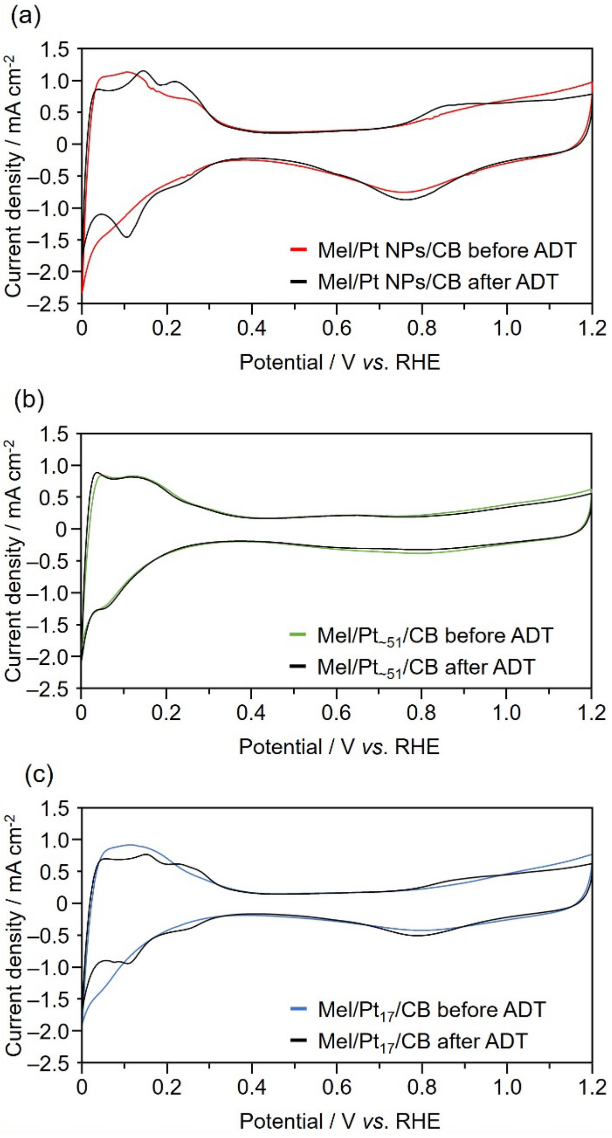

Figure S32. Comparison of CVs before and after ADT for (a) Mel/Pt NPs/CB, (b) Mel/Pt$_{\sim51}$/CB and (c) Mel/Pt$_{17}$/CB. The loading weight of Pt were 46.9, 20.0 and 20.0 wt% for Mel/Pt NPs/CB, Mel/Pt$_{\sim51}$/CB and Mel/Pt$_{17}$/CB, respectively.

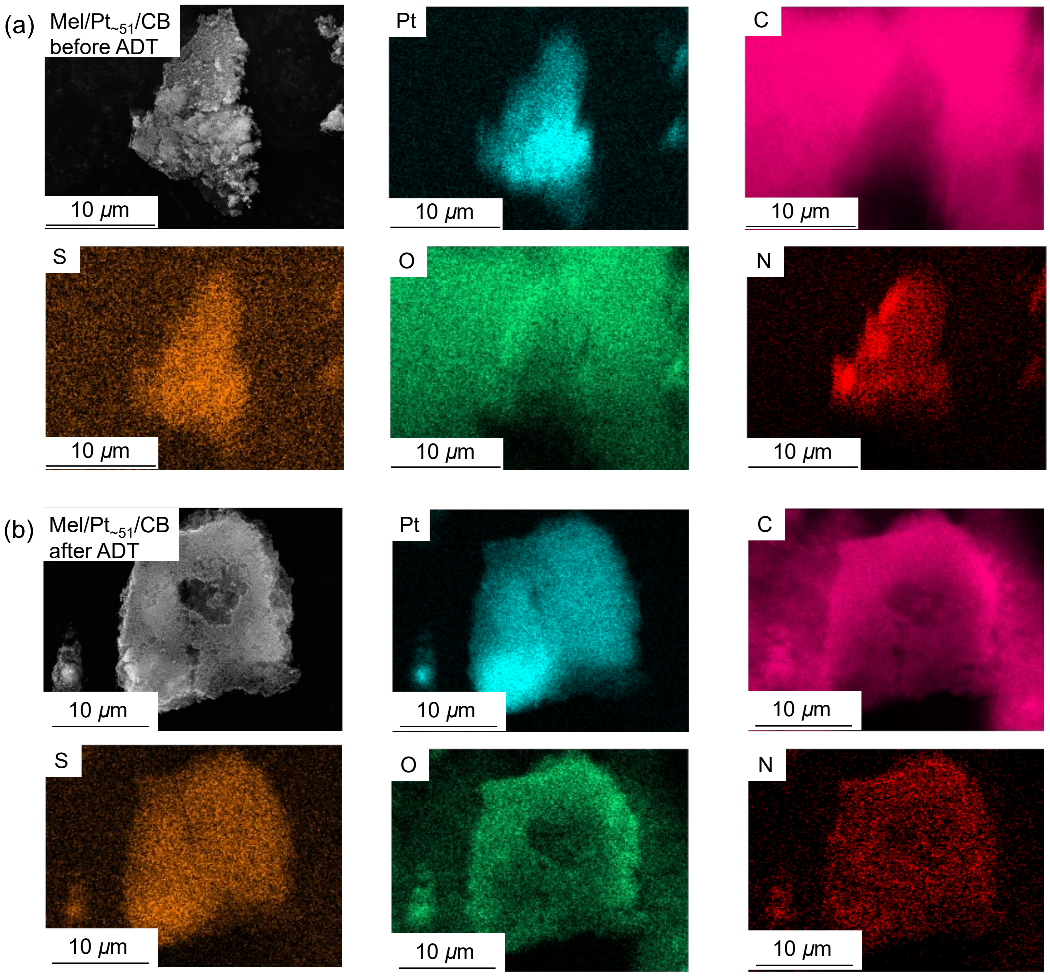

Figure S33. SEM images and energy-dispersive X-ray spectroscopy maps of Pt-M, C-K, O-K, S-K, and N-K of Mel/$Pt_{\sim 51}$/CB (a) before and (b) after ADT.

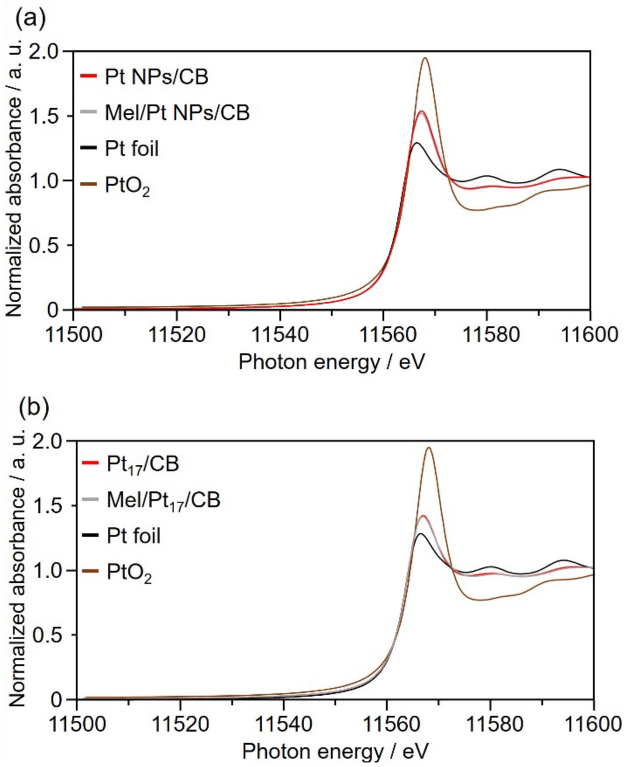

Figure S34. Pt L_3-edge XANES spectra before and after melamine modification for (a) Pt NPs/CB and (b) Pt_{17}/CB together with Pt foil and PtO_2 powder as reference. These changes in the electronic states were observed under conditions of forced excess adsorption of melamine. At the ultra-trace adsorption levels associated with high activity, these signals remained below the detection limit of the current measurement system.

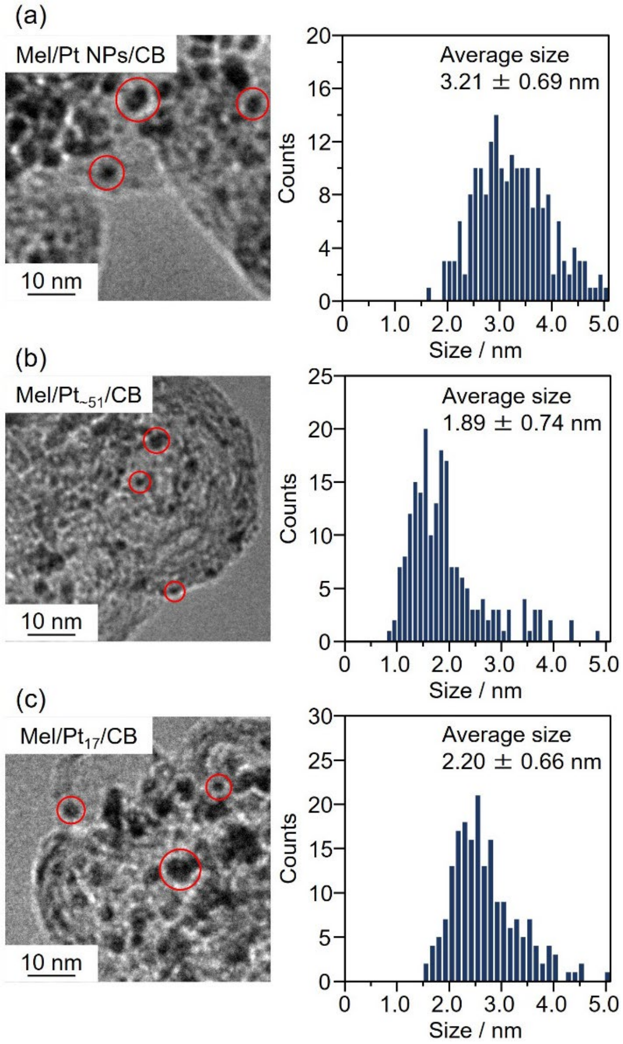

Figure S35. TEM images and the resulting Pt size histograms of (a) Mel/Pt NPs/CB, (b) Mel/$Pt_{\sim 51}$/CB and (c) Mel/Pt_{17}/CB after ADT.

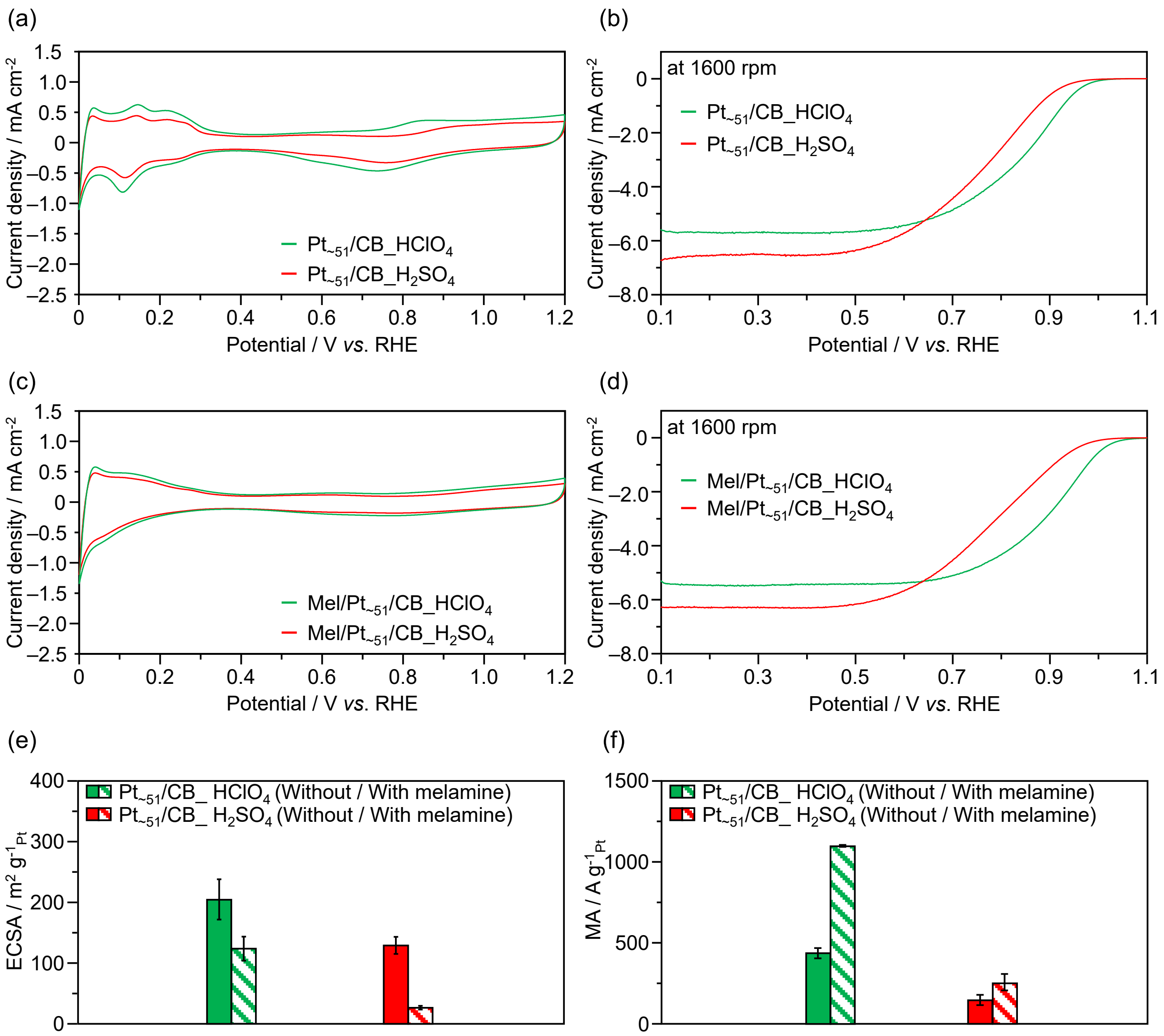

Figure S36. Comparison of electrochemical measurements in sulfuric acid or perchloric acid. Comparison of the (a, c) CVs and (b, d) LSVs for (a, b) Pt~51/CB and (c, d) Mel/Pt~51/CB in 0.1 M $HClO_4$ or H_2SO_4 aq. Resulting (e) ECSAs using proton adsorption current from CVs and (f) MAs calculated using the Koutecký–Levich plots from LSVs at 0.9 V *vs*. RHE.

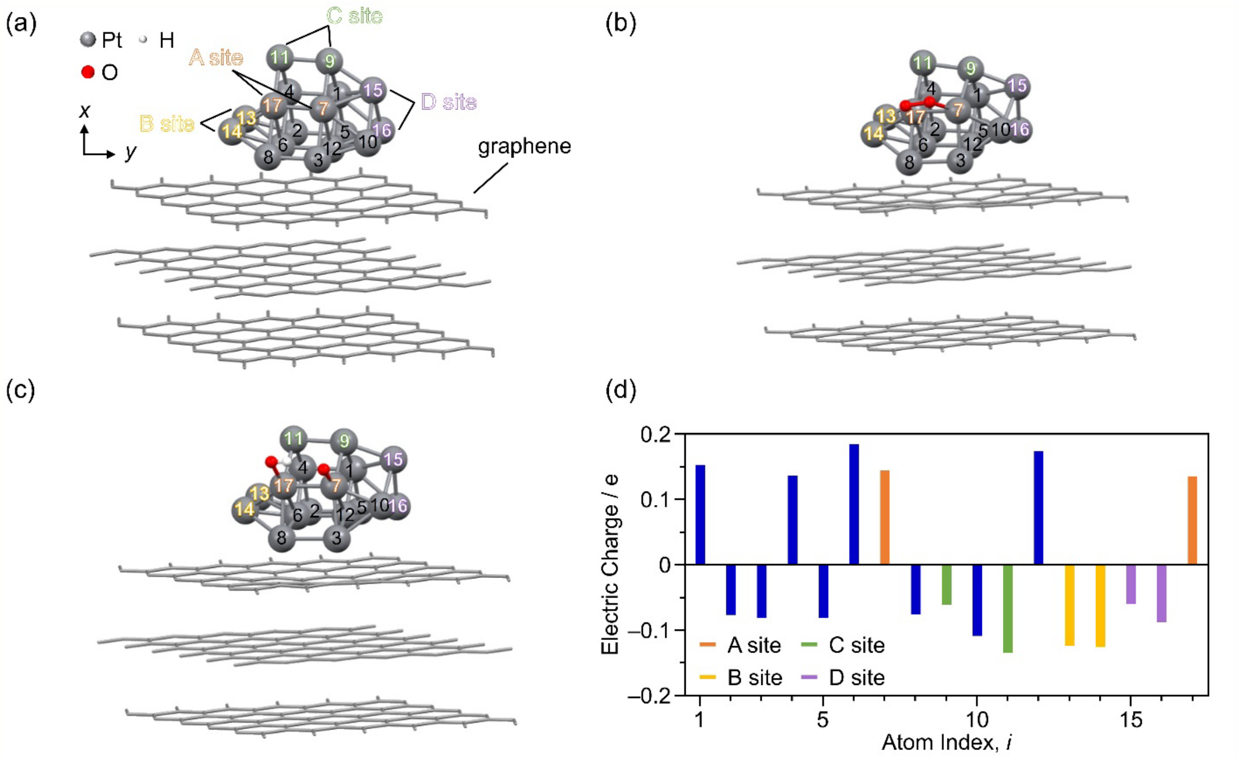

Figure S37. Results of DFT calculations. (a) Optimized structure of Pt_{17}/graphite. In this figure, the atom index (i) of each Pt atom and the site (A–D) for the reaction with O_2 are also described. (b) Optimized intermediate structure for O_2/Pt_{17}/graphite(A). (c) Optimized intermediate structure for (O + OH)/Pt_{17}/graphite(A). The other intermediate structures, O_2/Pt_{17}/graphite(X) and (O + OH)/Pt_{17}/graphite(X), obtained by starting Pt_{17}/graphite(X) (X = B, C, or D). (d) Electric charge of each Pt atom for Pt_{17}/graphite. Reproduced with permission from Ref. 2. Copyright 2023, The Royal Society of Chemistry.

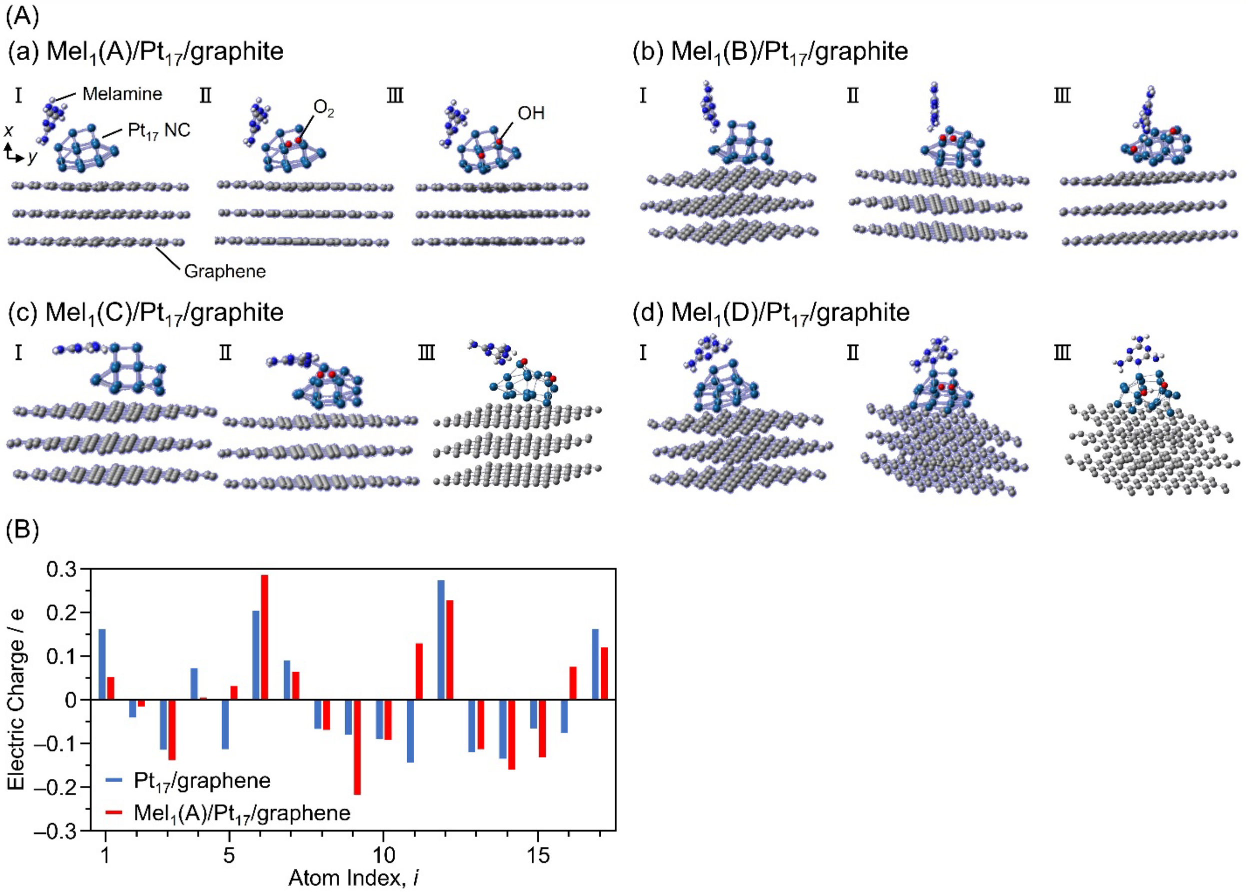

Figure S38. Results of DFT calculations. (A) (a–d) Optimized several Mel_1(X)/Pt_{17}/graphite (X = A–D) structure, where I, II and III correspond to the respective reaction steps in Figure 9(d). (B) Electric charge of each Pt atom for Pt_{17}/graphite and Mel_1(I)/Pt_{17}/graphite.

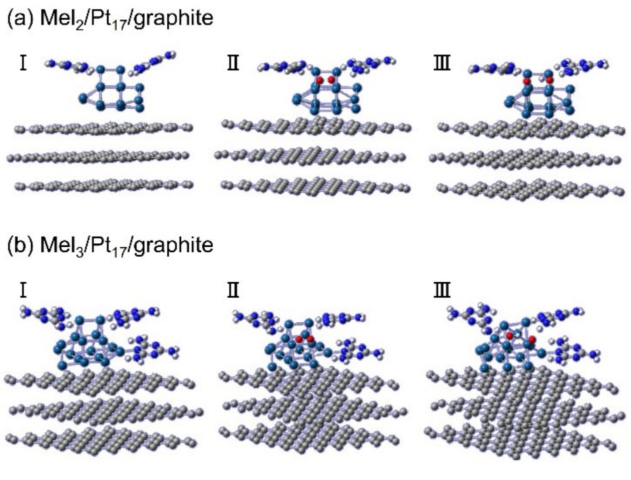

Figure S39. Results of DFT calculations. (a), Optimized MeI$_2$/Pt$_{17}$/graphite and (b) MeI$_3$/Pt$_{17}$/graphite structure, where I, II and III correspond to the respective reaction steps in Figure 9(d).

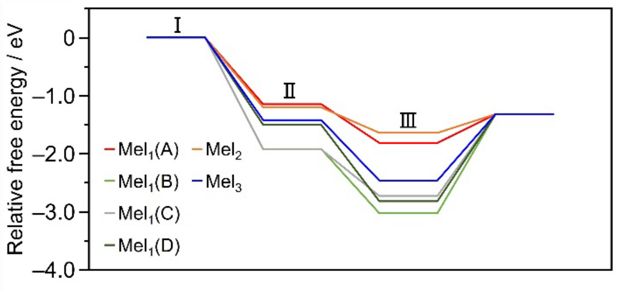

I : 2(H⁺ + e⁻) + O₂ / Catalyst.
II : 2(H⁺ + e⁻)O₂ / Catalyst.
III : 3/2(H⁺ + e-)(O + OH) / Catalyst.

Figure S40. Free-energy diagram for ORR through a direct four-electron pathway on Mel$_x$(X)/Pt_{17}/graphite (x = 1–3) under the potential of 0.9 V vs. SHE, where I, II and III correspond to [2(H^+ + e^-) + O_2(gas) + Mel$_x$(X)/Pt_{17}/graphite], [2(H^+ + e^-)O_2/Mel$_x$(X)/Pt_{17}/graphite] and [3/2(H^+ + e^-)(O+OH)/Mel$_x$(X)/Pt_{17}/graphite]. This diagram is derived from the optimized structure in Figures S33 and 34.

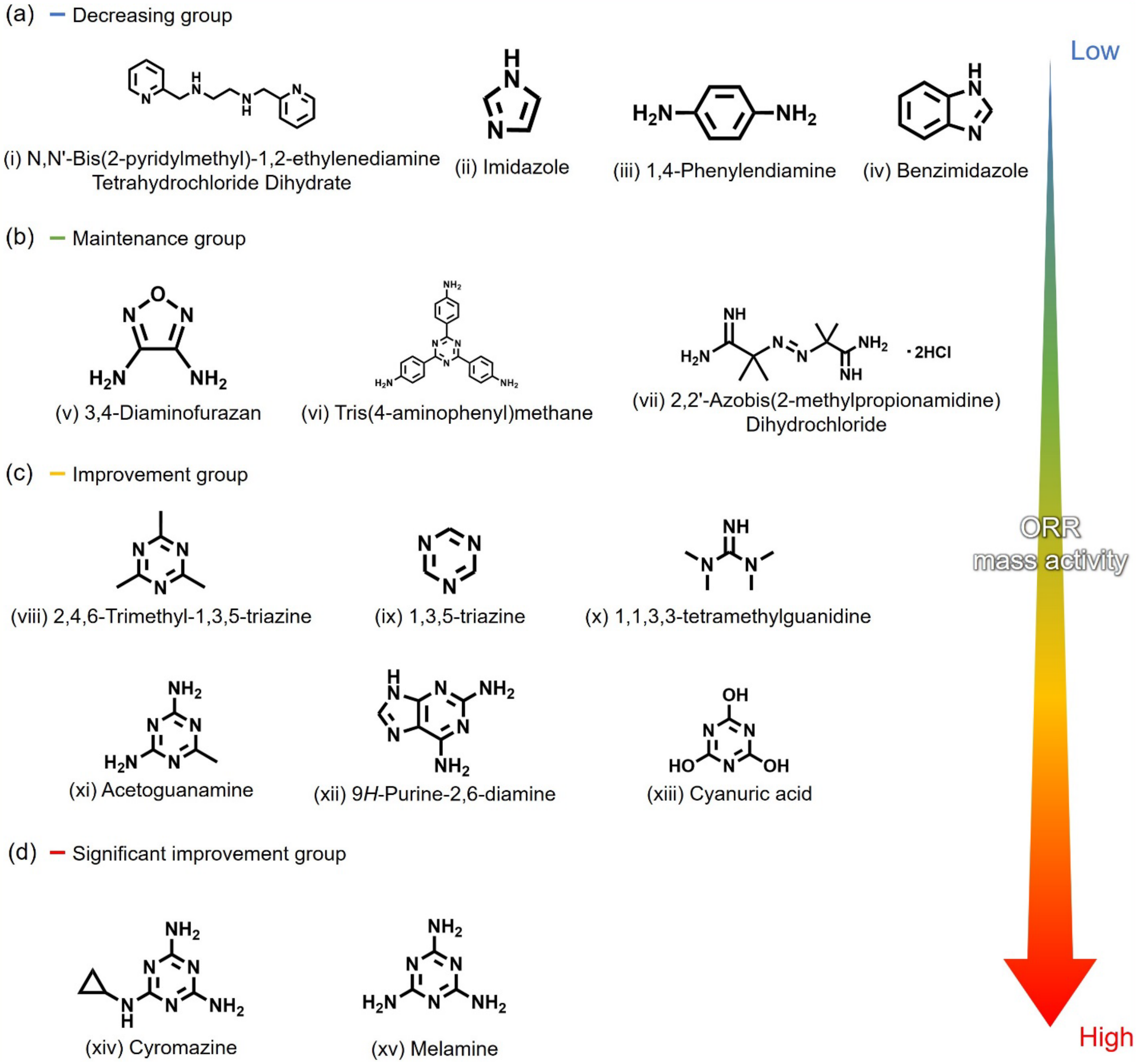

Figure S41. Organic chemicals for the modification of $Pt_{\sim 51}$/CB. Organic chemicals with (a) reduced MA, (b) maintained MA, (c) improved MA and (d) significantly improved MA.

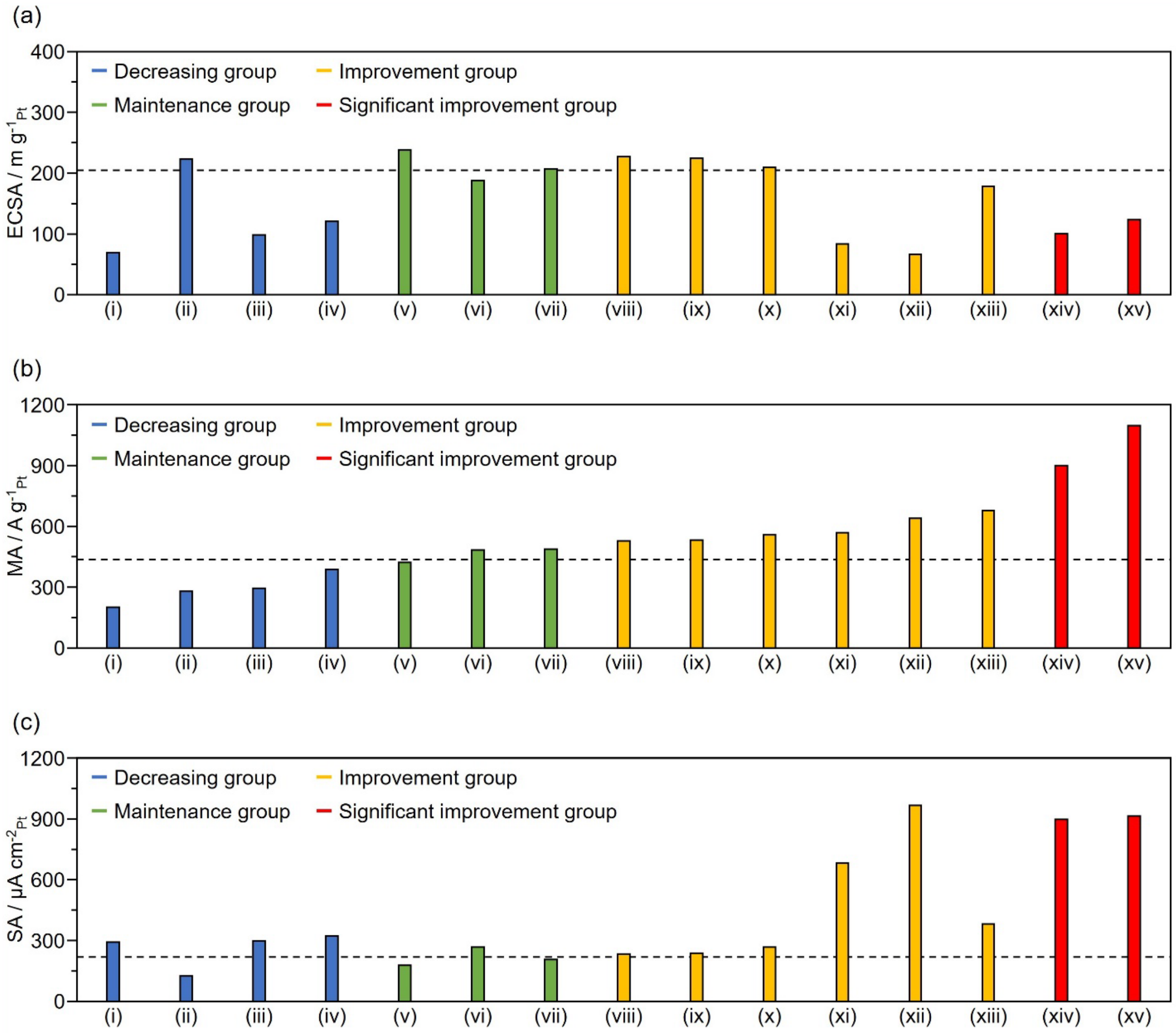

Figure S42. Results of electrochemical measurements of the organic chemicals modified $Pt_{\sim51}$/CB. Comparison of (a) ECSAs calculated from CVs, (b) MAs calculated from LSVs and **c** SA calculated from ECSA and MA. (i)−(xv) correspond to Figure. S36. The loading weight of Pt was 20.0 wt%.

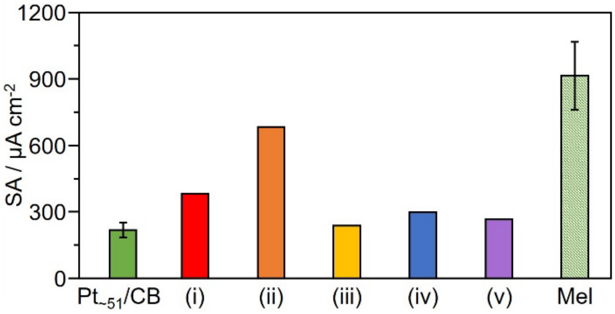

Figure S43. Comparison of SA calculated from ECSA and MA for (i) cyanuric acid, (ii) acetoguanamine, (iii) 1,3,5–triazine, (iv) 1,4–phenylenediamine, (v) 4,4',4''-(1,3,5-triazine-2,4,6-triyl)trianiline and (Mel) melamine modified Pt~51~/CB. The loading weight of Pt was 20.0 wt%.

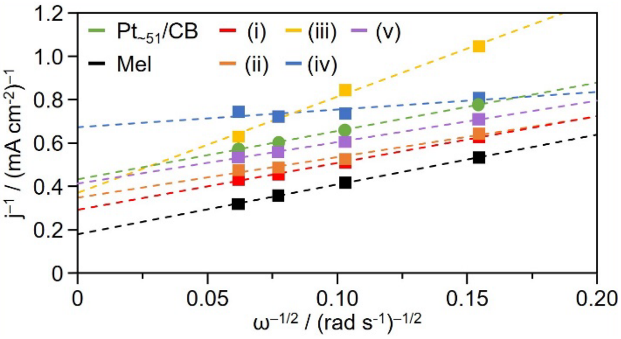

Figure S44. Comparison of Koutecky−Levich plots for (i) cyanuric acid, (ii) acetoguanamine, (iii) 1,3,5–triazine, (iv) 1,4–phenylenediamine, (v) 4,4',4''-(1,3,5-triazine-2,4,6-triyl)trianiline and (Mel) melamine modified Pt~51~/CB. The loading weight of Pt was 20.0 wt%.

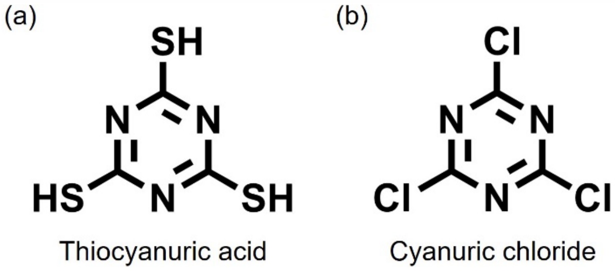

Figure S45. The structures of 1,3,5-triazine derivatives with low water solubility, (a) thiocyanuric acid and (b) cyanuric chloride. Electrochemical evaluation of the working electrode immersed in aqueous solutions of 1,3,5-triazine derivatives containing relatively strong electron-donating thiol (–SH) and chloro (–Cl) groups (thiocyanuric acid and cyanuric chloride) was also attempted. However, owing to their poor solubility in water, a proper evaluation could not be performed.

S8. References

1. Kawawaki, T.; Shimizu, N.; Funai, K.; Mitomi, Y.; Hossain, S.; Kikkawa, S.; Osborn, D. J.; Yamazoe, S.; Metha, G. F.; Negishi, Y. Simple and high-yield preparation of carbon-black-supported ~1 nm platinum nanoclusters and their oxygen reduction reactivity. *Nanoscale* **2021**, *13* (35), 14679–14687. DOI: 10.1039/D1NR04202E
2. Kawawaki, T.; Mitomi, Y.; Nishi, N.; Kurosaki, R.; Oiwa, K.; Tanaka, T.; Hirase, H.; Miyajima, S.; Niihori, Y.; Osborn, D. J.; Koitaya, T.; Metha, G. F.; Yokoyama, T.; Iida, K.; Negishi, Y. Pt_{17} nanocluster electrocatalysts: preparation and origin of high oxygen reduction reaction activity. *Nanoscale* **2023**, *15* (16), 7272–7279. DOI: 10.1039/D3NR01152F
3. Nair, L. V.; Hossain, S.; Wakayama, S.; Takagi, S.; Yoshioka, M.; Maekawa, J.; Harasawa, A.; Kumar, B.; Niihori, Y.; Kurashige, W.; Negishi, Y. $[Pt_{17}(CO)_{12}(PPh_3)_8]^{n+}$ (n=1, 2): Synthesis and geometric and electronic structures. *J. Phys. Chem. C* **2017**, *121* (20), 11002–11009. DOI: 10.1021/acs.jpcc.7b00978
4. Yazaki, D.; Kawawaki, T.; Hirayama, D.; Kawachi, M.; Kato, K.; Oguchi, S.; Yamaguchi, Y.; Kikkawa, S.; Ueki, Y.; Hossain, S.; Osborn, D. J.; Ozaki, F.; Tanaka, S.; Yoshinobu, J.; Metha, G. F.; Yamazoe, S.; Kudo, A.; Yamakata, A.; Negishi, Y. Carbon Nitride Loaded with an Ultrafine, Monodisperse, Metallic Platinum-Cluster Cocatalyst for the Photocatalytic Hydrogen-Evolution Reaction. *Small* **2023**, *19* (34), 2208287. DOI: 10.1002/smll.202208287
5. Fang, J.; Li, J.; Zhang, B.; Yuan, X.; Asakura, H.; Tanaka, T.; Teramura, K.; Xie, J.; Yan, N. The support effect on the size and catalytic activity of thiolated Au_{25} nanoclusters as precatalysts. *Nanoscale* **2015**, *7* (14), 6325–6333. DOI: 10.1039/C5NR00549C
6. Garsany, Y.; Singer, I. L.; Swider-Lyons, K. E. Impact of film drying procedures on RDE characterization of Pt/VC electrocatalysts. *J. Electroanal. Chem.* **2011**, *662* (2), 396–406. DOI: 10.1016/j.jelechem.2011.09.016
7. Ohma, A.; Shinohara, K.; Iiyama, A.; Yoshida, T.; Daimaru, A. Membrane and Catalyst Performance Targets for Automotive Fuel Cells by FCCJ Membrane, Catalyst, MEA WG. *ECS Trans.* **2011**, *41* (1), 775–784. DOI: 10.1149/1.3635611
8. Elgrishi, N.; Rountree, K. J.; McCarthy, B. D.; Rountree, E. S.; Eisenhart, T. T.; Dempsey, J. L. A Practical Beginner's Guide to Cyclic Voltammetry. *J. Chem. Educ.* **2018**, *95* (2), 197–206. DOI: 10.1021/acs.jchemed.7b00361
9. Cattabriga, E.; Ciabatti, I.; Femoni, C.; Funaioli, T.; Iapalucci, M. C.; Zacchini, S. Syntheses, Structures, and Electrochemistry of the Defective ccp $[Pt_{33}(CO)_{38}]^{2-}$ and the bcc $[Pt_{40}(CO)_{40}]^{6-}$ Molecular Nanoclusters. *Inorg. Chem.* **2016**, *55* (12), 6068–6079. DOI: 10.1021/acs.inorgchem.6b00607
10. Marković, N. M.; Gasteiger, H. A.; Grgur, B. N.; Ross, P. N. Oxygen reduction reaction on Pt(111): effects of bromide. *J. Electroanal. Chem.* **1999**, *467* (1–2), 157–163. DOI: 10.1016/S0022-0728(99)00020-0
11. Predew, J. P.; Burke, K.; Ernzerhof, M. Generalized Gradient Approximation Made Simple. *Phys. Rev. Lett.* **1996**, *77* (18), 3865–3868. DOI: 10.1103/PhysRevLett.77.3865
12. Giannozzi, P.; Baroni, S.; Bonini, N.; Calandra, M.; Car, R.; Cavazzoni, C.; Ceresoli, D.; Chiarotti, G. L.; Cococcioni, M.; Dabo, I.; Corso, A. D.; Gironcoli, S.; Fabris, S.; Fratesi, G.; Gebauer, R.; Gerstmann, U.; Gougoussis, C.; Kokalj, A.; Lazzeri, M.; Martin-Samos, L.; Marzari, N.; Mauri, F.; Mazzarello, R.; Paolini, S.;

Pasquarello, A.; Paulatto, L.; Sbraccia, C.; Scandolo, S.; Sclauzero, G.; Seitsonen, A. P.; Smogunov, A.; Umari, P.; Wentzcovitch, R. M. QUANTUM ESPRESSO: a modular and open-source software project for quantum simulations of materials. *J. Phys.: Condens. Matter* **2009**, *21* (39), 395502. DOI: 10.1088/0953-8984/21/39/395502

13. Grimme, S.; Antony, J.; Ehrlich, S.; Krieg, H. A consistent and accurate ab initio parametrization of density functional dispersion correction (DFT-D) for the 94 elements H-Pu. *J. Chem. Phys.* **2010**, *132* (15), 154104. DOI: 10.1063/1.3382344
14. Nørskov, J. K.; Rossmeisl, J.; Logadottir, A.; Lindqvist, L.; Kitchin, J. R.; Bligaard, T.; Jónsson, H. Origin of the Overpotential for Oxygen Reduction at a Fuel-Cell Cathode. *J. Phys. Chem. B* **2004**, *108* (46), 17886–17892. DOI: 10.1021/jp047349j
15. Rossmeisl, J.; Logadottir, A.; Nørskov, J. K. Electrolysis of water on (oxidized) metal surfaces. *Chem. Phys.* **2005**, *319* (1–3), 178–184. DOI 10.1016/j.chemphys.2005.05.038
16. Lim, D.-H.; Wilcox, J. Mechanisms of the Oxygen Reduction Reaction on Defective Graphene-Supported Pt Nanoparticles from First-Principles. *J. Phys. Chem. C* **2012**, *116* (5), 3653–3660. DOI: 10.1021/jp210796e
